# Supplementary material for: Patterns of brain atrophy in recently-diagnosed relapsing-remitting multiple sclerosis
Source: PLoS One. 2023 Jul 28;18(7):e0288967. doi: 10.1371/journal.pone.0288967 (PMC10381059; doi:10.1371/journal.pone.0288967)
Supplement: S1 Table — (DOCX) [file pone.0288967.s001.docx]

SUPPLEMENT

**S1 Table. Future MS MRI parameters for protocol A and B.**

| A. PROTOCOL A | | | | | | | | | | | | |
| --- | --- | --- | --- | --- | --- | --- | --- | --- | --- | --- | --- | --- |
| Sequence | T1-weighted | | | | T2-weighted | | | | 2D FLAIR | | | |
| Site | **EDI1** | **GLA** | **DUN** | **ABN** | **EDI1** | **GLA** | **DUN** | **ABN** | **EDI1** | **GLA** | **DUN** | **ABN** |
| Mode | 3D | 3D | 3D | 3D | 2D | 2D | 2D | 3D | 2D | 2D | 2D | 2D |
| FOV (mm) | 256 | 256 | 256 | 240 | 220 | 220 | 220 | 256 | 250 | 250 | 250 | 250 |
| Orientation | Sag | Sag | Sag | Sag | Ax | Ax | Ax | Sag | Ax | Ax | Ax | Ax |
| TR (ms) | 2530 | 2500 | 2500 | 3000 | 6000 | 6160 | 6160 | 2500 | 9500 | 9500 | 9500 | 11000 |
| TE (ms) | 3.37 | 2.26 | 2.26 | 3.9 | 96 | 96 | 96 | 310 | 124 | 124 | 124 | 125 |
| TI (ms) | 1100 | 1100 | 1100 | 1048 | - | - | - | - | 2400 | 2400 | 2400 | 2800 |
| Flip angle (deg) | 7 | 7 | 7 | 8 | 150 | 150 | 150 | - | 150 | 150 | 150 | 120 |
| Gap (mm) | - | - | - | - | 1.2 | 1.2 | 1.2 | - | 0 | 0 | 0 | 1 |
| Matrix (mm) | 256 × 256 | 256 × 256 | 256 × 256 | 240 × 240 | 320 x 320 | 320 × 314 | 314 x 314 | 256 x 256 | 256 x 256 | 256 × 256 | 256 x 256 | 252 x 226 |
| Voxel size (mm) | 1 × 1 × 1 | 1 × 1 × 1 | 1 × 1 × 1 | 1 x 1 x 1 | 0.7 × 0.7 × 4 | 0.7 × 0.7 × 4 | 0.7 × 0.7 × 4 | 1 x 1 x 2 | 1 x 1 x 3 | 1 × 1 × 3 | 1 x 1 x 3 | 1 x 1.1 x 3 |
| Slices reconstructed | 176 | 176 | 176 | 160 | 33 | 33 | 33 | 176 | 60 | 60 | 60 | 29 |
| Acq. Time (m:ss) | 6:03 | 5:59 | 5:59 | 5:38 | 1:26 | 1:03 | 1:03 | 3:42 | 7:38 | 7:38 | 7:38 | 5:08 |
|  | | | | | | | | | | | | |
| B. PROTOCOL B | | | | | | | | | | | | |
| Sequence | T1-weighted (MPRAGE) | | | | T2-weighted dual echo (FSE) | | | | 2D FLAIR (PROPELLER) | | | |
| Mode | 3D | | | | 2D | | | | 2D | | | |
| FOV (mm) | 256 | | | | 250 | | | | 250 | | | |
| Orientation | Sagittal | | | | Axial | | | | Axial | | | |
| TR (ms) | 2500 | | | | 3630 | | | | 9500 | | | |
| TE (ms) | 2.26 | | | | 9.6, 96 | | | | 120 | | | |
| TI (ms) | 1100 | | | | - | | | | 2400 | | | |
| Flip angle (deg) | 7 | | | | 150 | | | | 150 | | | |
| Gap (mm) | - | | | | 0 | | | | 0 | | | |
| Matrix (mm) | 256 x 256 | | | | 384 x 384 | | | | 256 x 256 | | | |
| Voxel size (mm) | 1 x 1 x1 | | | | 0.7 x 0.7 x 3 | | | | 1 x 1 x 3 | | | |
| Slices | 176 | | | | 60 | | | | 60 | | | |
| Acceleration factor (in-plane × slice) | 2 × 1 | | | | 3 × 1 | | | | 2 × 1 | | | |
| Acq. Time (m:ss) | 5:59 | | | | 4:01 | | | | 4:47 | | | |

FLAIR = fluid attenuated inversion recovery; EDI1 = Edinburgh site 1; GLA = Glasgow; DUN = Dundee; ABN = Aberdeen;
 FOV = field of view; TR = repetition time; TE = echo time; TI = inversion time; deg = degree; acq. = acquisition; sag = sagittal; Ax = axial
