# Supplementary material for: Patterns of brain atrophy in recently-diagnosed relapsing-remitting multiple sclerosis
Source: PLoS One. 2023 Jul 28;18(7):e0288967. doi: 10.1371/journal.pone.0288967 (PMC10381059; doi:10.1371/journal.pone.0288967)
Supplement: S2 Table — (DOCX) [file pone.0288967.s002.docx]

**S2 Table. Statistical parameters for linear mixed-effects models evaluating the effect of DMT use at w1 (yes/no) on brain tissue volume change over time.** The models were corrected for age, sex, imaging site and WML change. Standardised regression coeffients are shown. P-values shown are uncorrected for multiple comparisons and did not survive FDR correction (q<0.05).

|  | B_standardised_ | SE | t-value | P_uncorrected_ | CI 2.5. | CI 97.5. |
| --- | --- | --- | --- | --- | --- | --- |
| Accumbens L | -0.00000014 | 0.00000341 | -0.03976 | 0.9683 | -0.00000685 | 0.00000658 |
| Accumbens R | 0.00000044 | 0.00000333 | 0.13254 | 0.8946 | -0.00000611 | 0.00000699 |
| Amygdala L | -0.00000833 | 0.00000623 | -1.33795 | 0.1819 | -0.00002059 | 0.00000392 |
| Amygdala R | -0.00000985 | 0.00000588 | -1.67564 | 0.0949 | -0.00002142 | 0.00000172 |
| Basal Ganglia L | 0.00001171 | 0.00002511 | 0.46652 | 0.6412 | -0.00003770 | 0.00006113 |
| Basal Ganglia R | -0.00000368 | 0.00002671 | -0.13764 | 0.8906 | -0.00005625 | 0.00004889 |
| Brainstem | -0.00003264 | 0.00003390 | -0.96278 | 0.3365 | -0.00009936 | 0.00003408 |
| GM cerebellar L | -0.00006125 | 0.00009202 | -0.66566 | 0.5061 | -0.00024236 | 0.00011985 |
| GM cerebellar R | -0.00000949 | 0.00010189 | -0.09318 | 0.9258 | -0.00021003 | 0.00019104 |
| GM cortical L | -0.00040273 | 0.00038349 | -1.05016 | 0.2945 | -0.00115745 | 0.00035199 |
| GM cortical R | -0.00067305 | 0.00039157 | -1.71884 | 0.0867 | -0.00144367 | 0.00009757 |
| Hippocampus L | -0.00002002 | 0.00000955 | -2.09687 | 0.0369 | -0.00003881 | -0.00000123 |
| Hippocampus R | -0.00000750 | 0.00000810 | -0.92507 | 0.3557 | -0.00002344 | 0.00000845 |
| NAWM cerebellar L | -0.00001046 | 0.00004410 | -0.23721 | 0.8127 | -0.00009725 | 0.00007633 |
| NAWM cerebellar R | 0.00004638 | 0.00004147 | 1.11820 | 0.2644 | -0.00003524 | 0.00012799 |
| NAWM cerebral L | -0.00013351 | 0.00027528 | -0.48500 | 0.6280 | -0.00067529 | 0.00040827 |
| NAWM cerebral R | -0.00032252 | 0.00029226 | -1.10355 | 0.2707 | -0.00089771 | 0.00025267 |
| Thalamus L | 0.00000427 | 0.00001279 | 0.33355 | 0.7390 | -0.00002091 | 0.00002945 |
| Thalamus R | -0.00000535 | 0.00001038 | -0.51549 | 0.6066 | -0.00002577 | 0.00001507 |
| Ventral DC L | 0.00000027 | 0.00000867 | 0.03104 | 0.9753 | -0.00001680 | 0.00001734 |
| Ventral DC R | 0.00000091 | 0.00000973 | 0.09366 | 0.9254 | -0.00001824 | 0.00002006 |
| Whole-brain | -0.00189780 | 0.00117214 | -1.61909 | 0.1065 | -0.00420468 | 0.00040908 |

B=standardised beta value, SE=standard error, CI=confidence interval for beta value, w0=baseline, w1=1-year follow-up, L=left, R=right, GM=grey matter, NAWM=normal-appearing white matter, WML=white matter lesion, DC=diencephalon, FDR=false discovery rate.
