# Supplementary material for: Patterns of brain atrophy in recently-diagnosed relapsing-remitting multiple sclerosis
Source: PLoS One. 2023 Jul 28;18(7):e0288967. doi: 10.1371/journal.pone.0288967 (PMC10381059; doi:10.1371/journal.pone.0288967)
Supplement: S3 Table — (DOCX) [file pone.0288967.s003.docx]

**S3 Table**. **Detailed statistical parameters for linear mixed-effect models evaluating the effect of time on brain volumes**. The models were corrected for age, sex, imaging site, DMT status at w1 and WML change. Left cerebellar GM and bilateral superiorfrontal NAWM also included the significant time*age interaction. Standardised regression coeffients are shown. P-values shown are uncorrected for multiple comparisons.

|  | Coefficient | B_standardised_ | SE | t-value | P_uncorrected_ | CI 2.5. | CI 97.5. |
| --- | --- | --- | --- | --- | --- | --- | --- |
| Accumbens L | Intercept | -0.3445 | 0.2444 | -1.4094 | 0.1597 | -0.8181 | 0.1291 |
|  | Time | -0.0546 | 0.0210 | -2.6078 | **0.0096** | -0.0958 | -0.0135 |
|  | WML change | -0.0517 | 0.0530 | -0.9762 | 0.3297 | -0.1543 | 0.0509 |
|  | Age | -0.3293 | 0.0532 | -6.1904 | **<0.0001** | -0.4323 | -0.2262 |
|  | Sex: Male | -0.2628 | 0.1205 | -2.1806 | **0.0300** | -0.4963 | -0.0293 |
|  | Site: ED2 | 0.2551 | 0.2638 | 0.9672 | 0.3342 | -0.2560 | 0.7663 |
|  | Site: ED1 | 0.5282 | 0.2644 | 1.9977 | **0.0466** | 0.0159 | 1.0405 |
|  | Site: DUN | 0.4888 | 0.2926 | 1.6704 | 0.0959 | -0.0782 | 1.0557 |
|  | Site: GLA | 0.4522 | 0.2622 | 1.7247 | 0.0856 | -0.0558 | 0.9602 |
|  | DMT | 0.0389 | 0.1266 | 0.3069 | 0.7591 | -0.2065 | 0.2842 |
| Accumbens R | Intercept | 0.1057 | 0.2323 | 0.4551 | 0.6494 | -0.3439 | 0.5554 |
|  | Time | -0.0787 | 0.0229 | -3.4413 | **0.0007** | -0.1236 | -0.0338 |
|  | WML change | -0.0625 | 0.0525 | -1.1897 | 0.2352 | -0.1642 | 0.0392 |
|  | Age | -0.3432 | 0.0525 | -6.5364 | **<0.0001** | -0.4449 | -0.2416 |
|  | Sex: Male | -0.5853 | 0.1197 | -4.8885 | **<0.0001** | -0.8170 | -0.3535 |
|  | Site: ED2 | -0.2304 | 0.2512 | -0.9172 | 0.3598 | -0.7166 | 0.2558 |
|  | Site: ED1 | 0.3450 | 0.2520 | 1.3692 | 0.1720 | -0.1427 | 0.8328 |
|  | Site: DUN | 0.1292 | 0.2851 | 0.4533 | 0.6507 | -0.4226 | 0.6811 |
|  | Site: GLA | 0.1135 | 0.2499 | 0.4541 | 0.6501 | -0.3702 | 0.5971 |
|  | DMT | -0.0118 | 0.1231 | -0.0958 | 0.9237 | -0.2501 | 0.2265 |
| Amygdala L | Intercept | 0.3682 | 0.2485 | 1.4818 | 0.1394 | -0.1133 | 0.8497 |
|  | Time | -0.0963 | 0.0222 | -4.3368 | **<0.0001** | -0.1398 | -0.0527 |
|  | WML change | 0.0439 | 0.0539 | 0.8140 | 0.4163 | -0.0606 | 0.1483 |
|  | Age | -0.2666 | 0.0541 | -4.9288 | **<0.0001** | -0.3714 | -0.1618 |
|  | Sex: Male | -0.2449 | 0.1225 | -1.9983 | **0.0466** | -0.4823 | -0.0074 |
|  | Site: ED2 | -0.6111 | 0.2687 | -2.2743 | **0.0236** | -1.1317 | -0.0905 |
|  | Site: ED1 | -0.0238 | 0.2688 | -0.0887 | 0.9294 | -0.5446 | 0.4969 |
|  | Site: DUN | -0.3384 | 0.2974 | -1.1378 | 0.2561 | -0.9147 | 0.2379 |
|  | Site: GLA | -0.2296 | 0.2666 | -0.8614 | 0.3897 | -0.7461 | 0.2869 |
|  | DMT | 0.0107 | 0.1290 | 0.0831 | 0.9338 | -0.2393 | 0.2607 |
| Amygdala R | Intercept | 0.3646 | 0.2491 | 1.4637 | 0.1443 | -0.1180 | 0.8472 |
|  | Time | -0.1458 | 0.0217 | -6.7159 | **<0.0001** | -0.1885 | -0.1032 |
|  | WML change | 0.1463 | 0.0540 | 2.7104 | **0.0071** | 0.0417 | 0.2508 |
|  | Age | -0.2568 | 0.0542 | -4.7372 | **<0.0001** | -0.3618 | -0.1517 |
|  | Sex: Male | 0.0082 | 0.1228 | 0.0664 | 0.9471 | -0.2298 | 0.2461 |
|  | Site: ED2 | -0.5407 | 0.2688 | -2.0113 | **0.0452** | -1.0616 | -0.0198 |
|  | Site: ED1 | -0.0206 | 0.2694 | -0.0765 | 0.9390 | -0.5427 | 0.5014 |
|  | Site: DUN | -0.4885 | 0.2982 | -1.6381 | 0.1024 | -1.0662 | 0.0893 |
|  | Site: GLA | -0.2601 | 0.2672 | -0.9736 | 0.3310 | -0.7778 | 0.2576 |
|  | DMT | -0.0183 | 0.1290 | -0.1422 | 0.8870 | -0.2684 | 0.2317 |
| Basal Ganglia L | Intercept | 0.1367 | 0.2425 | 0.5637 | 0.5734 | -0.3331 | 0.6065 |
|  | Time | -0.1510 | 0.0192 | -7.8818 | **<0.0001** | -0.1887 | -0.1134 |
|  | WML change | 0.0025 | 0.0525 | 0.0473 | 0.9623 | -0.0993 | 0.1043 |
|  | Age | -0.2658 | 0.0528 | -5.0377 | **<0.0001** | -0.3681 | -0.1636 |
|  | Sex: Male | -0.4928 | 0.1196 | -4.1221 | **<0.0001** | -0.7245 | -0.2612 |
|  | Site: ED2 | 0.3734 | 0.2617 | 1.4267 | 0.1547 | -0.1337 | 0.8806 |
|  | Site: ED1 | -0.0284 | 0.2623 | -0.1082 | 0.9139 | -0.5367 | 0.4799 |
|  | Site: DUN | -0.2713 | 0.2903 | -0.9344 | 0.3508 | -0.8338 | 0.2912 |
|  | Site: GLA | -0.1448 | 0.2601 | -0.5567 | 0.5782 | -0.6488 | 0.3592 |
|  | DMT | 0.0911 | 0.1256 | 0.7253 | 0.4688 | -0.1523 | 0.3346 |
| Basal Ganglia R | Intercept | 0.2778 | 0.2382 | 1.1664 | 0.2444 | -0.1837 | 0.7393 |
|  | Time | -0.1646 | 0.0200 | -8.2182 | **<0.0001** | -0.2039 | -0.1253 |
|  | WML change | 0.0180 | 0.0516 | 0.3488 | 0.7275 | -0.0820 | 0.1180 |
|  | Age | -0.2578 | 0.0518 | -4.9744 | **<0.0001** | -0.3583 | -0.1574 |
|  | Sex: Male | -0.5353 | 0.1174 | -4.5580 | **<0.0001** | -0.7628 | -0.3077 |
|  | Site: ED2 | 0.2985 | 0.2571 | 1.1613 | 0.2464 | -0.1996 | 0.7967 |
|  | Site: ED1 | -0.3117 | 0.2577 | -1.2096 | 0.2274 | -0.8109 | 0.1876 |
|  | Site: DUN | -0.3974 | 0.2852 | -1.3938 | 0.1644 | -0.9499 | 0.1551 |
|  | Site: GLA | -0.3288 | 0.2555 | -1.2871 | 0.1990 | -0.8239 | 0.1662 |
|  | DMT | 0.1501 | 0.1234 | 1.2165 | 0.2247 | -0.0890 | 0.3892 |
| Brainstem | Intercept | 0.0792 | 0.2535 | 0.3125 | 0.7549 | -0.4119 | 0.5703 |
|  | Time | -0.0671 | 0.0136 | -4.9164 | **<0.0001** | -0.0938 | -0.0403 |
|  | WML change | -0.0187 | 0.0550 | -0.3407 | 0.7336 | -0.1253 | 0.0878 |
|  | Age | -0.1264 | 0.0553 | -2.2883 | **0.0228** | -0.2335 | -0.0194 |
|  | Sex: Male | -0.1489 | 0.1251 | -1.1905 | 0.2348 | -0.3913 | 0.0934 |
|  | Site: ED2 | 0.4579 | 0.2736 | 1.6737 | 0.0952 | -0.0722 | 0.9881 |
|  | Site: ED1 | -0.2997 | 0.2742 | -1.0928 | 0.2754 | -0.8310 | 0.2317 |
|  | Site: DUN | -0.2139 | 0.3035 | -0.7048 | 0.4815 | -0.8019 | 0.3741 |
|  | Site: GLA | -0.2969 | 0.2723 | -1.0904 | 0.2764 | -0.8245 | 0.2307 |
|  | DMT | 0.1274 | 0.1324 | 0.9624 | 0.3366 | -0.1291 | 0.3840 |
| GM cerebellar L | Intercept | 0.0017 | 0.2404 | 0.0070 | 0.9944 | -0.4640 | 0.4674 |
|  | Time*Age | 0.0345 | 0.0115 | 2.9944 | **0.0030** | 0.0119 | 0.0571 |
|  | Time | -0.0520 | 0.0115 | -4.5135 | **<0.0001** | -0.0745 | -0.0294 |
|  | WML change | 0.0440 | 0.0522 | 0.8430 | 0.3999 | -0.0571 | 0.1451 |
|  | Age | -0.3834 | 0.0527 | -7.2700 | **<0.0001** | -0.4856 | -0.2812 |
|  | Sex: Male | -0.3422 | 0.1186 | -2.8850 | **0.0042** | -0.5720 | -0.1124 |
|  | Site: ED2 | 0.2717 | 0.2600 | 1.0451 | 0.2968 | -0.2320 | 0.7754 |
|  | Site: ED1 | -0.3451 | 0.2601 | -1.3266 | 0.1857 | -0.8490 | 0.1589 |
|  | Site: DUN | 0.0508 | 0.2879 | 0.1763 | 0.8601 | -0.5070 | 0.6086 |
|  | Site: GLA | 0.0543 | 0.2580 | 0.2104 | 0.8335 | -0.4455 | 0.5541 |
|  | DMT | 0.1378 | 0.1251 | 1.1017 | 0.2714 | -0.1045 | 0.3802 |
| GM cerebellar R | Intercept | -0.2554 | 0.2452 | -1.0416 | 0.2984 | -0.7306 | 0.2197 |
|  | Time | -0.0615 | 0.0127 | -4.8253 | **<0.0001** | -0.0865 | -0.0365 |
|  | WML change | 0.0277 | 0.0532 | 0.5212 | 0.6026 | -0.0753 | 0.1307 |
|  | Age | -0.3283 | 0.0534 | -6.1490 | **<0.0001** | -0.4318 | -0.2249 |
|  | Sex: Male | -0.2193 | 0.1210 | -1.8128 | 0.0708 | -0.4537 | 0.0151 |
|  | Site: ED2 | 0.5513 | 0.2648 | 2.0818 | **0.0382** | 0.0382 | 1.0644 |
|  | Site: ED1 | -0.0575 | 0.2654 | -0.2168 | 0.8285 | -0.5718 | 0.4567 |
|  | Site: DUN | 0.2951 | 0.2937 | 1.0048 | 0.3158 | -0.2740 | 0.8643 |
|  | Site: GLA | 0.3311 | 0.2632 | 1.2580 | 0.2093 | -0.1789 | 0.8410 |
|  | DMT | 0.0939 | 0.1271 | 0.7391 | 0.4604 | -0.1523 | 0.3402 |
| GM cerebral L | Intercept | -0.2859 | 0.2041 | -1.4010 | 0.1622 | -0.6814 | 0.1095 |
|  | Time | -0.0861 | 0.0165 | -5.2088 | **<0.0001** | -0.1186 | -0.0537 |
|  | WML change | 0.0271 | 0.0442 | 0.6125 | 0.5407 | -0.0586 | 0.1128 |
|  | Age | -0.5175 | 0.0444 | -11.6519 | **<0.0001** | -0.6036 | -0.4315 |
|  | Sex: Male | -0.3755 | 0.1006 | -3.7315 | **0.0002** | -0.5705 | -0.1805 |
|  | Site: ED2 | 1.0005 | 0.2203 | 4.5418 | **<0.0001** | 0.5737 | 1.4273 |
|  | Site: ED1 | 0.0796 | 0.2208 | 0.3607 | 0.7186 | -0.3482 | 0.5074 |
|  | Site: DUN | 0.2820 | 0.2443 | 1.1540 | 0.2494 | -0.1915 | 0.7554 |
|  | Site: GLA | 0.5574 | 0.2189 | 2.5458 | **0.0114** | 0.1332 | 0.9816 |
|  | DMT | -0.0938 | 0.1057 | -0.8869 | 0.3759 | -0.2987 | 0.1111 |
| GM cerebral R | Intercept | -0.3185 | 0.2018 | -1.5784 | 0.1155 | -0.7095 | 0.0725 |
|  | Time | -0.0608 | 0.0169 | -3.5949 | **0.0004** | -0.0941 | -0.0276 |
|  | WML change | 0.0188 | 0.0437 | 0.4310 | 0.6668 | -0.0659 | 0.1036 |
|  | Age | -0.5250 | 0.0439 | -11.9547 | **<0.0001** | -0.6100 | -0.4399 |
|  | Sex: Male | -0.3598 | 0.0995 | -3.6161 | **0.0003** | -0.5525 | -0.1670 |
|  | Site: ED2 | 1.0756 | 0.2178 | 4.9386 | **<0.0001** | 0.6536 | 1.4976 |
|  | Site: ED1 | 0.1311 | 0.2183 | 0.6006 | 0.5486 | -0.2919 | 0.5541 |
|  | Site: DUN | 0.3288 | 0.2416 | 1.3609 | 0.1746 | -0.1393 | 0.7968 |
|  | Site: GLA | 0.5606 | 0.2165 | 2.5898 | **0.0101** | 0.1412 | 0.9800 |
|  | DMT | -0.1198 | 0.1045 | -1.1461 | 0.2526 | -0.3224 | 0.0827 |
| Hippocampus L | Intercept | 0.7295 | 0.2490 | 2.9294 | **0.0037** | 0.2470 | 1.2121 |
|  | Time | -0.1005 | 0.0177 | -5.6859 | **<0.0001** | -0.1352 | -0.0658 |
|  | WML change | 0.0064 | 0.0540 | 0.1184 | 0.9058 | -0.0982 | 0.1110 |
|  | Age | -0.2438 | 0.0542 | -4.4970 | **<0.0001** | -0.3488 | -0.1387 |
|  | Sex: Male | -0.5224 | 0.1228 | -4.2537 | **<0.0001** | -0.7604 | -0.2845 |
|  | Site: ED2 | -0.5524 | 0.2689 | -2.0548 | **0.0408** | -1.0734 | -0.0315 |
|  | Site: ED1 | -0.7854 | 0.2695 | -2.9146 | **0.0038** | -1.3075 | -0.2633 |
|  | Site: DUN | -0.8280 | 0.2982 | -2.7766 | **0.0058** | -1.4059 | -0.2502 |
|  | Site: GLA | -0.5743 | 0.2672 | -2.1494 | **0.0324** | -1.0921 | -0.0566 |
|  | DMT | 0.0915 | 0.1291 | 0.7089 | 0.4789 | -0.1586 | 0.3415 |
| Hippocampus R | Intercept | 0.6501 | 0.2475 | 2.6269 | **0.0090** | 0.1706 | 1.1296 |
|  | Time | -0.0951 | 0.0163 | -5.8384 | **<0.0001** | -0.1271 | -0.0632 |
|  | WML change | 0.0267 | 0.0536 | 0.4976 | 0.6191 | -0.0772 | 0.1306 |
|  | Age | -0.2292 | 0.0539 | -4.2554 | **<0.0001** | -0.3336 | -0.1249 |
|  | Sex: Male | -0.6700 | 0.1221 | -5.4897 | **<0.0001** | -0.9065 | -0.4336 |
|  | Site: ED2 | -0.5170 | 0.2672 | -1.9349 | 0.0539 | -1.0347 | 0.0007 |
|  | Site: ED1 | -0.6184 | 0.2678 | -2.3093 | **0.0216** | -1.1373 | -0.0995 |
|  | Site: DUN | -0.6597 | 0.2964 | -2.2259 | **0.0267** | -1.2339 | -0.0855 |
|  | Site: GLA | -0.4980 | 0.2655 | -1.8753 | 0.0617 | -1.0125 | 0.0165 |
|  | DMT | 0.1166 | 0.1283 | 0.9092 | 0.3639 | -0.1319 | 0.3651 |
| NAWM cerebellar L | Intercept | 0.4473 | 0.2519 | 1.7756 | 0.0768 | -0.0408 | 0.9355 |
|  | Time | -0.0215 | 0.0168 | -1.2787 | 0.2019 | -0.0546 | 0.0115 |
|  | WML change | -0.0410 | 0.0546 | -0.7502 | 0.4537 | -0.1468 | 0.0648 |
|  | Age | -0.0219 | 0.0548 | -0.3985 | 0.6905 | -0.1281 | 0.0844 |
|  | Sex: Male | -0.5098 | 0.1243 | -4.1029 | **0.0001** | -0.7506 | -0.2690 |
|  | Site: ED2 | -0.4429 | 0.2720 | -1.6284 | 0.1045 | -0.9699 | 0.0841 |
|  | Site: ED1 | -0.0473 | 0.2726 | -0.1735 | 0.8624 | -0.5755 | 0.4809 |
|  | Site: DUN | 0.0985 | 0.3017 | 0.3265 | 0.7443 | -0.4861 | 0.6831 |
|  | Site: GLA | -0.5763 | 0.2703 | -2.1317 | **0.0338** | -1.1001 | -0.0525 |
|  | DMT | 0.0149 | 0.1306 | 0.1144 | 0.9090 | -0.2380 | 0.2679 |
| NAWM cerebellar R | Intercept | 0.6023 | 0.2533 | 2.3775 | **0.0181** | 0.1115 | 1.0930 |
|  | Time | 0.0096 | 0.0162 | 0.5956 | 0.5519 | -0.0221 | 0.0414 |
|  | WML change | 0.0067 | 0.0551 | 0.1209 | 0.9039 | -0.1001 | 0.1134 |
|  | Age | -0.0920 | 0.0553 | -1.6617 | 0.0976 | -0.1992 | 0.0153 |
|  | Sex: Male | -0.4532 | 0.1250 | -3.6245 | **0.0003** | -0.6954 | -0.2109 |
|  | Site: ED2 | -0.7272 | 0.2735 | -2.6587 | **0.0083** | -1.2572 | -0.1973 |
|  | Site: ED1 | -0.2876 | 0.2745 | -1.0475 | 0.2957 | -0.8194 | 0.2443 |
|  | Site: DUN | -0.0783 | 0.3034 | -0.2579 | 0.7966 | -0.6662 | 0.5096 |
|  | Site: GLA | -0.7109 | 0.2719 | -2.6146 | **0.0094** | -1.2377 | -0.1841 |
|  | DMT | 0.0271 | 0.1316 | 0.2061 | 0.8368 | -0.2279 | 0.2821 |
| NAWM cerebral L | Intercept | 0.3732 | 0.2604 | 1.4331 | 0.1528 | -0.1313 | 0.8778 |
|  | Time | -0.1378 | 0.0121 | -11.3639 | **<0.0001** | -0.1617 | -0.1140 |
|  | WML change | -0.1246 | 0.0568 | -2.1925 | **0.0291** | -0.2347 | -0.0145 |
|  | Age | -0.0129 | 0.0572 | -0.2248 | 0.8223 | -0.1237 | 0.0980 |
|  | Sex: Male | -0.0862 | 0.1289 | -0.6693 | 0.5038 | -0.3359 | 0.1634 |
|  | Site: ED2 | 0.0377 | 0.2813 | 0.1340 | 0.8935 | -0.5074 | 0.5828 |
|  | Site: ED1 | -0.5868 | 0.2822 | -2.0796 | **0.0384** | -1.1335 | -0.0401 |
|  | Site: DUN | -0.3560 | 0.3123 | -1.1398 | 0.2553 | -0.9610 | 0.2491 |
|  | Site: GLA | -0.4103 | 0.2800 | -1.4650 | 0.1439 | -0.9528 | 0.1323 |
|  | DMT | 0.0549 | 0.1352 | 0.4062 | 0.6849 | -0.2070 | 0.3168 |
| NAWM cerebral R | Intercept | 0.2486 | 0.2603 | 0.9548 | 0.3404 | -0.2559 | 0.7530 |
|  | Time | -0.1263 | 0.0120 | -10.5151 | **<0.0001** | -0.1498 | -0.1027 |
|  | WML change | -0.0247 | 0.0564 | -0.4373 | 0.6622 | -0.1340 | 0.0847 |
|  | Age | -0.0300 | 0.0567 | -0.5294 | 0.5969 | -0.1399 | 0.0798 |
|  | Sex: Male | -0.1086 | 0.1284 | -0.8458 | 0.3983 | -0.3575 | 0.1402 |
|  | Site: ED2 | 0.2326 | 0.2812 | 0.8274 | 0.4086 | -0.3121 | 0.7774 |
|  | Site: ED1 | -0.4809 | 0.2818 | -1.7065 | 0.0889 | -1.0269 | 0.0651 |
|  | Site: DUN | -0.3087 | 0.3119 | -0.9898 | 0.3231 | -0.9130 | 0.2956 |
|  | Site: GLA | -0.2658 | 0.2794 | -0.9513 | 0.3422 | -0.8073 | 0.2756 |
|  | DMT | 0.0493 | 0.1350 | 0.3650 | 0.7154 | -0.2122 | 0.3108 |
| Thalamus L | Intercept | 0.7492 | 0.2446 | 3.0624 | **0.0024** | 0.2752 | 1.2232 |
|  | Time | -0.1163 | 0.0116 | -10.0684 | **<0.0001** | -0.1390 | -0.0937 |
|  | WML change | -0.0878 | 0.0530 | -1.6564 | 0.0987 | -0.1906 | 0.0149 |
|  | Age | -0.2964 | 0.0533 | -5.5637 | **<0.0001** | -0.3996 | -0.1932 |
|  | Sex: Male | -0.5713 | 0.1207 | -4.7339 | **<0.0001** | -0.8052 | -0.3375 |
|  | Site: ED2 | -0.4616 | 0.2642 | -1.7470 | 0.0816 | -0.9735 | 0.0504 |
|  | Site: ED1 | -0.7277 | 0.2648 | -2.7479 | **0.0064** | -1.2408 | -0.2146 |
|  | Site: DUN | -0.7353 | 0.2931 | -2.5092 | **0.0126** | -1.3032 | -0.1675 |
|  | Site: GLA | -0.6180 | 0.2626 | -2.3534 | **0.0192** | -1.1267 | -0.1092 |
|  | DMT | 0.0549 | 0.1268 | 0.4325 | 0.6657 | -0.1909 | 0.3006 |
| Thalamus R | Intercept | 0.3886 | 0.2395 | 1.6226 | 0.1057 | -0.0754 | 0.8527 |
|  | Time | -0.1055 | 0.0107 | -9.8826 | **<0.0001** | -0.1265 | -0.0846 |
|  | WML change | -0.1786 | 0.0521 | -3.4269 | **0.0007** | -0.2796 | -0.0776 |
|  | Age | -0.3021 | 0.0525 | -5.7517 | **<0.0001** | -0.4039 | -0.2003 |
|  | Sex: Male | -0.5928 | 0.1185 | -5.0023 | **<0.0001** | -0.8224 | -0.3632 |
|  | Site: ED2 | -0.1476 | 0.2587 | -0.5706 | 0.5687 | -0.6489 | 0.3537 |
|  | Site: ED1 | -0.2595 | 0.2595 | -0.9998 | 0.3182 | -0.7623 | 0.2433 |
|  | Site: DUN | -0.2395 | 0.2872 | -0.8338 | 0.4050 | -0.7960 | 0.3170 |
|  | Site: GLA | -0.3783 | 0.2575 | -1.4687 | 0.1429 | -0.8772 | 0.1207 |
|  | DMT | 0.1009 | 0.1244 | 0.8108 | 0.4181 | -0.1402 | 0.3419 |
| Ventral DC L | Intercept | 0.1537 | 0.2485 | 0.6184 | 0.5368 | -0.3278 | 0.6352 |
|  | Time | -0.0763 | 0.0173 | -4.4036 | **<0.0001** | -0.1103 | -0.0423 |
|  | WML change | -0.0870 | 0.0540 | -1.6101 | 0.1084 | -0.1916 | 0.0177 |
|  | Age | -0.2738 | 0.0542 | -5.0515 | **<0.0001** | -0.3789 | -0.1688 |
|  | Sex: Male | -0.2608 | 0.1226 | -2.1277 | **0.0342** | -0.4983 | -0.0233 |
|  | Site: ED2 | -0.0533 | 0.2686 | -0.1985 | 0.8428 | -0.5737 | 0.4670 |
|  | Site: ED1 | 0.0110 | 0.2688 | 0.0409 | 0.9674 | -0.5097 | 0.5317 |
|  | Site: DUN | -0.2667 | 0.2975 | -0.8966 | 0.3707 | -0.8431 | 0.3096 |
|  | Site: GLA | -0.4320 | 0.2667 | -1.6197 | 0.1063 | -0.9488 | 0.0847 |
|  | DMT | 0.2082 | 0.1294 | 1.6090 | 0.1087 | -0.0425 | 0.4588 |
| Ventral DC R | Intercept | 0.2995 | 0.2368 | 1.2647 | 0.2070 | -0.1589 | 0.7580 |
|  | Time | -0.1306 | 0.0186 | -7.0225 | **<0.0001** | -0.1671 | -0.0941 |
|  | WML change | -0.0354 | 0.0535 | -0.6615 | 0.5088 | -0.1390 | 0.0682 |
|  | Age | -0.3876 | 0.0535 | -7.2440 | **<0.0001** | -0.4911 | -0.2840 |
|  | Sex: Male | -0.3170 | 0.1221 | -2.5967 | **0.0099** | -0.5533 | -0.0807 |
|  | Site: ED2 | -0.4216 | 0.2557 | -1.6487 | 0.1003 | -0.9166 | 0.0734 |
|  | Site: ED1 | -0.0941 | 0.2571 | -0.3660 | 0.7147 | -0.5917 | 0.4035 |
|  | Site: DUN | -0.1538 | 0.2908 | -0.5291 | 0.5972 | -0.7167 | 0.4090 |
|  | Site: GLA | -0.4696 | 0.2548 | -1.8427 | 0.0664 | -0.9628 | 0.0237 |
|  | DMT | 0.2174 | 0.1252 | 1.7371 | 0.0835 | -0.0249 | 0.4597 |
| Whole-brain | Intercept | -0.0045 | 0.2250 | -0.0200 | 0.9840 | -0.4404 | 0.4314 |
|  | Time | -0.1001 | 0.0146 | -6.8463 | **<0.0001** | -0.1288 | -0.0714 |
|  | WML change | 0.0096 | 0.0488 | 0.1976 | 0.8435 | -0.0848 | 0.1041 |
|  | Age | -0.3700 | 0.0490 | -7.5563 | **<0.0001** | -0.4648 | -0.2751 |
|  | Sex: Male | -0.3840 | 0.1104 | -3.4779 | **0.0006** | -0.5979 | -0.1701 |
|  | Site: ED2 | 0.7604 | 0.2430 | 3.1289 | **0.0019** | 0.2895 | 1.2313 |
|  | Site: ED1 | -0.2759 | 0.2437 | -1.1319 | 0.2585 | -0.7481 | 0.1964 |
|  | Site: DUN | 0.0508 | 0.2696 | 0.1882 | 0.8508 | -0.4717 | 0.5732 |
|  | Site: GLA | 0.1289 | 0.2416 | 0.5334 | 0.5941 | -0.3393 | 0.5970 |
|  | DMT | -0.0176 | 0.1169 | -0.1507 | 0.8803 | -0.2442 | 0.2089 |
| GM caudal anterior cingulate L | Intercept | -0.4342 | 0.2637 | -1.6465 | 0.1007 | -0.9452 | 0.0768 |
|  | Time | -0.0441 | 0.0078 | -5.6479 | **<0.0001** | -0.0595 | -0.0288 |
|  | WML change | 0.0436 | 0.0573 | 0.7608 | 0.4473 | -0.0674 | 0.1546 |
|  | Age | -0.0785 | 0.0575 | -1.3642 | 0.1735 | -0.1900 | 0.0330 |
|  | Sex: Male | -0.2068 | 0.1301 | -1.5889 | 0.1131 | -0.4589 | 0.0454 |
|  | Site: ED2 | 0.7727 | 0.2852 | 2.7095 | **0.0071** | 0.2201 | 1.3253 |
|  | Site: ED1 | 0.3546 | 0.2857 | 1.2414 | 0.2154 | -0.1989 | 0.9081 |
|  | Site: DUN | 0.5912 | 0.3164 | 1.8684 | 0.0627 | -0.0219 | 1.2042 |
|  | Site: GLA | 0.5731 | 0.2840 | 2.0176 | **0.0445** | 0.0227 | 1.1234 |
|  | DMT | -0.0471 | 0.1379 | -0.3415 | 0.7330 | -0.3142 | 0.2200 |
| GM caudal anterior cingulate R | Intercept | 0.1152 | 0.2492 | 0.4622 | 0.6442 | -0.3677 | 0.5981 |
|  | Time | -0.0223 | 0.0073 | -3.0396 | **0.0026** | -0.0367 | -0.0079 |
|  | WML change | -0.0215 | 0.0540 | -0.3989 | 0.6903 | -0.1262 | 0.0831 |
|  | Age | -0.2256 | 0.0542 | -4.1628 | **<0.0001** | -0.3306 | -0.1206 |
|  | Sex: Male | -0.3766 | 0.1224 | -3.0781 | **0.0023** | -0.6137 | -0.1395 |
|  | Site: ED2 | 0.1444 | 0.2694 | 0.5360 | 0.5923 | -0.3776 | 0.6663 |
|  | Site: ED1 | -0.3549 | 0.2696 | -1.3164 | 0.1890 | -0.8774 | 0.1675 |
|  | Site: DUN | 0.6808 | 0.2988 | 2.2785 | **0.0234** | 0.1018 | 1.2597 |
|  | Site: GLA | -0.0392 | 0.2677 | -0.1466 | 0.8835 | -0.5580 | 0.4795 |
|  | DMT | -0.0249 | 0.1291 | -0.1930 | 0.8471 | -0.2750 | 0.2252 |
| GM caudal middle frontal L | Intercept | -0.0131 | 0.2410 | -0.0544 | 0.9566 | -0.4801 | 0.4539 |
|  | Time | -0.0325 | 0.0131 | -2.4729 | **0.0139** | -0.0582 | -0.0067 |
|  | WML change | -0.0131 | 0.0524 | -0.2501 | 0.8027 | -0.1145 | 0.0883 |
|  | Age | -0.2271 | 0.0526 | -4.3213 | **<0.0001** | -0.3290 | -0.1253 |
|  | Sex: Male | -0.3408 | 0.1197 | -2.8485 | **0.0047** | -0.5727 | -0.1090 |
|  | Site: ED2 | 0.7342 | 0.2605 | 2.8185 | **0.0051** | 0.2295 | 1.2389 |
|  | Site: ED1 | -0.2599 | 0.2608 | -0.9967 | 0.3197 | -0.7652 | 0.2454 |
|  | Site: DUN | 0.2393 | 0.2886 | 0.8290 | 0.4077 | -0.3199 | 0.7985 |
|  | Site: GLA | 0.4596 | 0.2586 | 1.7771 | 0.0766 | -0.0415 | 0.9607 |
|  | DMT | -0.2649 | 0.1250 | -2.1183 | **0.0350** | -0.5071 | -0.0226 |
| GM caudal middle frontal R | Intercept | 0.3736 | 0.2477 | 1.5083 | 0.1325 | -0.1063 | 0.8535 |
|  | Time | -0.0279 | 0.0132 | -2.1094 | **0.0357** | -0.0538 | -0.0019 |
|  | WML change | -0.0427 | 0.0538 | -0.7929 | 0.4284 | -0.1469 | 0.0616 |
|  | Age | -0.1968 | 0.0540 | -3.6440 | **0.0003** | -0.3014 | -0.0922 |
|  | Sex: Male | -0.3644 | 0.1217 | -2.9955 | **0.0030** | -0.6002 | -0.1287 |
|  | Site: ED2 | 0.1596 | 0.2679 | 0.5957 | 0.5518 | -0.3594 | 0.6785 |
|  | Site: ED1 | -0.6950 | 0.2681 | -2.5924 | **0.0100** | -1.2145 | -0.1756 |
|  | Site: DUN | -0.1982 | 0.2969 | -0.6676 | 0.5049 | -0.7735 | 0.3770 |
|  | Site: GLA | -0.0722 | 0.2661 | -0.2714 | 0.7863 | -0.5877 | 0.4433 |
|  | DMT | -0.1265 | 0.1285 | -0.9846 | 0.3256 | -0.3755 | 0.1225 |
| GM frontal pole L | Intercept | 0.3317 | 0.2413 | 1.3747 | 0.1702 | -0.1358 | 0.7993 |
|  | Time | -0.0293 | 0.0203 | -1.4411 | 0.1506 | -0.0691 | 0.0106 |
|  | WML change | 0.0438 | 0.0525 | 0.8347 | 0.4046 | -0.0579 | 0.1456 |
|  | Age | -0.3774 | 0.0527 | -7.1663 | **<0.0001** | -0.4795 | -0.2754 |
|  | Sex: Male | -0.2400 | 0.1185 | -2.0242 | **0.0438** | -0.4696 | -0.0103 |
|  | Site: ED2 | 0.1535 | 0.2607 | 0.5887 | 0.5565 | -0.3517 | 0.6587 |
|  | Site: ED1 | -0.1467 | 0.2612 | -0.5615 | 0.5749 | -0.6528 | 0.3594 |
|  | Site: DUN | -0.2534 | 0.2892 | -0.8762 | 0.3816 | -0.8136 | 0.3069 |
|  | Site: GLA | 0.0600 | 0.2592 | 0.2313 | 0.8172 | -0.4422 | 0.5621 |
|  | DMT | -0.3456 | 0.1251 | -2.7635 | **0.0061** | -0.5879 | -0.1033 |
| GM frontal pole R | Intercept | -0.0431 | 0.2430 | -0.1774 | 0.8593 | -0.5139 | 0.4277 |
|  | Time | -0.0381 | 0.0193 | -1.9739 | **0.0493** | -0.0759 | -0.0002 |
|  | WML change | 0.0467 | 0.0528 | 0.8834 | 0.3777 | -0.0557 | 0.1490 |
|  | Age | -0.3962 | 0.0531 | -7.4573 | **<0.0001** | -0.4991 | -0.2932 |
|  | Sex: Male | -0.1331 | 0.1199 | -1.1103 | 0.2677 | -0.3653 | 0.0991 |
|  | Site: ED2 | 0.2348 | 0.2625 | 0.8945 | 0.3718 | -0.2738 | 0.7435 |
|  | Site: ED1 | 0.0525 | 0.2628 | 0.1999 | 0.8417 | -0.4566 | 0.5617 |
|  | Site: DUN | 0.0074 | 0.2913 | 0.0255 | 0.9797 | -0.5570 | 0.5719 |
|  | Site: GLA | 0.2983 | 0.2615 | 1.1407 | 0.2549 | -0.2084 | 0.8051 |
|  | DMT | -0.1152 | 0.1271 | -0.9066 | 0.3653 | -0.3615 | 0.1310 |
| GM lateral orbito frontal L | Intercept | -0.4547 | 0.2416 | -1.8825 | 0.0607 | -0.9227 | 0.0133 |
|  | Time | -0.0684 | 0.0167 | -4.1102 | **0.0001** | -0.1011 | -0.0358 |
|  | WML change | 0.0047 | 0.0523 | 0.0895 | 0.9288 | -0.0967 | 0.1061 |
|  | Age | -0.3814 | 0.0526 | -7.2534 | **<0.0001** | -0.4833 | -0.2795 |
|  | Sex: Male | -0.1502 | 0.1191 | -1.2606 | 0.2084 | -0.3810 | 0.0806 |
|  | Site: ED2 | 0.7076 | 0.2608 | 2.7133 | **0.0070** | 0.2023 | 1.2129 |
|  | Site: ED1 | 0.4072 | 0.2614 | 1.5581 | 0.1203 | -0.0992 | 0.9137 |
|  | Site: DUN | 0.3843 | 0.2893 | 1.3285 | 0.1850 | -0.1762 | 0.9448 |
|  | Site: GLA | 0.5463 | 0.2592 | 2.1076 | **0.0359** | 0.0441 | 1.0484 |
|  | DMT | 0.0258 | 0.1252 | 0.2061 | 0.8369 | -0.2168 | 0.2683 |
| GM lateral orbito frontal R | Intercept | -0.2317 | 0.2393 | -0.9684 | 0.3336 | -0.6954 | 0.2319 |
|  | Time | -0.0291 | 0.0197 | -1.4790 | 0.1402 | -0.0678 | 0.0095 |
|  | WML change | -0.0603 | 0.0520 | -1.1603 | 0.2468 | -0.1611 | 0.0404 |
|  | Age | -0.3412 | 0.0523 | -6.5251 | **<0.0001** | -0.4425 | -0.2399 |
|  | Sex: Male | -0.1922 | 0.1177 | -1.6329 | 0.1035 | -0.4203 | 0.0359 |
|  | Site: ED2 | 0.5501 | 0.2585 | 2.1277 | **0.0342** | 0.0492 | 1.0510 |
|  | Site: ED1 | -0.0472 | 0.2588 | -0.1825 | 0.8553 | -0.5486 | 0.4542 |
|  | Site: DUN | 0.0893 | 0.2870 | 0.3112 | 0.7559 | -0.4668 | 0.6454 |
|  | Site: GLA | 0.2037 | 0.2573 | 0.7914 | 0.4293 | -0.2950 | 0.7023 |
|  | DMT | 0.1255 | 0.1239 | 1.0123 | 0.3122 | -0.1147 | 0.3656 |
| GM medial orbito frontal L | Intercept | 0.1676 | 0.2484 | 0.6750 | 0.5002 | -0.3135 | 0.6488 |
|  | Time | -0.0588 | 0.0220 | -2.6696 | **0.0080** | -0.1021 | -0.0156 |
|  | WML change | -0.0134 | 0.0539 | -0.2492 | 0.8033 | -0.1179 | 0.0910 |
|  | Age | -0.2873 | 0.0542 | -5.2995 | **<0.0001** | -0.3923 | -0.1822 |
|  | Sex: Male | -0.0957 | 0.1220 | -0.7842 | 0.4335 | -0.3321 | 0.1407 |
|  | Site: ED2 | 0.1626 | 0.2687 | 0.6053 | 0.5455 | -0.3579 | 0.6832 |
|  | Site: ED1 | -0.0765 | 0.2686 | -0.2849 | 0.7759 | -0.5969 | 0.4439 |
|  | Site: DUN | -0.4078 | 0.2985 | -1.3661 | 0.1729 | -0.9861 | 0.1705 |
|  | Site: GLA | 0.1820 | 0.2666 | 0.6827 | 0.4953 | -0.3346 | 0.6986 |
|  | DMT | -0.2175 | 0.1291 | -1.6845 | 0.0931 | -0.4677 | 0.0327 |
| GM medial orbito frontal R | Intercept | -0.1171 | 0.2367 | -0.4946 | 0.6213 | -0.5757 | 0.3416 |
|  | Time | -0.0137 | 0.0214 | -0.6390 | 0.5233 | -0.0556 | 0.0283 |
|  | WML change | 0.0153 | 0.0514 | 0.2971 | 0.7666 | -0.0843 | 0.1149 |
|  | Age | -0.3992 | 0.0516 | -7.7411 | **<0.0001** | -0.4991 | -0.2993 |
|  | Sex: Male | -0.1530 | 0.1167 | -1.3113 | 0.1907 | -0.3792 | 0.0731 |
|  | Site: ED2 | 0.1874 | 0.2556 | 0.7332 | 0.4640 | -0.3078 | 0.6826 |
|  | Site: ED1 | 0.0882 | 0.2558 | 0.3446 | 0.7306 | -0.4075 | 0.5838 |
|  | Site: DUN | -0.3509 | 0.2835 | -1.2379 | 0.2167 | -0.9002 | 0.1983 |
|  | Site: GLA | 0.0843 | 0.2542 | 0.3317 | 0.7403 | -0.4082 | 0.5768 |
|  | DMT | 0.1419 | 0.1225 | 1.1584 | 0.2476 | -0.0954 | 0.3792 |
| GM paracentral L | Intercept | -0.3193 | 0.2458 | -1.2989 | 0.1949 | -0.7956 | 0.1570 |
|  | Time | -0.0157 | 0.0170 | -0.9226 | 0.3569 | -0.0490 | 0.0177 |
|  | WML change | 0.0239 | 0.0535 | 0.4473 | 0.6550 | -0.0797 | 0.1276 |
|  | Age | -0.2161 | 0.0538 | -4.0196 | **0.0001** | -0.3202 | -0.1119 |
|  | Sex: Male | -0.4828 | 0.1208 | -3.9966 | **0.0001** | -0.7168 | -0.2487 |
|  | Site: ED2 | 0.8102 | 0.2660 | 3.0455 | **0.0025** | 0.2948 | 1.3257 |
|  | Site: ED1 | 0.1168 | 0.2658 | 0.4395 | 0.6606 | -0.3982 | 0.6319 |
|  | Site: DUN | 0.5684 | 0.2958 | 1.9213 | 0.0556 | -0.0048 | 1.1415 |
|  | Site: GLA | 0.5034 | 0.2641 | 1.9063 | 0.0576 | -0.0082 | 1.0150 |
|  | DMT | -0.0208 | 0.1278 | -0.1631 | 0.8705 | -0.2684 | 0.2267 |
| GM paracentral R | Intercept | 0.3019 | 0.2431 | 1.2417 | 0.2153 | -0.1692 | 0.7729 |
|  | Time | -0.0317 | 0.0159 | -1.9976 | **0.0466** | -0.0629 | -0.0006 |
|  | WML change | 0.0404 | 0.0529 | 0.7631 | 0.4460 | -0.0621 | 0.1428 |
|  | Age | -0.2426 | 0.0531 | -4.5695 | **<0.0001** | -0.3454 | -0.1397 |
|  | Sex: Male | -0.5314 | 0.1195 | -4.4473 | **<0.0001** | -0.7629 | -0.2999 |
|  | Site: ED2 | 0.2913 | 0.2632 | 1.1068 | 0.2693 | -0.2186 | 0.8013 |
|  | Site: ED1 | -0.4789 | 0.2629 | -1.8215 | 0.0695 | -0.9883 | 0.0305 |
|  | Site: DUN | 0.0701 | 0.2914 | 0.2407 | 0.8099 | -0.4944 | 0.6346 |
|  | Site: GLA | 0.1176 | 0.2611 | 0.4503 | 0.6528 | -0.3884 | 0.6235 |
|  | DMT | -0.2128 | 0.1266 | -1.6817 | 0.0937 | -0.4580 | 0.0324 |
| GM pars opercularis L | Intercept | 0.4245 | 0.2507 | 1.6936 | 0.0914 | -0.0611 | 0.9102 |
|  | Time | -0.0388 | 0.0101 | -3.8407 | **0.0001** | -0.0586 | -0.0190 |
|  | WML change | 0.0423 | 0.0546 | 0.7744 | 0.4393 | -0.0635 | 0.1480 |
|  | Age | -0.3000 | 0.0548 | -5.4766 | **<0.0001** | -0.4061 | -0.1939 |
|  | Sex: Male | -0.3020 | 0.1238 | -2.4398 | **0.0153** | -0.5418 | -0.0622 |
|  | Site: ED2 | -0.1803 | 0.2713 | -0.6645 | 0.5069 | -0.7060 | 0.3454 |
|  | Site: ED1 | -0.6104 | 0.2711 | -2.2510 | **0.0251** | -1.1357 | -0.0850 |
|  | Site: DUN | -0.5258 | 0.3005 | -1.7498 | 0.0812 | -1.1080 | 0.0564 |
|  | Site: GLA | -0.5041 | 0.2694 | -1.8709 | 0.0623 | -1.0261 | 0.0179 |
|  | DMT | 0.1413 | 0.1304 | 1.0837 | 0.2794 | -0.1113 | 0.3939 |
| GM pars opercularis R | Intercept | 0.1240 | 0.2449 | 0.5064 | 0.6129 | -0.3505 | 0.5986 |
|  | Time | -0.0448 | 0.0115 | -3.9067 | **0.0001** | -0.0673 | -0.0223 |
|  | WML change | -0.0556 | 0.0532 | -1.0449 | 0.2969 | -0.1586 | 0.0475 |
|  | Age | -0.3488 | 0.0534 | -6.5294 | **<0.0001** | -0.4523 | -0.2453 |
|  | Sex: Male | -0.3976 | 0.1208 | -3.2922 | **0.0011** | -0.6316 | -0.1636 |
|  | Site: ED2 | 0.3096 | 0.2647 | 1.1696 | 0.2431 | -0.2033 | 0.8225 |
|  | Site: ED1 | -0.0184 | 0.2648 | -0.0696 | 0.9446 | -0.5315 | 0.4947 |
|  | Site: DUN | -0.0859 | 0.2934 | -0.2928 | 0.7699 | -0.6545 | 0.4827 |
|  | Site: GLA | 0.1962 | 0.2631 | 0.7457 | 0.4564 | -0.3136 | 0.7060 |
|  | DMT | -0.1831 | 0.1270 | -1.4425 | 0.1502 | -0.4292 | 0.0629 |
| GM pars orbitalis L | Intercept | -0.4344 | 0.2481 | -1.7510 | 0.0810 | -0.9151 | 0.0463 |
|  | Time | -0.0341 | 0.0151 | -2.2575 | **0.0247** | -0.0637 | -0.0044 |
|  | WML change | 0.0038 | 0.0538 | 0.0699 | 0.9443 | -0.1006 | 0.1081 |
|  | Age | -0.3512 | 0.0540 | -6.5034 | **<0.0001** | -0.4558 | -0.2466 |
|  | Sex: Male | -0.1206 | 0.1224 | -0.9854 | 0.3252 | -0.3579 | 0.1166 |
|  | Site: ED2 | 0.6188 | 0.2683 | 2.3066 | **0.0217** | 0.0990 | 1.1385 |
|  | Site: ED1 | 0.4228 | 0.2682 | 1.5763 | 0.1160 | -0.0969 | 0.9426 |
|  | Site: DUN | 0.5998 | 0.2972 | 2.0178 | **0.0445** | 0.0238 | 1.1757 |
|  | Site: GLA | 0.4718 | 0.2663 | 1.7714 | 0.0775 | -0.0443 | 0.9878 |
|  | DMT | -0.0045 | 0.1285 | -0.0351 | 0.9720 | -0.2536 | 0.2445 |
| GM pars orbitalis R | Intercept | -0.3067 | 0.2428 | -1.2631 | 0.2075 | -0.7771 | 0.1638 |
|  | Time | -0.0629 | 0.0146 | -4.3127 | **<0.0001** | -0.0915 | -0.0342 |
|  | WML change | 0.0440 | 0.0527 | 0.8337 | 0.4051 | -0.0582 | 0.1462 |
|  | Age | -0.3846 | 0.0529 | -7.2656 | **<0.0001** | -0.4872 | -0.2820 |
|  | Sex: Male | -0.2179 | 0.1192 | -1.8276 | 0.0686 | -0.4489 | 0.0131 |
|  | Site: ED2 | 0.6155 | 0.2627 | 2.3427 | **0.0198** | 0.1065 | 1.1246 |
|  | Site: ED1 | 0.3938 | 0.2627 | 1.4991 | 0.1349 | -0.1152 | 0.9027 |
|  | Site: DUN | 0.2554 | 0.2910 | 0.8777 | 0.3808 | -0.3084 | 0.8191 |
|  | Site: GLA | 0.3908 | 0.2607 | 1.4986 | 0.1350 | -0.1144 | 0.8960 |
|  | DMT | -0.0215 | 0.1263 | -0.1706 | 0.8647 | -0.2663 | 0.2232 |
| GM pars triangularis L | Intercept | -0.1836 | 0.2550 | -0.7200 | 0.4721 | -0.6777 | 0.3105 |
|  | Time | -0.0365 | 0.0116 | -3.1445 | **0.0018** | -0.0593 | -0.0137 |
|  | WML change | 0.0061 | 0.0554 | 0.1099 | 0.9125 | -0.1012 | 0.1134 |
|  | Age | -0.2837 | 0.0555 | -5.1099 | **<0.0001** | -0.3913 | -0.1761 |
|  | Sex: Male | -0.0809 | 0.1258 | -0.6434 | 0.5205 | -0.3247 | 0.1628 |
|  | Site: ED2 | 0.5138 | 0.2755 | 1.8646 | 0.0632 | -0.0201 | 1.0476 |
|  | Site: ED1 | 0.1125 | 0.2758 | 0.4078 | 0.6837 | -0.4219 | 0.6469 |
|  | Site: DUN | 0.1931 | 0.3056 | 0.6319 | 0.5279 | -0.3990 | 0.7853 |
|  | Site: GLA | 0.2035 | 0.2740 | 0.7427 | 0.4582 | -0.3274 | 0.7343 |
|  | DMT | -0.0267 | 0.1321 | -0.2022 | 0.8399 | -0.2826 | 0.2292 |
| GM pars triangularis R | Intercept | 0.0957 | 0.2488 | 0.3847 | 0.7007 | -0.3864 | 0.5779 |
|  | Time | -0.0422 | 0.0113 | -3.7344 | **0.0002** | -0.0644 | -0.0200 |
|  | WML change | -0.0002 | 0.0540 | -0.0040 | 0.9968 | -0.1048 | 0.1044 |
|  | Age | -0.3343 | 0.0542 | -6.1736 | **<0.0001** | -0.4392 | -0.2294 |
|  | Sex: Male | -0.1167 | 0.1222 | -0.9549 | 0.3404 | -0.3535 | 0.1201 |
|  | Site: ED2 | 0.3068 | 0.2690 | 1.1405 | 0.2550 | -0.2144 | 0.8279 |
|  | Site: ED1 | -0.2088 | 0.2693 | -0.7754 | 0.4387 | -0.7307 | 0.3130 |
|  | Site: DUN | -0.1612 | 0.2983 | -0.5403 | 0.5894 | -0.7392 | 0.4169 |
|  | Site: GLA | 0.0598 | 0.2673 | 0.2236 | 0.8232 | -0.4582 | 0.5777 |
|  | DMT | -0.0979 | 0.1290 | -0.7592 | 0.4483 | -0.3477 | 0.1520 |
| GM precentral L | Intercept | 0.0100 | 0.2319 | 0.0430 | 0.9658 | -0.4393 | 0.4592 |
|  | Time | -0.0108 | 0.0180 | -0.5980 | 0.5503 | -0.0462 | 0.0246 |
|  | WML change | 0.0380 | 0.0504 | 0.7545 | 0.4511 | -0.0596 | 0.1356 |
|  | Age | -0.2879 | 0.0507 | -5.6785 | **<0.0001** | -0.3861 | -0.1897 |
|  | Sex: Male | -0.4823 | 0.1149 | -4.1992 | **<0.0001** | -0.7049 | -0.2598 |
|  | Site: ED2 | 0.8098 | 0.2507 | 3.2298 | **0.0014** | 0.3240 | 1.2956 |
|  | Site: ED1 | -0.1567 | 0.2507 | -0.6252 | 0.5323 | -0.6424 | 0.3290 |
|  | Site: DUN | 0.3839 | 0.2779 | 1.3817 | 0.1681 | -0.1545 | 0.9224 |
|  | Site: GLA | 0.4687 | 0.2494 | 1.8795 | 0.0611 | -0.0145 | 0.9519 |
|  | DMT | -0.3427 | 0.1214 | -2.8222 | **0.0051** | -0.5780 | -0.1074 |
| GM precentral R | Intercept | -0.2842 | 0.2421 | -1.1743 | 0.2412 | -0.7533 | 0.1848 |
|  | Time | -0.0040 | 0.0176 | -0.2269 | 0.8206 | -0.0385 | 0.0305 |
|  | WML change | -0.0080 | 0.0525 | -0.1533 | 0.8783 | -0.1097 | 0.0937 |
|  | Age | -0.2266 | 0.0527 | -4.2989 | **<0.0001** | -0.3287 | -0.1245 |
|  | Sex: Male | -0.1916 | 0.1188 | -1.6123 | 0.1079 | -0.4218 | 0.0386 |
|  | Site: ED2 | 1.0176 | 0.2619 | 3.8860 | **0.0001** | 0.5102 | 1.5249 |
|  | Site: ED1 | 0.0594 | 0.2617 | 0.2270 | 0.8206 | -0.4476 | 0.5664 |
|  | Site: DUN | 0.6424 | 0.2900 | 2.2152 | **0.0275** | 0.0805 | 1.2043 |
|  | Site: GLA | 0.6378 | 0.2599 | 2.4545 | **0.0147** | 0.1343 | 1.1413 |
|  | DMT | -0.3010 | 0.1259 | -2.3912 | **0.0174** | -0.5448 | -0.0571 |
| GM rostral anterior cingulate L | Intercept | -0.2926 | 0.2543 | -1.1507 | 0.2508 | -0.7852 | 0.2001 |
|  | Time | -0.0472 | 0.0124 | -3.7977 | **0.0002** | -0.0715 | -0.0228 |
|  | WML change | -0.0377 | 0.0553 | -0.6806 | 0.4966 | -0.1449 | 0.0695 |
|  | Age | -0.2847 | 0.0555 | -5.1256 | **<0.0001** | -0.3923 | -0.1771 |
|  | Sex: Male | -0.0834 | 0.1255 | -0.6644 | 0.5069 | -0.3265 | 0.1598 |
|  | Site: ED2 | 0.4084 | 0.2755 | 1.4825 | 0.1392 | -0.1253 | 0.9422 |
|  | Site: ED1 | 0.1076 | 0.2752 | 0.3909 | 0.6961 | -0.4256 | 0.6407 |
|  | Site: DUN | 0.4027 | 0.3050 | 1.3204 | 0.1877 | -0.1882 | 0.9936 |
|  | Site: GLA | 0.2830 | 0.2738 | 1.0335 | 0.3022 | -0.2475 | 0.8135 |
|  | DMT | 0.0921 | 0.1334 | 0.6902 | 0.4906 | -0.1664 | 0.3506 |
| GM rostral anterior cingulate R | Intercept | -0.3772 | 0.2592 | -1.4548 | 0.1468 | -0.8795 | 0.1252 |
|  | Time | -0.0195 | 0.0103 | -1.8930 | 0.0593 | -0.0398 | 0.0007 |
|  | WML change | 0.0319 | 0.0562 | 0.5686 | 0.5700 | -0.0769 | 0.1408 |
|  | Age | -0.2114 | 0.0564 | -3.7502 | **0.0002** | -0.3206 | -0.1022 |
|  | Sex: Male | 0.0322 | 0.1273 | 0.2532 | 0.8003 | -0.2144 | 0.2788 |
|  | Site: ED2 | 0.5600 | 0.2802 | 1.9985 | **0.0465** | 0.0170 | 1.1029 |
|  | Site: ED1 | 0.1456 | 0.2805 | 0.5193 | 0.6040 | -0.3978 | 0.6891 |
|  | Site: DUN | 0.7071 | 0.3108 | 2.2752 | **0.0236** | 0.1049 | 1.3093 |
|  | Site: GLA | 0.4455 | 0.2785 | 1.6000 | 0.1106 | -0.0940 | 0.9851 |
|  | DMT | -0.0393 | 0.1343 | -0.2928 | 0.7699 | -0.2995 | 0.2209 |
| GM rostral middle frontal L | Intercept | -0.4954 | 0.2396 | -2.0679 | **0.0395** | -0.9595 | -0.0313 |
|  | Time | -0.0505 | 0.0143 | -3.5410 | **0.0005** | -0.0785 | -0.0225 |
|  | WML change | -0.0256 | 0.0522 | -0.4914 | 0.6235 | -0.1267 | 0.0754 |
|  | Age | -0.3396 | 0.0523 | -6.4890 | **<0.0001** | -0.4410 | -0.2382 |
|  | Sex: Male | 0.0385 | 0.1184 | 0.3255 | 0.7450 | -0.1908 | 0.2679 |
|  | Site: ED2 | 0.8387 | 0.2593 | 3.2345 | **0.0014** | 0.3363 | 1.3411 |
|  | Site: ED1 | 0.2314 | 0.2592 | 0.8930 | 0.3726 | -0.2707 | 0.7336 |
|  | Site: DUN | 0.3492 | 0.2881 | 1.2119 | 0.2265 | -0.2090 | 0.9075 |
|  | Site: GLA | 0.7968 | 0.2574 | 3.0953 | **0.0022** | 0.2981 | 1.2955 |
|  | DMT | -0.1017 | 0.1243 | -0.8182 | 0.4139 | -0.3425 | 0.1391 |
| GM rostral middle frontal R | Intercept | -0.1773 | 0.2446 | -0.7251 | 0.4690 | -0.6512 | 0.2965 |
|  | Time | -0.0317 | 0.0150 | -2.1159 | **0.0351** | -0.0611 | -0.0023 |
|  | WML change | 0.0681 | 0.0532 | 1.2798 | 0.2016 | -0.0350 | 0.1711 |
|  | Age | -0.3565 | 0.0533 | -6.6904 | **<0.0001** | -0.4598 | -0.2533 |
|  | Sex: Male | -0.0628 | 0.1212 | -0.5181 | 0.6048 | -0.2977 | 0.1721 |
|  | Site: ED2 | 0.4258 | 0.2644 | 1.6103 | 0.1084 | -0.0865 | 0.9380 |
|  | Site: ED1 | -0.1459 | 0.2644 | -0.5520 | 0.5814 | -0.6581 | 0.3663 |
|  | Site: DUN | 0.1472 | 0.2930 | 0.5023 | 0.6158 | -0.4205 | 0.7148 |
|  | Site: GLA | 0.2216 | 0.2628 | 0.8431 | 0.3998 | -0.2876 | 0.7307 |
|  | DMT | 0.0641 | 0.1267 | 0.5055 | 0.6136 | -0.1815 | 0.3096 |
| GM superior frontal L | Intercept | 0.0761 | 0.2237 | 0.3400 | 0.7341 | -0.3573 | 0.5094 |
|  | Time | -0.0624 | 0.0195 | -3.1906 | **0.0016** | -0.1007 | -0.0240 |
|  | WML change | 0.0033 | 0.0486 | 0.0685 | 0.9454 | -0.0909 | 0.0976 |
|  | Age | -0.3830 | 0.0488 | -7.8474 | **<0.0001** | -0.4775 | -0.2884 |
|  | Sex: Male | -0.1630 | 0.1099 | -1.4838 | 0.1389 | -0.3759 | 0.0498 |
|  | Site: ED2 | 0.5120 | 0.2422 | 2.1138 | **0.0354** | 0.0427 | 0.9814 |
|  | Site: ED1 | -0.5927 | 0.2418 | -2.4509 | **0.0148** | -1.0612 | -0.1242 |
|  | Site: DUN | -0.0135 | 0.2680 | -0.0504 | 0.9599 | -0.5327 | 0.5057 |
|  | Site: GLA | 0.1512 | 0.2402 | 0.6294 | 0.5296 | -0.3142 | 0.6165 |
|  | DMT | -0.0438 | 0.1165 | -0.3761 | 0.7071 | -0.2695 | 0.1819 |
| GM superior frontal R | Intercept | 0.2692 | 0.2198 | 1.2248 | 0.2216 | -0.1567 | 0.6951 |
|  | Time | -0.0348 | 0.0190 | -1.8294 | 0.0683 | -0.0721 | 0.0025 |
|  | WML change | -0.0059 | 0.0477 | -0.1227 | 0.9024 | -0.0984 | 0.0866 |
|  | Age | -0.3966 | 0.0479 | -8.2843 | **<0.0001** | -0.4894 | -0.3039 |
|  | Sex: Male | -0.2636 | 0.1084 | -2.4322 | **0.0156** | -0.4736 | -0.0536 |
|  | Site: ED2 | 0.4023 | 0.2375 | 1.6939 | 0.0913 | -0.0579 | 0.8625 |
|  | Site: ED1 | -0.6951 | 0.2376 | -2.9254 | **0.0037** | -1.1555 | -0.2347 |
|  | Site: DUN | -0.4466 | 0.2633 | -1.6962 | 0.0909 | -0.9568 | 0.0636 |
|  | Site: GLA | -0.1134 | 0.2361 | -0.4805 | 0.6312 | -0.5708 | 0.3439 |
|  | DMT | -0.0281 | 0.1139 | -0.2464 | 0.8055 | -0.2487 | 0.1926 |
| NAWM caudal anterior cingulate L | Intercept | -0.0546 | 0.2577 | -0.2119 | 0.8323 | -0.5538 | 0.4446 |
|  | Time | -0.0743 | 0.0134 | -5.5530 | **<0.0001** | -0.1006 | -0.0481 |
|  | WML change | -0.0577 | 0.0560 | -1.0307 | 0.3035 | -0.1662 | 0.0508 |
|  | Age | -0.0026 | 0.0562 | -0.0456 | 0.9637 | -0.1114 | 0.1063 |
|  | Sex: Male | -0.2136 | 0.1266 | -1.6878 | 0.0925 | -0.4589 | 0.0316 |
|  | Site: ED2 | 0.6365 | 0.2788 | 2.2826 | **0.0231** | 0.0962 | 1.1767 |
|  | Site: ED1 | -0.1178 | 0.2786 | -0.4228 | 0.6727 | -0.6577 | 0.4221 |
|  | Site: DUN | 0.2964 | 0.3088 | 0.9598 | 0.3379 | -0.3019 | 0.8947 |
|  | Site: GLA | 0.0907 | 0.2768 | 0.3276 | 0.7435 | -0.4456 | 0.6269 |
|  | DMT | -0.0494 | 0.1340 | -0.3685 | 0.7128 | -0.3090 | 0.2102 |
| NAWM caudal anterior cingulate R | Intercept | 0.1827 | 0.2536 | 0.7204 | 0.4719 | -0.3087 | 0.6741 |
|  | Time | -0.0863 | 0.0134 | -6.4445 | **<0.0001** | -0.1126 | -0.0600 |
|  | WML change | -0.0629 | 0.0551 | -1.1402 | 0.2551 | -0.1697 | 0.0440 |
|  | Age | -0.1143 | 0.0553 | -2.0681 | **0.0395** | -0.2213 | -0.0072 |
|  | Sex: Male | -0.4017 | 0.1246 | -3.2234 | **0.0014** | -0.6431 | -0.1602 |
|  | Site: ED2 | 0.2227 | 0.2743 | 0.8119 | 0.4175 | -0.3087 | 0.7541 |
|  | Site: ED1 | -0.2909 | 0.2745 | -1.0596 | 0.2902 | -0.8228 | 0.2410 |
|  | Site: DUN | 0.4958 | 0.3041 | 1.6304 | 0.1040 | -0.0934 | 1.0850 |
|  | Site: GLA | -0.1729 | 0.2725 | -0.6345 | 0.5263 | -0.7009 | 0.3551 |
|  | DMT | -0.0114 | 0.1316 | -0.0870 | 0.9308 | -0.2664 | 0.2435 |
| NAWM caudal middle frontal L | Intercept | 0.4091 | 0.2559 | 1.5988 | 0.1109 | -0.0867 | 0.9049 |
|  | Time | -0.0185 | 0.0089 | -2.0926 | **0.0372** | -0.0359 | -0.0011 |
|  | WML change | -0.0345 | 0.0555 | -0.6223 | 0.5342 | -0.1420 | 0.0730 |
|  | Age | -0.0347 | 0.0557 | -0.6227 | 0.5340 | -0.1427 | 0.0733 |
|  | Sex: Male | -0.3901 | 0.1263 | -3.0897 | **0.0022** | -0.6347 | -0.1455 |
|  | Site: ED2 | 0.1558 | 0.2764 | 0.5637 | 0.5734 | -0.3797 | 0.6913 |
|  | Site: ED1 | -0.6478 | 0.2770 | -2.3386 | **0.0200** | -1.1846 | -0.1111 |
|  | Site: DUN | -0.1679 | 0.3066 | -0.5478 | 0.5842 | -0.7620 | 0.4261 |
|  | Site: GLA | -0.1602 | 0.2747 | -0.5832 | 0.5602 | -0.6924 | 0.3720 |
|  | DMT | -0.1480 | 0.1327 | -1.1156 | 0.2655 | -0.4051 | 0.1091 |
| NAWM caudal middle frontal R | Intercept | 0.6168 | 0.2596 | 2.3760 | **0.0181** | 0.1138 | 1.1197 |
|  | Time | -0.0254 | 0.0093 | -2.7177 | **0.0069** | -0.0437 | -0.0071 |
|  | WML change | -0.0677 | 0.0563 | -1.2024 | 0.2301 | -0.1768 | 0.0414 |
|  | Age | 0.0003 | 0.0565 | 0.0052 | 0.9959 | -0.1092 | 0.1098 |
|  | Sex: Male | -0.4863 | 0.1275 | -3.8147 | **0.0002** | -0.7333 | -0.2393 |
|  | Site: ED2 | -0.3278 | 0.2807 | -1.1678 | 0.2438 | -0.8718 | 0.2161 |
|  | Site: ED1 | -0.7947 | 0.2809 | -2.8295 | **0.0050** | -1.3388 | -0.2505 |
|  | Site: DUN | -0.5587 | 0.3112 | -1.7953 | 0.0736 | -1.1617 | 0.0443 |
|  | Site: GLA | -0.5071 | 0.2789 | -1.8183 | 0.0700 | -1.0474 | 0.0333 |
|  | DMT | 0.0449 | 0.1346 | 0.3333 | 0.7391 | -0.2159 | 0.3056 |
| NAWM frontal pole L | Intercept | 0.1641 | 0.2615 | 0.6276 | 0.5307 | -0.3425 | 0.6707 |
|  | Time | -0.0111 | 0.0243 | -0.4555 | 0.6491 | -0.0589 | 0.0367 |
|  | WML change | 0.0571 | 0.0568 | 1.0051 | 0.3157 | -0.0530 | 0.1672 |
|  | Age | -0.0156 | 0.0571 | -0.2734 | 0.7847 | -0.1263 | 0.0950 |
|  | Sex: Male | 0.0707 | 0.1283 | 0.5510 | 0.5820 | -0.1779 | 0.3194 |
|  | Site: ED2 | 0.0545 | 0.2827 | 0.1927 | 0.8474 | -0.4932 | 0.6022 |
|  | Site: ED1 | -0.2672 | 0.2830 | -0.9441 | 0.3459 | -0.8155 | 0.2811 |
|  | Site: DUN | -0.2381 | 0.3132 | -0.7603 | 0.4477 | -0.8448 | 0.3686 |
|  | Site: GLA | 0.0528 | 0.2807 | 0.1882 | 0.8508 | -0.4910 | 0.5967 |
|  | DMT | -0.1625 | 0.1365 | -1.1909 | 0.2346 | -0.4269 | 0.1019 |
| NAWM frontal pole R | Intercept | 0.4750 | 0.2575 | 1.8448 | 0.0660 | -0.0239 | 0.9738 |
|  | Time | 0.0042 | 0.0199 | 0.2122 | 0.8321 | -0.0348 | 0.0432 |
|  | WML change | 0.0266 | 0.0557 | 0.4767 | 0.6339 | -0.0814 | 0.1346 |
|  | Age | 0.0089 | 0.0559 | 0.1593 | 0.8735 | -0.0995 | 0.1173 |
|  | Sex: Male | -0.3065 | 0.1263 | -2.4268 | **0.0158** | -0.5513 | -0.0618 |
|  | Site: ED2 | -0.4368 | 0.2781 | -1.5706 | 0.1173 | -0.9756 | 0.1021 |
|  | Site: ED1 | -0.6965 | 0.2784 | -2.5021 | **0.0129** | -1.2358 | -0.1571 |
|  | Site: DUN | -0.9031 | 0.3085 | -2.9278 | **0.0037** | -1.5008 | -0.3054 |
|  | Site: GLA | -0.2979 | 0.2764 | -1.0780 | 0.2819 | -0.8335 | 0.2376 |
|  | DMT | 0.0989 | 0.1333 | 0.7420 | 0.4586 | -0.1593 | 0.3571 |
| NAWM lateral orbito frontal L | Intercept | -0.2997 | 0.2651 | -1.1304 | 0.2592 | -0.8133 | 0.2139 |
|  | Time | -0.0730 | 0.0109 | -6.6879 | **<0.0001** | -0.0945 | -0.0516 |
|  | WML change | -0.0047 | 0.0576 | -0.0808 | 0.9357 | -0.1162 | 0.1069 |
|  | Age | 0.0423 | 0.0578 | 0.7316 | 0.4650 | -0.0697 | 0.1542 |
|  | Sex: Male | -0.1921 | 0.1308 | -1.4682 | 0.1431 | -0.4456 | 0.0614 |
|  | Site: ED2 | 0.4845 | 0.2867 | 1.6897 | 0.0921 | -0.0710 | 1.0400 |
|  | Site: ED1 | 0.1565 | 0.2868 | 0.5456 | 0.5858 | -0.3992 | 0.7122 |
|  | Site: DUN | 0.3309 | 0.3174 | 1.0424 | 0.2980 | -0.2841 | 0.9459 |
|  | Site: GLA | 0.1179 | 0.2847 | 0.4141 | 0.6791 | -0.4336 | 0.6694 |
|  | DMT | 0.2128 | 0.1379 | 1.5437 | 0.1237 | -0.0543 | 0.4800 |
| NAWM lateral orbito frontal R | Intercept | 0.1456 | 0.2577 | 0.5649 | 0.5726 | -0.3537 | 0.6448 |
|  | Time | -0.0109 | 0.0221 | -0.4955 | 0.6206 | -0.0543 | 0.0324 |
|  | WML change | 0.0594 | 0.0559 | 1.0635 | 0.2884 | -0.0488 | 0.1676 |
|  | Age | 0.0587 | 0.0560 | 1.0479 | 0.2955 | -0.0499 | 0.1673 |
|  | Sex: Male | -0.2856 | 0.1264 | -2.2587 | **0.0246** | -0.5305 | -0.0406 |
|  | Site: ED2 | 0.0297 | 0.2787 | 0.1064 | 0.9153 | -0.5103 | 0.5696 |
|  | Site: ED1 | -0.3741 | 0.2784 | -1.3439 | 0.1800 | -0.9136 | 0.1653 |
|  | Site: DUN | -0.4304 | 0.3085 | -1.3952 | 0.1640 | -1.0282 | 0.1673 |
|  | Site: GLA | -0.4118 | 0.2766 | -1.4885 | 0.1377 | -0.9478 | 0.1242 |
|  | DMT | 0.2948 | 0.1338 | 2.2032 | **0.0283** | 0.0355 | 0.5540 |
| NAWM medial orbito frontal L | Intercept | 0.4148 | 0.2683 | 1.5460 | 0.1231 | -0.1051 | 0.9348 |
|  | Time | -0.0148 | 0.0223 | -0.6654 | 0.5063 | -0.0587 | 0.0290 |
|  | WML change | 0.0074 | 0.0565 | 0.1303 | 0.8964 | -0.1021 | 0.1168 |
|  | Age | -0.0058 | 0.0567 | -0.1028 | 0.9182 | -0.1156 | 0.1040 |
|  | Sex: Male | 0.0447 | 0.1283 | 0.3485 | 0.7277 | -0.2039 | 0.2933 |
|  | Site: ED2 | -0.1255 | 0.2886 | -0.4348 | 0.6640 | -0.6846 | 0.4336 |
|  | Site: ED1 | -0.3142 | 0.2887 | -1.0884 | 0.2773 | -0.8737 | 0.2452 |
|  | Site: DUN | -0.5932 | 0.3184 | -1.8629 | 0.0634 | -1.2102 | 0.0238 |
|  | Site: GLA | -0.1332 | 0.2866 | -0.4647 | 0.6424 | -0.6885 | 0.4221 |
|  | DMT | -0.2712 | 0.1350 | -2.0094 | **0.0454** | -0.5328 | -0.0097 |
| NAWM medial orbito frontal R | Intercept | 0.0828 | 0.2614 | 0.3166 | 0.7518 | -0.4238 | 0.5893 |
|  | Time | -0.0301 | 0.0214 | -1.4092 | 0.1598 | -0.0721 | 0.0118 |
|  | WML change | -0.0386 | 0.0567 | -0.6806 | 0.4966 | -0.1484 | 0.0712 |
|  | Age | 0.0615 | 0.0569 | 1.0810 | 0.2805 | -0.0487 | 0.1717 |
|  | Sex: Male | 0.1054 | 0.1283 | 0.8209 | 0.4124 | -0.1433 | 0.3540 |
|  | Site: ED2 | 0.1711 | 0.2824 | 0.6060 | 0.5450 | -0.3760 | 0.7182 |
|  | Site: ED1 | -0.2776 | 0.2826 | -0.9821 | 0.3268 | -0.8252 | 0.2700 |
|  | Site: DUN | -0.3188 | 0.3142 | -1.0146 | 0.3111 | -0.9276 | 0.2900 |
|  | Site: GLA | -0.2515 | 0.2806 | -0.8964 | 0.3708 | -0.7953 | 0.2922 |
|  | DMT | 0.0860 | 0.1354 | 0.6355 | 0.5256 | -0.1763 | 0.3483 |
| NAWM paracentral L | Intercept | 0.2800 | 0.2608 | 1.0735 | 0.2839 | -0.2254 | 0.7854 |
|  | Time | -0.0505 | 0.0140 | -3.6198 | **0.0003** | -0.0779 | -0.0231 |
|  | WML change | -0.0497 | 0.0569 | -0.8742 | 0.3827 | -0.1599 | 0.0605 |
|  | Age | -0.1183 | 0.0572 | -2.0676 | **0.0395** | -0.2292 | -0.0074 |
|  | Sex: Male | -0.3111 | 0.1285 | -2.4211 | **0.0161** | -0.5600 | -0.0621 |
|  | Site: ED2 | 0.0192 | 0.2819 | 0.0682 | 0.9456 | -0.5270 | 0.5655 |
|  | Site: ED1 | -0.4120 | 0.2827 | -1.4574 | 0.1460 | -0.9598 | 0.1357 |
|  | Site: DUN | 0.0163 | 0.3130 | 0.0520 | 0.9586 | -0.5902 | 0.6227 |
|  | Site: GLA | -0.2597 | 0.2808 | -0.9249 | 0.3557 | -0.8037 | 0.2843 |
|  | DMT | 0.0165 | 0.1358 | 0.1219 | 0.9031 | -0.2465 | 0.2796 |
| NAWM paracentral R | Intercept | 0.7111 | 0.2614 | 2.7207 | **0.0069** | 0.2047 | 1.2174 |
|  | Time | -0.0391 | 0.0136 | -2.8630 | **0.0045** | -0.0658 | -0.0123 |
|  | WML change | -0.0817 | 0.0570 | -1.4318 | 0.1532 | -0.1922 | 0.0288 |
|  | Age | -0.0849 | 0.0574 | -1.4801 | 0.1399 | -0.1960 | 0.0262 |
|  | Sex: Male | -0.3016 | 0.1287 | -2.3424 | **0.0198** | -0.5510 | -0.0521 |
|  | Site: ED2 | -0.5165 | 0.2825 | -1.8288 | 0.0684 | -1.0638 | 0.0307 |
|  | Site: ED1 | -0.7840 | 0.2834 | -2.7667 | **0.0060** | -1.3331 | -0.2350 |
|  | Site: DUN | -0.7528 | 0.3148 | -2.3912 | **0.0174** | -1.3627 | -0.1429 |
|  | Site: GLA | -0.5299 | 0.2812 | -1.8841 | 0.0605 | -1.0748 | 0.0150 |
|  | DMT | -0.0459 | 0.1362 | -0.3371 | 0.7363 | -0.3098 | 0.2180 |
| NAWM pars opercularis L | Intercept | 0.4362 | 0.2732 | 1.5969 | 0.1113 | -0.0931 | 0.9655 |
|  | Time | -0.0279 | 0.0093 | -2.9909 | **0.0030** | -0.0461 | -0.0096 |
|  | WML change | 0.0413 | 0.0576 | 0.7167 | 0.4741 | -0.0703 | 0.1529 |
|  | Age | -0.0879 | 0.0578 | -1.5200 | 0.1295 | -0.1999 | 0.0241 |
|  | Sex: Male | -0.1113 | 0.1302 | -0.8548 | 0.3933 | -0.3637 | 0.1410 |
|  | Site: ED2 | -0.2673 | 0.2961 | -0.9027 | 0.3674 | -0.8411 | 0.3065 |
|  | Site: ED1 | -0.6926 | 0.2961 | -2.3388 | **0.0200** | -1.2663 | -0.1188 |
|  | Site: DUN | -0.5941 | 0.3272 | -1.8157 | 0.0704 | -1.2281 | 0.0399 |
|  | Site: GLA | -0.5009 | 0.2952 | -1.6968 | 0.0908 | -1.0728 | 0.0711 |
|  | DMT | 0.1181 | 0.1386 | 0.8519 | 0.3949 | -0.1505 | 0.3866 |
| NAWM pars opercularis R | Intercept | -0.0677 | 0.2617 | -0.2589 | 0.7959 | -0.5747 | 0.4392 |
|  | Time | -0.0282 | 0.0085 | -3.3114 | **0.0010** | -0.0449 | -0.0115 |
|  | WML change | 0.0218 | 0.0568 | 0.3844 | 0.7009 | -0.0882 | 0.1318 |
|  | Age | -0.1760 | 0.0570 | -3.0880 | **0.0022** | -0.2864 | -0.0656 |
|  | Sex: Male | -0.1366 | 0.1285 | -1.0627 | 0.2887 | -0.3857 | 0.1125 |
|  | Site: ED2 | 0.4992 | 0.2828 | 1.7651 | 0.0786 | -0.0488 | 1.0471 |
|  | Site: ED1 | 0.0111 | 0.2831 | 0.0393 | 0.9687 | -0.5374 | 0.5596 |
|  | Site: DUN | 0.2664 | 0.3137 | 0.8491 | 0.3965 | -0.3415 | 0.8742 |
|  | Site: GLA | 0.2713 | 0.2811 | 0.9650 | 0.3353 | -0.2734 | 0.8160 |
|  | DMT | -0.1797 | 0.1355 | -1.3259 | 0.1859 | -0.4424 | 0.0829 |
| NAWM pars orbitalis L | Intercept | 0.0445 | 0.2628 | 0.1694 | 0.8656 | -0.4646 | 0.5536 |
|  | Time | -0.0245 | 0.0175 | -1.4010 | 0.1622 | -0.0588 | 0.0098 |
|  | WML change | 0.1149 | 0.0571 | 2.0139 | **0.0449** | 0.0044 | 0.2255 |
|  | Age | -0.0159 | 0.0574 | -0.2769 | 0.7821 | -0.1270 | 0.0953 |
|  | Sex: Male | 0.2015 | 0.1290 | 1.5614 | 0.1195 | -0.0485 | 0.4514 |
|  | Site: ED2 | -0.0353 | 0.2838 | -0.1245 | 0.9010 | -0.5852 | 0.5145 |
|  | Site: ED1 | -0.3743 | 0.2843 | -1.3166 | 0.1890 | -0.9252 | 0.1765 |
|  | Site: DUN | 0.0291 | 0.3148 | 0.0923 | 0.9265 | -0.5809 | 0.6390 |
|  | Site: GLA | -0.0069 | 0.2821 | -0.0246 | 0.9804 | -0.5536 | 0.5397 |
|  | DMT | 0.0168 | 0.1363 | 0.1231 | 0.9021 | -0.2474 | 0.2810 |
| NAWM pars orbitalis R | Intercept | 0.0102 | 0.2605 | 0.0391 | 0.9689 | -0.4945 | 0.5149 |
|  | Time | -0.0373 | 0.0176 | -2.1198 | **0.0348** | -0.0719 | -0.0028 |
|  | WML change | 0.0279 | 0.0567 | 0.4916 | 0.6234 | -0.0819 | 0.1377 |
|  | Age | -0.0071 | 0.0569 | -0.1244 | 0.9010 | -0.1173 | 0.1031 |
|  | Sex: Male | 0.2535 | 0.1280 | 1.9799 | **0.0486** | 0.0054 | 0.5015 |
|  | Site: ED2 | 0.2877 | 0.2818 | 1.0207 | 0.3082 | -0.2584 | 0.8337 |
|  | Site: ED1 | -0.2475 | 0.2818 | -0.8783 | 0.3805 | -0.7934 | 0.2984 |
|  | Site: DUN | -0.3114 | 0.3122 | -0.9974 | 0.3194 | -0.9163 | 0.2935 |
|  | Site: GLA | -0.1260 | 0.2799 | -0.4500 | 0.6530 | -0.6682 | 0.4163 |
|  | DMT | 0.0308 | 0.1352 | 0.2275 | 0.8202 | -0.2312 | 0.2927 |
| NAWM pars triangularis L | Intercept | -0.0010 | 0.2656 | -0.0039 | 0.9969 | -0.5158 | 0.5137 |
|  | Time | -0.0155 | 0.0104 | -1.4843 | 0.1387 | -0.0359 | 0.0050 |
|  | WML change | -0.0417 | 0.0575 | -0.7246 | 0.4692 | -0.1532 | 0.0698 |
|  | Age | 0.0128 | 0.0578 | 0.2217 | 0.8247 | -0.0991 | 0.1247 |
|  | Sex: Male | 0.1786 | 0.1304 | 1.3696 | 0.1718 | -0.0741 | 0.4313 |
|  | Site: ED2 | 0.1212 | 0.2871 | 0.4223 | 0.6731 | -0.4351 | 0.6775 |
|  | Site: ED1 | -0.3276 | 0.2874 | -1.1399 | 0.2552 | -0.8844 | 0.2293 |
|  | Site: DUN | -0.1471 | 0.3184 | -0.4618 | 0.6445 | -0.7641 | 0.4700 |
|  | Site: GLA | -0.1835 | 0.2853 | -0.6430 | 0.5207 | -0.7363 | 0.3694 |
|  | DMT | 0.1365 | 0.1376 | 0.9919 | 0.3220 | -0.1301 | 0.4031 |
| NAWM pars triangularis R | Intercept | -0.0583 | 0.2656 | -0.2194 | 0.8265 | -0.5729 | 0.4564 |
|  | Time | -0.0072 | 0.0109 | -0.6627 | 0.5080 | -0.0285 | 0.0141 |
|  | WML change | -0.0451 | 0.0576 | -0.7833 | 0.4341 | -0.1567 | 0.0665 |
|  | Age | -0.0453 | 0.0578 | -0.7827 | 0.4344 | -0.1573 | 0.0668 |
|  | Sex: Male | -0.0870 | 0.1310 | -0.6643 | 0.5070 | -0.3407 | 0.1667 |
|  | Site: ED2 | 0.4496 | 0.2870 | 1.5664 | 0.1183 | -0.1065 | 1.0056 |
|  | Site: ED1 | -0.0055 | 0.2873 | -0.0192 | 0.9847 | -0.5621 | 0.5511 |
|  | Site: DUN | 0.1430 | 0.3183 | 0.4492 | 0.6536 | -0.4738 | 0.7598 |
|  | Site: GLA | 0.1390 | 0.2854 | 0.4872 | 0.6265 | -0.4139 | 0.6919 |
|  | DMT | -0.1166 | 0.1376 | -0.8475 | 0.3974 | -0.3831 | 0.1500 |
| NAWM precentral L | Intercept | 0.5516 | 0.2590 | 2.1299 | **0.0340** | 0.0499 | 1.0534 |
|  | Time | -0.0262 | 0.0150 | -1.7514 | 0.0809 | -0.0556 | 0.0032 |
|  | WML change | 0.0475 | 0.0563 | 0.8432 | 0.3998 | -0.0617 | 0.1567 |
|  | Age | -0.0013 | 0.0566 | -0.0225 | 0.9820 | -0.1109 | 0.1083 |
|  | Sex: Male | -0.3834 | 0.1290 | -2.9724 | **0.0032** | -0.6333 | -0.1335 |
|  | Site: ED2 | -0.0738 | 0.2806 | -0.2630 | 0.7927 | -0.6176 | 0.4699 |
|  | Site: ED1 | -0.6047 | 0.2802 | -2.1581 | **0.0317** | -1.1476 | -0.0618 |
|  | Site: DUN | -0.2039 | 0.3100 | -0.6577 | 0.5112 | -0.8046 | 0.3968 |
|  | Site: GLA | -0.2982 | 0.2779 | -1.0732 | 0.2840 | -0.8365 | 0.2402 |
|  | DMT | -0.2044 | 0.1347 | -1.5172 | 0.1302 | -0.4654 | 0.0566 |
| NAWM precentral R | Intercept | 0.2384 | 0.2711 | 0.8797 | 0.3797 | -0.2867 | 0.7636 |
|  | Time | -0.0244 | 0.0134 | -1.8166 | 0.0702 | -0.0508 | 0.0020 |
|  | WML change | -0.0208 | 0.0568 | -0.3657 | 0.7148 | -0.1308 | 0.0893 |
|  | Age | 0.0636 | 0.0570 | 1.1153 | 0.2656 | -0.0469 | 0.1741 |
|  | Sex: Male | -0.1360 | 0.1283 | -1.0595 | 0.2902 | -0.3846 | 0.1127 |
|  | Site: ED2 | 0.2922 | 0.2904 | 1.0059 | 0.3153 | -0.2706 | 0.8549 |
|  | Site: ED1 | -0.2268 | 0.2904 | -0.7811 | 0.4354 | -0.7893 | 0.3358 |
|  | Site: DUN | 0.1400 | 0.3195 | 0.4382 | 0.6615 | -0.4791 | 0.7591 |
|  | Site: GLA | -0.1410 | 0.2879 | -0.4899 | 0.6246 | -0.6989 | 0.4168 |
|  | DMT | -0.2232 | 0.1358 | -1.6439 | 0.1012 | -0.4863 | 0.0399 |
| NAWM rostral anterior cingulate L | Intercept | 0.4936 | 0.2642 | 1.8679 | 0.0627 | -0.0184 | 1.0056 |
|  | Time | -0.0329 | 0.0174 | -1.8938 | 0.0592 | -0.0670 | 0.0012 |
|  | WML change | -0.0732 | 0.0572 | -1.2788 | 0.2019 | -0.1841 | 0.0377 |
|  | Age | 0.0106 | 0.0574 | 0.1853 | 0.8531 | -0.1006 | 0.1219 |
|  | Sex: Male | -0.1753 | 0.1297 | -1.3518 | 0.1774 | -0.4266 | 0.0760 |
|  | Site: ED2 | -0.3384 | 0.2855 | -1.1852 | 0.2368 | -0.8916 | 0.2148 |
|  | Site: ED1 | -0.6258 | 0.2858 | -2.1899 | **0.0293** | -1.1795 | -0.0721 |
|  | Site: DUN | -0.4198 | 0.3167 | -1.3258 | 0.1859 | -1.0334 | 0.1938 |
|  | Site: GLA | -0.5496 | 0.2837 | -1.9369 | 0.0537 | -1.0993 | 0.0002 |
|  | DMT | 0.0624 | 0.1368 | 0.4562 | 0.6485 | -0.2027 | 0.3275 |
| NAWM rostral anterior cingulate R | Intercept | 0.2556 | 0.2609 | 0.9798 | 0.3279 | -0.2499 | 0.7611 |
|  | Time | -0.0303 | 0.0145 | -2.0852 | **0.0379** | -0.0588 | -0.0018 |
|  | WML change | -0.0060 | 0.0566 | -0.1061 | 0.9156 | -0.1157 | 0.1037 |
|  | Age | -0.1136 | 0.0568 | -1.9989 | **0.0465** | -0.2237 | -0.0035 |
|  | Sex: Male | -0.1430 | 0.1281 | -1.1165 | 0.2651 | -0.3913 | 0.1052 |
|  | Site: ED2 | -0.1588 | 0.2819 | -0.5632 | 0.5737 | -0.7050 | 0.3874 |
|  | Site: ED1 | -0.5535 | 0.2822 | -1.9617 | 0.0507 | -1.1003 | -0.0068 |
|  | Site: DUN | -0.6639 | 0.3127 | -2.1234 | **0.0345** | -1.2698 | -0.0581 |
|  | Site: GLA | -0.2507 | 0.2802 | -0.8947 | 0.3716 | -0.7937 | 0.2922 |
|  | DMT | 0.1755 | 0.1351 | 1.2992 | 0.1949 | -0.0862 | 0.4373 |
| NAWM rostral middle frontal L | Intercept | -0.1527 | 0.2650 | -0.5764 | 0.5648 | -0.6662 | 0.3607 |
|  | Time | -0.0311 | 0.0100 | -3.1023 | **0.0021** | -0.0507 | -0.0114 |
|  | WML change | -0.0520 | 0.0575 | -0.9043 | 0.3666 | -0.1633 | 0.0594 |
|  | Age | -0.0473 | 0.0577 | -0.8199 | 0.4129 | -0.1592 | 0.0645 |
|  | Sex: Male | 0.1069 | 0.1302 | 0.8215 | 0.4120 | -0.1453 | 0.3592 |
|  | Site: ED2 | 0.3704 | 0.2863 | 1.2937 | 0.1968 | -0.1843 | 0.9251 |
|  | Site: ED1 | -0.1318 | 0.2866 | -0.4597 | 0.6460 | -0.6870 | 0.4235 |
|  | Site: DUN | 0.1913 | 0.3176 | 0.6024 | 0.5473 | -0.4240 | 0.8066 |
|  | Site: GLA | 0.1852 | 0.2848 | 0.6502 | 0.5160 | -0.3666 | 0.7370 |
|  | DMT | -0.0033 | 0.1374 | -0.0240 | 0.9809 | -0.2694 | 0.2628 |
| NAWM rostral middle frontal R | Intercept | -0.1698 | 0.2594 | -0.6548 | 0.5131 | -0.6724 | 0.3327 |
|  | Time | -0.0176 | 0.0103 | -1.7067 | 0.0889 | -0.0378 | 0.0026 |
|  | WML change | 0.0336 | 0.0564 | 0.5947 | 0.5525 | -0.0758 | 0.1429 |
|  | Age | -0.0456 | 0.0565 | -0.8075 | 0.4200 | -0.1552 | 0.0639 |
|  | Sex: Male | 0.0898 | 0.1286 | 0.6983 | 0.4855 | -0.1594 | 0.3390 |
|  | Site: ED2 | 0.3676 | 0.2805 | 1.3105 | 0.1910 | -0.1759 | 0.9110 |
|  | Site: ED1 | -0.3914 | 0.2805 | -1.3955 | 0.1639 | -0.9348 | 0.1520 |
|  | Site: DUN | 0.0482 | 0.3108 | 0.1551 | 0.8769 | -0.5540 | 0.6504 |
|  | Site: GLA | 0.0971 | 0.2788 | 0.3483 | 0.7279 | -0.4430 | 0.6372 |
|  | DMT | 0.1701 | 0.1344 | 1.2650 | 0.2068 | -0.0904 | 0.4306 |
| NAWM superior frontal L | Intercept | 0.3614 | 0.2605 | 1.3871 | 0.1664 | -0.1434 | 0.8662 |
|  | Time*Age | -0.0378 | 0.0137 | -2.7515 | **0.0063** | -0.0647 | -0.0109 |
|  | Time | -0.0348 | 0.0137 | -2.5346 | **0.0117** | -0.0617 | -0.0079 |
|  | WML change | -0.0183 | 0.0565 | -0.3240 | 0.7461 | -0.1277 | 0.0911 |
|  | Age | 0.0161 | 0.0571 | 0.2817 | 0.7784 | -0.0946 | 0.1268 |
|  | Sex: Male | -0.0281 | 0.1279 | -0.2200 | 0.8260 | -0.2759 | 0.2197 |
|  | Site: ED2 | -0.0956 | 0.2815 | -0.3398 | 0.7343 | -0.6411 | 0.4498 |
|  | Site: ED1 | -0.7911 | 0.2823 | -2.8025 | **0.0054** | -1.3381 | -0.2442 |
|  | Site: DUN | -0.3175 | 0.3123 | -1.0166 | 0.3102 | -0.9227 | 0.2876 |
|  | Site: GLA | -0.3658 | 0.2799 | -1.3069 | 0.1922 | -0.9080 | 0.1765 |
|  | DMT | 0.0594 | 0.1354 | 0.4384 | 0.6614 | -0.2031 | 0.3218 |
| NAWM superior frontal R | Intercept | 0.5809 | 0.2547 | 2.2805 | 0.0233 | 0.0873 | 1.0744 |
|  | Time*Age | -0.0366 | 0.0131 | -2.7928 | **0.0055** | -0.0623 | -0.0109 |
|  | Time | -0.0272 | 0.0131 | -2.0744 | **0.0389** | -0.0528 | -0.0015 |
|  | WML change | -0.0515 | 0.0553 | -0.9311 | 0.3525 | -0.1585 | 0.0556 |
|  | Age | 0.0096 | 0.0558 | 0.1714 | 0.8640 | -0.0986 | 0.1178 |
|  | Sex: Male | -0.1983 | 0.1256 | -1.5787 | 0.1154 | -0.4417 | 0.0451 |
|  | Site: ED2 | -0.1308 | 0.2752 | -0.4755 | 0.6348 | -0.6641 | 0.4024 |
|  | Site: ED1 | -0.9919 | 0.2755 | -3.6010 | **0.0004** | -1.5256 | -0.4582 |
|  | Site: DUN | -0.6810 | 0.3052 | -2.2311 | **0.0264** | -1.2725 | -0.0896 |
|  | Site: GLA | -0.5850 | 0.2736 | -2.1380 | **0.0333** | -1.1152 | -0.0548 |
|  | DMT | 0.0571 | 0.1319 | 0.4331 | 0.6653 | -0.1984 | 0.3127 |
| GM bank ssts L | Intercept | -0.4457 | 0.2515 | -1.7718 | 0.0774 | -0.9331 | 0.0417 |
|  | Time | -0.0399 | 0.0091 | -4.3896 | **<0.0001** | -0.0577 | -0.0220 |
|  | WML change | 0.0482 | 0.0546 | 0.8828 | 0.3781 | -0.0576 | 0.1539 |
|  | Age | -0.2982 | 0.0548 | -5.4435 | **<0.0001** | -0.4044 | -0.1921 |
|  | Sex: Male | -0.2387 | 0.1235 | -1.9323 | 0.0543 | -0.4780 | 0.0007 |
|  | Site: ED2 | 0.5972 | 0.2718 | 2.1974 | **0.0287** | 0.0706 | 1.1238 |
|  | Site: ED1 | 0.6342 | 0.2725 | 2.3277 | **0.0206** | 0.1063 | 1.1622 |
|  | Site: DUN | 0.4872 | 0.3015 | 1.6160 | 0.1071 | -0.0970 | 1.0713 |
|  | Site: GLA | 0.3632 | 0.2701 | 1.3445 | 0.1798 | -0.1602 | 0.8866 |
|  | DMT | 0.0673 | 0.1310 | 0.5136 | 0.6079 | -0.1865 | 0.3210 |
| GM bank ssts R | Intercept | -0.1079 | 0.2509 | -0.4301 | 0.6674 | -0.5941 | 0.3782 |
|  | Time | -0.0338 | 0.0118 | -2.8521 | **0.0046** | -0.0570 | -0.0105 |
|  | WML change | -0.0225 | 0.0545 | -0.4134 | 0.6796 | -0.1282 | 0.0831 |
|  | Age | -0.2924 | 0.0547 | -5.3450 | **<0.0001** | -0.3984 | -0.1864 |
|  | Sex: Male | -0.3754 | 0.1233 | -3.0448 | **0.0025** | -0.6143 | -0.1365 |
|  | Site: ED2 | 0.4076 | 0.2712 | 1.5028 | 0.1339 | -0.1179 | 0.9330 |
|  | Site: ED1 | 0.3524 | 0.2716 | 1.2976 | 0.1954 | -0.1738 | 0.8787 |
|  | Site: DUN | 0.2620 | 0.3008 | 0.8709 | 0.3845 | -0.3208 | 0.8448 |
|  | Site: GLA | 0.1185 | 0.2696 | 0.4394 | 0.6607 | -0.4039 | 0.6408 |
|  | DMT | -0.0410 | 0.1301 | -0.3156 | 0.7525 | -0.2930 | 0.2110 |
| GM entorhinal L | Intercept | -0.4737 | 0.2733 | -1.7335 | 0.0840 | -1.0030 | 0.0557 |
|  | Time | -0.0066 | 0.0186 | -0.3544 | 0.7233 | -0.0430 | 0.0298 |
|  | WML change | 0.0712 | 0.0575 | 1.2379 | 0.2167 | -0.0402 | 0.1827 |
|  | Age | 0.0137 | 0.0579 | 0.2358 | 0.8137 | -0.0985 | 0.1259 |
|  | Sex: Male | 0.0232 | 0.1300 | 0.1783 | 0.8586 | -0.2287 | 0.2751 |
|  | Site: ED2 | 0.4780 | 0.2923 | 1.6350 | 0.1031 | -0.0884 | 1.0443 |
|  | Site: ED1 | 0.1091 | 0.2922 | 0.3735 | 0.7091 | -0.4569 | 0.6752 |
|  | Site: DUN | 0.4338 | 0.3254 | 1.3329 | 0.1836 | -0.1967 | 1.0642 |
|  | Site: GLA | 0.4387 | 0.2903 | 1.5112 | 0.1318 | -0.1237 | 1.0011 |
|  | DMT | 0.1658 | 0.1380 | 1.2014 | 0.2305 | -0.1016 | 0.4333 |
| GM entorhinal R | Intercept | -0.4801 | 0.2537 | -1.8921 | 0.0594 | -0.9716 | 0.0115 |
|  | Time | -0.0252 | 0.0184 | -1.3689 | 0.1720 | -0.0612 | 0.0109 |
|  | WML change | 0.0007 | 0.0554 | 0.0119 | 0.9905 | -0.1066 | 0.1079 |
|  | Age | -0.1773 | 0.0556 | -3.1910 | **0.0016** | -0.2849 | -0.0696 |
|  | Sex: Male | 0.0890 | 0.1253 | 0.7103 | 0.4781 | -0.1537 | 0.3317 |
|  | Site: ED2 | 0.9055 | 0.2742 | 3.3018 | **0.0011** | 0.3742 | 1.4368 |
|  | Site: ED1 | 0.3216 | 0.2743 | 1.1723 | 0.2420 | -0.2099 | 0.8530 |
|  | Site: DUN | 0.3293 | 0.3051 | 1.0793 | 0.2813 | -0.2618 | 0.9204 |
|  | Site: GLA | 0.6940 | 0.2726 | 2.5458 | **0.0114** | 0.1659 | 1.2221 |
|  | DMT | -0.1501 | 0.1316 | -1.1406 | 0.2549 | -0.4050 | 0.1048 |
| GM fusiform L | Intercept | -0.4028 | 0.2526 | -1.5945 | 0.1119 | -0.8923 | 0.0867 |
|  | Time | -0.0512 | 0.0128 | -4.0073 | **0.0001** | -0.0763 | -0.0261 |
|  | WML change | -0.0470 | 0.0552 | -0.8513 | 0.3953 | -0.1539 | 0.0599 |
|  | Age | -0.2521 | 0.0554 | -4.5497 | **<0.0001** | -0.3595 | -0.1448 |
|  | Sex: Male | -0.1473 | 0.1245 | -1.1829 | 0.2378 | -0.3885 | 0.0939 |
|  | Site: ED2 | 0.8997 | 0.2733 | 3.2920 | **0.0011** | 0.3702 | 1.4293 |
|  | Site: ED1 | 0.4236 | 0.2734 | 1.5491 | 0.1224 | -0.1062 | 0.9534 |
|  | Site: DUN | 0.6730 | 0.3032 | 2.2193 | **0.0272** | 0.0855 | 1.2605 |
|  | Site: GLA | 0.6414 | 0.2720 | 2.3578 | **0.0190** | 0.1143 | 1.1685 |
|  | DMT | -0.2117 | 0.1311 | -1.6155 | 0.1073 | -0.4657 | 0.0422 |
| GM fusiform R | Intercept | -0.6283 | 0.2476 | -2.5373 | **0.0117** | -1.1081 | -0.1485 |
|  | Time | -0.0307 | 0.0113 | -2.7082 | **0.0071** | -0.0529 | -0.0084 |
|  | WML change | -0.1130 | 0.0539 | -2.0949 | **0.0370** | -0.2175 | -0.0085 |
|  | Age | -0.2556 | 0.0542 | -4.7134 | **<0.0001** | -0.3606 | -0.1505 |
|  | Sex: Male | 0.0381 | 0.1219 | 0.3127 | 0.7548 | -0.1981 | 0.2744 |
|  | Site: ED2 | 1.0830 | 0.2679 | 4.0425 | **0.0001** | 0.5639 | 1.6020 |
|  | Site: ED1 | 0.5457 | 0.2680 | 2.0361 | **0.0426** | 0.0264 | 1.0649 |
|  | Site: DUN | 0.6617 | 0.2972 | 2.2261 | **0.0267** | 0.0858 | 1.2375 |
|  | Site: GLA | 0.8557 | 0.2665 | 3.2105 | **0.0015** | 0.3393 | 1.3721 |
|  | DMT | -0.1871 | 0.1285 | -1.4561 | 0.1464 | -0.4361 | 0.0619 |
| GM inferior temporal L | Intercept | -0.0297 | 0.2485 | -0.1197 | 0.9048 | -0.5112 | 0.4517 |
|  | Time | -0.0447 | 0.0127 | -3.5313 | **0.0005** | -0.0695 | -0.0199 |
|  | WML change | -0.0718 | 0.0540 | -1.3285 | 0.1850 | -0.1765 | 0.0329 |
|  | Age | -0.2640 | 0.0543 | -4.8637 | **<0.0001** | -0.3691 | -0.1588 |
|  | Sex: Male | -0.2327 | 0.1222 | -1.9042 | 0.0578 | -0.4695 | 0.0041 |
|  | Site: ED2 | 0.3953 | 0.2688 | 1.4707 | 0.1424 | -0.1254 | 0.9160 |
|  | Site: ED1 | -0.2559 | 0.2689 | -0.9517 | 0.3420 | -0.7768 | 0.2650 |
|  | Site: DUN | 0.2578 | 0.2989 | 0.8623 | 0.3892 | -0.3214 | 0.8370 |
|  | Site: GLA | 0.3123 | 0.2670 | 1.1695 | 0.2431 | -0.2051 | 0.8297 |
|  | DMT | -0.0887 | 0.1289 | -0.6884 | 0.4917 | -0.3384 | 0.1610 |
| GM inferior temporal R | Intercept | -0.4368 | 0.2542 | -1.7186 | 0.0867 | -0.9292 | 0.0557 |
|  | Time | -0.0278 | 0.0108 | -2.5747 | **0.0105** | -0.0490 | -0.0066 |
|  | WML change | 0.0576 | 0.0551 | 1.0471 | 0.2959 | -0.0490 | 0.1643 |
|  | Age | -0.2678 | 0.0553 | -4.8455 | **<0.0001** | -0.3748 | -0.1607 |
|  | Sex: Male | -0.1439 | 0.1248 | -1.1532 | 0.2497 | -0.3856 | 0.0979 |
|  | Site: ED2 | 0.7847 | 0.2747 | 2.8570 | **0.0046** | 0.2525 | 1.3169 |
|  | Site: ED1 | 0.3779 | 0.2749 | 1.3747 | 0.1702 | -0.1548 | 0.9107 |
|  | Site: DUN | 0.4674 | 0.3047 | 1.5341 | 0.1260 | -0.1229 | 1.0577 |
|  | Site: GLA | 0.6334 | 0.2730 | 2.3204 | **0.0210** | 0.1045 | 1.1624 |
|  | DMT | -0.0993 | 0.1316 | -0.7542 | 0.4513 | -0.3543 | 0.1558 |
| GM middle temporal L | Intercept | -0.3536 | 0.2362 | -1.4971 | 0.1354 | -0.8113 | 0.1041 |
|  | Time | -0.0535 | 0.0099 | -5.3785 | **<0.0001** | -0.0730 | -0.0340 |
|  | WML change | 0.0355 | 0.0512 | 0.6932 | 0.4887 | -0.0637 | 0.1346 |
|  | Age | -0.4040 | 0.0514 | -7.8664 | **<0.0001** | -0.5035 | -0.3045 |
|  | Sex: Male | -0.2310 | 0.1160 | -1.9918 | **0.0473** | -0.4556 | -0.0063 |
|  | Site: ED2 | 0.7159 | 0.2553 | 2.8042 | **0.0054** | 0.2212 | 1.2105 |
|  | Site: ED1 | 0.3113 | 0.2555 | 1.2181 | 0.2241 | -0.1839 | 0.8064 |
|  | Site: DUN | 0.0903 | 0.2832 | 0.3189 | 0.7500 | -0.4584 | 0.6390 |
|  | Site: GLA | 0.3212 | 0.2537 | 1.2660 | 0.2065 | -0.1704 | 0.8128 |
|  | DMT | 0.0950 | 0.1223 | 0.7767 | 0.4379 | -0.1420 | 0.3321 |
| GM middle temporal R | Intercept | -0.1526 | 0.2404 | -0.6348 | 0.5260 | -0.6185 | 0.3132 |
|  | Time | -0.0528 | 0.0114 | -4.6230 | **<0.0001** | -0.0752 | -0.0304 |
|  | WML change | 0.0228 | 0.0521 | 0.4375 | 0.6621 | -0.0781 | 0.1237 |
|  | Age | -0.4019 | 0.0524 | -7.6752 | **<0.0001** | -0.5033 | -0.3004 |
|  | Sex: Male | -0.1971 | 0.1181 | -1.6687 | 0.0962 | -0.4259 | 0.0318 |
|  | Site: ED2 | 0.4545 | 0.2598 | 1.7493 | 0.0813 | -0.0489 | 0.9580 |
|  | Site: ED1 | 0.1284 | 0.2601 | 0.4936 | 0.6219 | -0.3756 | 0.6323 |
|  | Site: DUN | -0.0249 | 0.2891 | -0.0861 | 0.9315 | -0.5851 | 0.5354 |
|  | Site: GLA | 0.1069 | 0.2583 | 0.4141 | 0.6791 | -0.3935 | 0.6073 |
|  | DMT | 0.0766 | 0.1245 | 0.6150 | 0.5390 | -0.1647 | 0.3178 |
| GM parahippocampal L | Intercept | -0.0845 | 0.2556 | -0.3306 | 0.7412 | -0.5798 | 0.4108 |
|  | Time | -0.0711 | 0.0127 | -5.5987 | **<0.0001** | -0.0961 | -0.0462 |
|  | WML change | -0.0608 | 0.0558 | -1.0901 | 0.2766 | -0.1688 | 0.0472 |
|  | Age | -0.1654 | 0.0561 | -2.9504 | **0.0034** | -0.2741 | -0.0568 |
|  | Sex: Male | -0.5016 | 0.1260 | -3.9820 | **0.0001** | -0.7456 | -0.2575 |
|  | Site: ED2 | 0.2336 | 0.2765 | 0.8447 | 0.3990 | -0.3022 | 0.7694 |
|  | Site: ED1 | 0.1631 | 0.2766 | 0.5895 | 0.5560 | -0.3729 | 0.6991 |
|  | Site: DUN | -0.0161 | 0.3068 | -0.0525 | 0.9581 | -0.6106 | 0.5783 |
|  | Site: GLA | 0.2831 | 0.2752 | 1.0286 | 0.3045 | -0.2501 | 0.8163 |
|  | DMT | 0.0621 | 0.1327 | 0.4684 | 0.6398 | -0.1949 | 0.3192 |
| GM parahippocampal R | Intercept | 0.0165 | 0.2521 | 0.0656 | 0.9477 | -0.4720 | 0.5051 |
|  | Time | -0.0343 | 0.0130 | -2.6424 | **0.0086** | -0.0598 | -0.0088 |
|  | WML change | 0.0129 | 0.0546 | 0.2370 | 0.8128 | -0.0929 | 0.1188 |
|  | Age | -0.2350 | 0.0548 | -4.2878 | **<0.0001** | -0.3412 | -0.1288 |
|  | Sex: Male | -0.5014 | 0.1238 | -4.0515 | **0.0001** | -0.7412 | -0.2616 |
|  | Site: ED2 | 0.3089 | 0.2725 | 1.1336 | 0.2579 | -0.2191 | 0.8368 |
|  | Site: ED1 | 0.1326 | 0.2727 | 0.4861 | 0.6273 | -0.3959 | 0.6610 |
|  | Site: DUN | -0.1237 | 0.3022 | -0.4094 | 0.6825 | -0.7093 | 0.4619 |
|  | Site: GLA | 0.2517 | 0.2708 | 0.9297 | 0.3533 | -0.2730 | 0.7764 |
|  | DMT | -0.0805 | 0.1306 | -0.6168 | 0.5378 | -0.3335 | 0.1725 |
| GM superior temporal L | Intercept | -0.1435 | 0.2396 | -0.5988 | 0.5497 | -0.6078 | 0.3208 |
|  | Time | -0.0616 | 0.0123 | -5.0033 | **<0.0001** | -0.0858 | -0.0375 |
|  | WML change | 0.0741 | 0.0520 | 1.4243 | 0.1554 | -0.0267 | 0.1748 |
|  | Age | -0.4310 | 0.0522 | -8.2644 | **<0.0001** | -0.5321 | -0.3300 |
|  | Sex: Male | -0.1499 | 0.1177 | -1.2737 | 0.2037 | -0.3779 | 0.0781 |
|  | Site: ED2 | 0.5504 | 0.2590 | 2.1251 | **0.0344** | 0.0486 | 1.0522 |
|  | Site: ED1 | 0.4353 | 0.2594 | 1.6784 | 0.0943 | -0.0672 | 0.9378 |
|  | Site: DUN | 0.2690 | 0.2873 | 0.9363 | 0.3499 | -0.2876 | 0.8256 |
|  | Site: GLA | 0.4024 | 0.2574 | 1.5634 | 0.1190 | -0.0963 | 0.9012 |
|  | DMT | -0.2696 | 0.1242 | -2.1702 | **0.0308** | -0.5102 | -0.0289 |
| GM superior temporal R | Intercept | 0.1406 | 0.2377 | 0.5916 | 0.5546 | -0.3199 | 0.6010 |
|  | Time | -0.0524 | 0.0141 | -3.7128 | **0.0002** | -0.0801 | -0.0247 |
|  | WML change | 0.0257 | 0.0517 | 0.4972 | 0.6194 | -0.0745 | 0.1259 |
|  | Age | -0.4061 | 0.0519 | -7.8213 | **<0.0001** | -0.5067 | -0.3055 |
|  | Sex: Male | -0.4807 | 0.1168 | -4.1137 | **0.0001** | -0.7070 | -0.2543 |
|  | Site: ED2 | 0.2619 | 0.2568 | 1.0195 | 0.3088 | -0.2358 | 0.7595 |
|  | Site: ED1 | 0.0066 | 0.2572 | 0.0257 | 0.9795 | -0.4917 | 0.5050 |
|  | Site: DUN | 0.0739 | 0.2860 | 0.2583 | 0.7964 | -0.4802 | 0.6279 |
|  | Site: GLA | -0.0197 | 0.2553 | -0.0772 | 0.9385 | -0.5144 | 0.4750 |
|  | DMT | -0.0757 | 0.1232 | -0.6148 | 0.5392 | -0.3144 | 0.1630 |
| GM temporal pole L | Intercept | 0.6903 | 0.2581 | 2.6744 | **0.0079** | 0.1902 | 1.1905 |
|  | Time | -0.0721 | 0.0222 | -3.2482 | **0.0013** | -0.1156 | -0.0285 |
|  | WML change | 0.0477 | 0.0561 | 0.8505 | 0.3957 | -0.0609 | 0.1563 |
|  | Age | -0.0788 | 0.0563 | -1.4005 | 0.1624 | -0.1879 | 0.0302 |
|  | Sex: Male | -0.3349 | 0.1267 | -2.6431 | **0.0086** | -0.5805 | -0.0894 |
|  | Site: ED2 | -0.3156 | 0.2794 | -1.1297 | 0.2595 | -0.8569 | 0.2257 |
|  | Site: ED1 | -0.5332 | 0.2790 | -1.9111 | 0.0569 | -1.0738 | 0.0074 |
|  | Site: DUN | -0.6809 | 0.3092 | -2.2020 | **0.0284** | -1.2801 | -0.0818 |
|  | Site: GLA | -0.5524 | 0.2771 | -1.9935 | **0.0471** | -1.0893 | -0.0155 |
|  | DMT | -0.1188 | 0.1344 | -0.8844 | 0.3772 | -0.3791 | 0.1415 |
| GM temporal pole R | Intercept | 0.0216 | 0.2616 | 0.0824 | 0.9344 | -0.4852 | 0.5284 |
|  | Time | -0.0210 | 0.0179 | -1.1683 | 0.2436 | -0.0562 | 0.0143 |
|  | WML change | -0.0956 | 0.0568 | -1.6819 | 0.0936 | -0.2058 | 0.0145 |
|  | Age | -0.0382 | 0.0572 | -0.6684 | 0.5044 | -0.1490 | 0.0726 |
|  | Sex: Male | -0.3721 | 0.1287 | -2.8913 | **0.0041** | -0.6215 | -0.1227 |
|  | Site: ED2 | 0.1190 | 0.2827 | 0.4211 | 0.6740 | -0.4287 | 0.6667 |
|  | Site: ED1 | 0.2400 | 0.2829 | 0.8481 | 0.3971 | -0.3083 | 0.7882 |
|  | Site: DUN | 0.0039 | 0.3138 | 0.0124 | 0.9901 | -0.6041 | 0.6119 |
|  | Site: GLA | 0.2309 | 0.2814 | 0.8208 | 0.4124 | -0.3142 | 0.7761 |
|  | DMT | -0.1260 | 0.1355 | -0.9298 | 0.3532 | -0.3886 | 0.1366 |
| GM transverse temporal L | Intercept | 0.0169 | 0.2711 | 0.0622 | 0.9504 | -0.5084 | 0.5422 |
|  | Time | -0.0603 | 0.0120 | -5.0434 | **<0.0001** | -0.0837 | -0.0368 |
|  | WML change | 0.0725 | 0.0566 | 1.2820 | 0.2008 | -0.0371 | 0.1821 |
|  | Age | -0.2113 | 0.0570 | -3.7095 | **0.0002** | -0.3217 | -0.1009 |
|  | Sex: Male | -0.2238 | 0.1282 | -1.7464 | 0.0817 | -0.4722 | 0.0245 |
|  | Site: ED2 | 0.1613 | 0.2900 | 0.5561 | 0.5786 | -0.4006 | 0.7232 |
|  | Site: ED1 | 0.2502 | 0.2902 | 0.8622 | 0.3893 | -0.3121 | 0.8126 |
|  | Site: DUN | 0.2840 | 0.3192 | 0.8896 | 0.3744 | -0.3345 | 0.9024 |
|  | Site: GLA | 0.0440 | 0.2877 | 0.1529 | 0.8786 | -0.5135 | 0.6015 |
|  | DMT | -0.1005 | 0.1352 | -0.7431 | 0.4580 | -0.3626 | 0.1615 |
| GM transverse temporal R | Intercept | 0.2038 | 0.2500 | 0.8152 | 0.4156 | -0.2807 | 0.6883 |
|  | Time | -0.0593 | 0.0146 | -4.0557 | **0.0001** | -0.0880 | -0.0306 |
|  | WML change | 0.0530 | 0.0543 | 0.9764 | 0.3297 | -0.0522 | 0.1582 |
|  | Age | -0.2762 | 0.0545 | -5.0712 | **<0.0001** | -0.3817 | -0.1706 |
|  | Sex: Male | -0.4239 | 0.1228 | -3.4525 | **0.0006** | -0.6618 | -0.1860 |
|  | Site: ED2 | 0.1928 | 0.2702 | 0.7135 | 0.4761 | -0.3308 | 0.7163 |
|  | Site: ED1 | -0.0431 | 0.2705 | -0.1595 | 0.8734 | -0.5671 | 0.4809 |
|  | Site: DUN | 0.3187 | 0.2997 | 1.0634 | 0.2884 | -0.2620 | 0.8994 |
|  | Site: GLA | -0.1540 | 0.2686 | -0.5732 | 0.5669 | -0.6744 | 0.3665 |
|  | DMT | -0.1060 | 0.1295 | -0.8186 | 0.4137 | -0.3569 | 0.1449 |
| NAWM banks sts L | Intercept | -0.3826 | 0.2638 | -1.4504 | 0.1480 | -0.8939 | 0.1286 |
|  | Time | -0.0282 | 0.0062 | -4.5194 | **<0.0001** | -0.0404 | -0.0159 |
|  | WML change | 0.0179 | 0.0572 | 0.3130 | 0.7545 | -0.0929 | 0.1286 |
|  | Age | -0.0725 | 0.0574 | -1.2641 | 0.2071 | -0.1837 | 0.0386 |
|  | Sex: Male | -0.3634 | 0.1295 | -2.8059 | **0.0053** | -0.6144 | -0.1125 |
|  | Site: ED2 | 0.4939 | 0.2852 | 1.7320 | 0.0843 | -0.0586 | 1.0465 |
|  | Site: ED1 | 0.4219 | 0.2854 | 1.4782 | 0.1404 | -0.1311 | 0.9750 |
|  | Site: DUN | 0.5440 | 0.3163 | 1.7199 | 0.0865 | -0.0689 | 1.1569 |
|  | Site: GLA | 0.3271 | 0.2834 | 1.1540 | 0.2494 | -0.2221 | 0.8762 |
|  | DMT | 0.1248 | 0.1367 | 0.9134 | 0.3617 | -0.1400 | 0.3896 |
| NAWM banks sts R | Intercept | 0.2273 | 0.2631 | 0.8641 | 0.3882 | -0.2824 | 0.7371 |
|  | Time | -0.0356 | 0.0072 | -4.9273 | **<0.0001** | -0.0498 | -0.0214 |
|  | WML change | -0.0348 | 0.0570 | -0.6103 | 0.5421 | -0.1452 | 0.0757 |
|  | Age | -0.0563 | 0.0572 | -0.9846 | 0.3256 | -0.1672 | 0.0545 |
|  | Sex: Male | -0.4016 | 0.1292 | -3.1091 | **0.0021** | -0.6518 | -0.1513 |
|  | Site: ED2 | -0.1541 | 0.2843 | -0.5418 | 0.5883 | -0.7050 | 0.3969 |
|  | Site: ED1 | 0.1346 | 0.2846 | 0.4731 | 0.6365 | -0.4169 | 0.6862 |
|  | Site: DUN | -0.1594 | 0.3154 | -0.5054 | 0.6136 | -0.7706 | 0.4517 |
|  | Site: GLA | -0.2439 | 0.2826 | -0.8630 | 0.3888 | -0.7915 | 0.3037 |
|  | DMT | 0.0053 | 0.1363 | 0.0390 | 0.9689 | -0.2587 | 0.2694 |
| NAWM entorhinal L | Intercept | -0.4527 | 0.2660 | -1.7020 | 0.0898 | -0.9681 | 0.0627 |
|  | Time | -0.0444 | 0.0138 | -3.2247 | **0.0014** | -0.0714 | -0.0174 |
|  | WML change | 0.0423 | 0.0577 | 0.7324 | 0.4645 | -0.0695 | 0.1541 |
|  | Age | 0.0373 | 0.0579 | 0.6445 | 0.5198 | -0.0749 | 0.1495 |
|  | Sex: Male | -0.0479 | 0.1312 | -0.3652 | 0.7152 | -0.3021 | 0.2063 |
|  | Site: ED2 | 0.5304 | 0.2874 | 1.8455 | 0.0659 | -0.0265 | 1.0872 |
|  | Site: ED1 | 0.3362 | 0.2877 | 1.1686 | 0.2435 | -0.2212 | 0.8936 |
|  | Site: DUN | 0.6810 | 0.3188 | 2.1362 | **0.0335** | 0.0633 | 1.2986 |
|  | Site: GLA | 0.3949 | 0.2858 | 1.3820 | 0.1680 | -0.1588 | 0.9486 |
|  | DMT | 0.0854 | 0.1377 | 0.6203 | 0.5355 | -0.1814 | 0.3523 |
| NAWM entorhinal R | Intercept | -0.0918 | 0.2629 | -0.3493 | 0.7271 | -0.6013 | 0.4176 |
|  | Time | -0.0402 | 0.0166 | -2.4201 | **0.0161** | -0.0728 | -0.0076 |
|  | WML change | -0.0293 | 0.0570 | -0.5139 | 0.6077 | -0.1397 | 0.0812 |
|  | Age | -0.0541 | 0.0573 | -0.9450 | 0.3454 | -0.1651 | 0.0568 |
|  | Sex: Male | -0.0349 | 0.1291 | -0.2708 | 0.7868 | -0.2850 | 0.2151 |
|  | Site: ED2 | 0.4363 | 0.2840 | 1.5363 | 0.1255 | -0.1140 | 0.9867 |
|  | Site: ED1 | -0.1022 | 0.2845 | -0.3593 | 0.7196 | -0.6535 | 0.4491 |
|  | Site: DUN | 0.0966 | 0.3151 | 0.3066 | 0.7594 | -0.5139 | 0.7070 |
|  | Site: GLA | 0.0279 | 0.2823 | 0.0987 | 0.9214 | -0.5191 | 0.5748 |
|  | DMT | 0.0333 | 0.1364 | 0.2445 | 0.8070 | -0.2309 | 0.2975 |
| NAWM fusiform L | Intercept | -0.1005 | 0.2651 | -0.3789 | 0.7050 | -0.6141 | 0.4132 |
|  | Time | -0.0925 | 0.0103 | -9.0060 | **<0.0001** | -0.1126 | -0.0723 |
|  | WML change | -0.0975 | 0.0578 | -1.6875 | 0.0925 | -0.2094 | 0.0144 |
|  | Age | -0.0717 | 0.0581 | -1.2329 | 0.2186 | -0.1843 | 0.0410 |
|  | Sex: Male | -0.2409 | 0.1318 | -1.8278 | 0.0686 | -0.4963 | 0.0144 |
|  | Site: ED2 | 0.3265 | 0.2864 | 1.1402 | 0.2551 | -0.2283 | 0.8813 |
|  | Site: ED1 | 0.1257 | 0.2870 | 0.4380 | 0.6617 | -0.4304 | 0.6818 |
|  | Site: DUN | 0.1862 | 0.3193 | 0.5833 | 0.5602 | -0.4324 | 0.8049 |
|  | Site: GLA | 0.0925 | 0.2850 | 0.3245 | 0.7458 | -0.4598 | 0.6448 |
|  | DMT | 0.0626 | 0.1376 | 0.4549 | 0.6495 | -0.2040 | 0.3292 |
| NAWM fusiform R | Intercept | -0.1330 | 0.2650 | -0.5018 | 0.6162 | -0.6464 | 0.3805 |
|  | Time | -0.0832 | 0.0100 | -8.3457 | **<0.0001** | -0.1028 | -0.0636 |
|  | WML change | -0.0953 | 0.0576 | -1.6539 | 0.0992 | -0.2069 | 0.0163 |
|  | Age | -0.0707 | 0.0580 | -1.2198 | 0.2235 | -0.1830 | 0.0416 |
|  | Sex: Male | -0.1756 | 0.1304 | -1.3460 | 0.1793 | -0.4283 | 0.0772 |
|  | Site: ED2 | 0.3893 | 0.2865 | 1.3589 | 0.1752 | -0.1658 | 0.9444 |
|  | Site: ED1 | 0.1437 | 0.2868 | 0.5011 | 0.6166 | -0.4119 | 0.6994 |
|  | Site: DUN | 0.0373 | 0.3180 | 0.1171 | 0.9068 | -0.5790 | 0.6535 |
|  | Site: GLA | 0.2461 | 0.2852 | 0.8628 | 0.3889 | -0.3065 | 0.7986 |
|  | DMT | -0.0041 | 0.1374 | -0.0296 | 0.9764 | -0.2702 | 0.2621 |
| NAWM inferior temporal L | Intercept | 0.1085 | 0.2592 | 0.4187 | 0.6758 | -0.3937 | 0.6107 |
|  | Time | -0.0578 | 0.0099 | -5.8325 | **<0.0001** | -0.0773 | -0.0384 |
|  | WML change | 0.0126 | 0.0562 | 0.2233 | 0.8235 | -0.0964 | 0.1215 |
|  | Age | -0.0758 | 0.0564 | -1.3430 | 0.1803 | -0.1852 | 0.0336 |
|  | Sex: Male | -0.0550 | 0.1273 | -0.4324 | 0.6658 | -0.3016 | 0.1916 |
|  | Site: ED2 | 0.3170 | 0.2803 | 1.1309 | 0.2590 | -0.2261 | 0.8601 |
|  | Site: ED1 | -0.4543 | 0.2804 | -1.6202 | 0.1062 | -0.9976 | 0.0890 |
|  | Site: DUN | -0.2996 | 0.3107 | -0.9642 | 0.3357 | -0.9016 | 0.3024 |
|  | Site: GLA | -0.1838 | 0.2784 | -0.6602 | 0.5096 | -0.7232 | 0.3556 |
|  | DMT | 0.1002 | 0.1344 | 0.7457 | 0.4564 | -0.1602 | 0.3606 |
| NAWM inferior temporal R | Intercept | -0.1318 | 0.2644 | -0.4987 | 0.6183 | -0.6441 | 0.3804 |
|  | Time | -0.0490 | 0.0087 | -5.6095 | **<0.0001** | -0.0661 | -0.0318 |
|  | WML change | 0.0785 | 0.0573 | 1.3701 | 0.1717 | -0.0325 | 0.1894 |
|  | Age | -0.0773 | 0.0575 | -1.3455 | 0.1794 | -0.1887 | 0.0340 |
|  | Sex: Male | 0.0409 | 0.1298 | 0.3154 | 0.7527 | -0.2105 | 0.2924 |
|  | Site: ED2 | 0.4539 | 0.2857 | 1.5886 | 0.1132 | -0.0997 | 1.0075 |
|  | Site: ED1 | -0.0012 | 0.2860 | -0.0043 | 0.9966 | -0.5554 | 0.5529 |
|  | Site: DUN | 0.0010 | 0.3169 | 0.0032 | 0.9974 | -0.6131 | 0.6151 |
|  | Site: GLA | 0.0465 | 0.2840 | 0.1639 | 0.8699 | -0.5037 | 0.5968 |
|  | DMT | 0.0356 | 0.1369 | 0.2597 | 0.7953 | -0.2298 | 0.3009 |
| NAWM middle temporal L | Intercept | 0.2756 | 0.2666 | 1.0339 | 0.3020 | -0.2409 | 0.7921 |
|  | Time | -0.0456 | 0.0103 | -4.4399 | **<0.0001** | -0.0657 | -0.0254 |
|  | WML change | -0.0677 | 0.0581 | -1.1658 | 0.2446 | -0.1802 | 0.0448 |
|  | Age | -0.0927 | 0.0583 | -1.5893 | 0.1130 | -0.2057 | 0.0203 |
|  | Sex: Male | -0.2274 | 0.1316 | -1.7276 | 0.0851 | -0.4824 | 0.0276 |
|  | Site: ED2 | -0.0956 | 0.2887 | -0.3311 | 0.7408 | -0.6548 | 0.4637 |
|  | Site: ED1 | -0.2544 | 0.2884 | -0.8824 | 0.3783 | -0.8131 | 0.3042 |
|  | Site: DUN | -0.3414 | 0.3196 | -1.0685 | 0.2862 | -0.9605 | 0.2777 |
|  | Site: GLA | -0.1994 | 0.2865 | -0.6960 | 0.4870 | -0.7545 | 0.3557 |
|  | DMT | -0.0011 | 0.1387 | -0.0082 | 0.9934 | -0.2698 | 0.2675 |
| NAWM middle temporal R | Intercept | 0.2498 | 0.2658 | 0.9398 | 0.3480 | -0.2653 | 0.7649 |
|  | Time | -0.0522 | 0.0109 | -4.8055 | **<0.0001** | -0.0736 | -0.0309 |
|  | WML change | 0.0058 | 0.0576 | 0.1008 | 0.9198 | -0.1058 | 0.1174 |
|  | Age | -0.0574 | 0.0578 | -0.9939 | 0.3211 | -0.1694 | 0.0545 |
|  | Sex: Male | -0.2582 | 0.1305 | -1.9790 | **0.0487** | -0.5111 | -0.0054 |
|  | Site: ED2 | -0.0744 | 0.2873 | -0.2591 | 0.7958 | -0.6311 | 0.4822 |
|  | Site: ED1 | -0.3661 | 0.2876 | -1.2730 | 0.2040 | -0.9233 | 0.1911 |
|  | Site: DUN | -0.3242 | 0.3187 | -1.0174 | 0.3097 | -0.9417 | 0.2932 |
|  | Site: GLA | -0.3294 | 0.2855 | -1.1537 | 0.2495 | -0.8827 | 0.2238 |
|  | DMT | 0.1419 | 0.1377 | 1.0309 | 0.3034 | -0.1248 | 0.4087 |
| NAWM parahippocampal L | Intercept | 0.2849 | 0.2630 | 1.0835 | 0.2795 | -0.2246 | 0.7944 |
|  | Time | -0.0587 | 0.0190 | -3.0862 | **0.0022** | -0.0961 | -0.0214 |
|  | WML change | -0.0077 | 0.0574 | -0.1346 | 0.8930 | -0.1190 | 0.1035 |
|  | Age | 0.0896 | 0.0577 | 1.5530 | 0.1215 | -0.0222 | 0.2013 |
|  | Sex: Male | -0.3106 | 0.1295 | -2.3981 | **0.0171** | -0.5616 | -0.0597 |
|  | Site: ED2 | -0.1744 | 0.2844 | -0.6134 | 0.5401 | -0.7254 | 0.3765 |
|  | Site: ED1 | -0.2220 | 0.2845 | -0.7803 | 0.4358 | -0.7731 | 0.3292 |
|  | Site: DUN | -0.4697 | 0.3155 | -1.4887 | 0.1376 | -1.0809 | 0.1416 |
|  | Site: GLA | -0.1618 | 0.2831 | -0.5718 | 0.5679 | -0.7102 | 0.3865 |
|  | DMT | 0.0338 | 0.1364 | 0.2479 | 0.8044 | -0.2305 | 0.2981 |
| NAWM parahippocampal R | Intercept | 0.0048 | 0.2650 | 0.0183 | 0.9854 | -0.5086 | 0.5183 |
|  | Time | -0.0635 | 0.0134 | -4.7352 | **<0.0001** | -0.0899 | -0.0372 |
|  | WML change | -0.0946 | 0.0578 | -1.6353 | 0.1030 | -0.2067 | 0.0175 |
|  | Age | 0.0954 | 0.0581 | 1.6433 | 0.1014 | -0.0171 | 0.2079 |
|  | Sex: Male | -0.2127 | 0.1305 | -1.6299 | 0.1042 | -0.4655 | 0.0401 |
|  | Site: ED2 | 0.2126 | 0.2864 | 0.7423 | 0.4585 | -0.3424 | 0.7676 |
|  | Site: ED1 | -0.0528 | 0.2867 | -0.1843 | 0.8539 | -0.6084 | 0.5027 |
|  | Site: DUN | -0.1128 | 0.3180 | -0.3548 | 0.7230 | -0.7289 | 0.5033 |
|  | Site: GLA | 0.0203 | 0.2853 | 0.0711 | 0.9433 | -0.5324 | 0.5730 |
|  | DMT | 0.0648 | 0.1373 | 0.4719 | 0.6374 | -0.2013 | 0.3309 |
| NAWM superior temporal L | Intercept | 0.2205 | 0.2658 | 0.8295 | 0.4075 | -0.2946 | 0.7356 |
|  | Time | -0.0489 | 0.0104 | -4.6780 | **<0.0001** | -0.0694 | -0.0284 |
|  | WML change | 0.0263 | 0.0577 | 0.4554 | 0.6491 | -0.0855 | 0.1380 |
|  | Age | -0.0239 | 0.0579 | -0.4133 | 0.6797 | -0.1361 | 0.0882 |
|  | Sex: Male | 0.2982 | 0.1306 | 2.2833 | **0.0231** | 0.0451 | 0.5512 |
|  | Site: ED2 | -0.0332 | 0.2873 | -0.1157 | 0.9080 | -0.5899 | 0.5235 |
|  | Site: ED1 | -0.2714 | 0.2876 | -0.9438 | 0.3460 | -0.8287 | 0.2858 |
|  | Site: DUN | -0.2643 | 0.3187 | -0.8295 | 0.4075 | -0.8818 | 0.3531 |
|  | Site: GLA | -0.2359 | 0.2856 | -0.8258 | 0.4096 | -0.7893 | 0.3176 |
|  | DMT | -0.1086 | 0.1377 | -0.7886 | 0.4310 | -0.3754 | 0.1582 |
| NAWM superior temporal R | Intercept | 0.3832 | 0.2635 | 1.4546 | 0.1468 | -0.1272 | 0.8937 |
|  | Time | -0.0575 | 0.0128 | -4.4985 | **<0.0001** | -0.0825 | -0.0324 |
|  | WML change | -0.0084 | 0.0572 | -0.1466 | 0.8835 | -0.1192 | 0.1024 |
|  | Age | 0.0142 | 0.0575 | 0.2468 | 0.8052 | -0.0971 | 0.1255 |
|  | Sex: Male | -0.1900 | 0.1300 | -1.4616 | 0.1449 | -0.4418 | 0.0619 |
|  | Site: ED2 | -0.1662 | 0.2847 | -0.5837 | 0.5599 | -0.7177 | 0.3854 |
|  | Site: ED1 | -0.6099 | 0.2849 | -2.1405 | **0.0331** | -1.1620 | -0.0578 |
|  | Site: DUN | -0.3938 | 0.3168 | -1.2431 | 0.2148 | -1.0076 | 0.2200 |
|  | Site: GLA | -0.5890 | 0.2830 | -2.0810 | **0.0383** | -1.1374 | -0.0406 |
|  | DMT | 0.1925 | 0.1365 | 1.4104 | 0.1595 | -0.0720 | 0.4570 |
| NAWM temporal pole L | Intercept | 0.6374 | 0.2489 | 2.5612 | **0.0109** | 0.1552 | 1.1196 |
|  | Time | -0.0990 | 0.0311 | -3.1791 | **0.0016** | -0.1601 | -0.0379 |
|  | WML change | -0.0049 | 0.0541 | -0.0915 | 0.9271 | -0.1097 | 0.0998 |
|  | Age | 0.0382 | 0.0542 | 0.7050 | 0.4813 | -0.0668 | 0.1432 |
|  | Sex: Male | 0.1449 | 0.1221 | 1.1870 | 0.2362 | -0.0916 | 0.3814 |
|  | Site: ED2 | -0.1077 | 0.2685 | -0.4012 | 0.6886 | -0.6280 | 0.4126 |
|  | Site: ED1 | -0.6174 | 0.2689 | -2.2956 | **0.0224** | -1.1384 | -0.0963 |
|  | Site: DUN | -0.6118 | 0.2979 | -2.0539 | **0.0408** | -1.1890 | -0.0347 |
|  | Site: GLA | -0.3243 | 0.2670 | -1.2146 | 0.2255 | -0.8416 | 0.1930 |
|  | DMT | -0.3604 | 0.1288 | -2.7980 | **0.0055** | -0.6099 | -0.1108 |
| NAWM temporal pole R | Intercept | -0.1374 | 0.2557 | -0.5374 | 0.5914 | -0.6328 | 0.3580 |
|  | Time | -0.0731 | 0.0275 | -2.6628 | **0.0082** | -0.1270 | -0.0192 |
|  | WML change | 0.0775 | 0.0556 | 1.3935 | 0.1645 | -0.0302 | 0.1851 |
|  | Age | 0.0510 | 0.0557 | 0.9145 | 0.3612 | -0.0570 | 0.1589 |
|  | Sex: Male | 0.0447 | 0.1255 | 0.3563 | 0.7219 | -0.1985 | 0.2879 |
|  | Site: ED2 | 0.4572 | 0.2760 | 1.6565 | 0.0986 | -0.0775 | 0.9919 |
|  | Site: ED1 | 0.0417 | 0.2770 | 0.1505 | 0.8804 | -0.4949 | 0.5783 |
|  | Site: DUN | -0.2822 | 0.3072 | -0.9188 | 0.3590 | -0.8774 | 0.3129 |
|  | Site: GLA | 0.1804 | 0.2744 | 0.6574 | 0.5114 | -0.3512 | 0.7120 |
|  | DMT | 0.0122 | 0.1328 | 0.0921 | 0.9267 | -0.2451 | 0.2696 |
| NAWM transverse temporal L | Intercept | -0.1828 | 0.2640 | -0.6924 | 0.4892 | -0.6942 | 0.3286 |
|  | Time | -0.0539 | 0.0174 | -3.0900 | **0.0022** | -0.0881 | -0.0197 |
|  | WML change | 0.0040 | 0.0576 | 0.0690 | 0.9451 | -0.1076 | 0.1156 |
|  | Age | 0.0858 | 0.0576 | 1.4891 | 0.1375 | -0.0258 | 0.1975 |
|  | Sex: Male | -0.2465 | 0.1298 | -1.8989 | 0.0585 | -0.4979 | 0.0050 |
|  | Site: ED2 | 0.1971 | 0.2858 | 0.6898 | 0.4908 | -0.3565 | 0.7508 |
|  | Site: ED1 | 0.0279 | 0.2863 | 0.0976 | 0.9223 | -0.5268 | 0.5826 |
|  | Site: DUN | 0.0447 | 0.3187 | 0.1402 | 0.8886 | -0.5728 | 0.6621 |
|  | Site: GLA | 0.2342 | 0.2838 | 0.8253 | 0.4098 | -0.3156 | 0.7840 |
|  | DMT | 0.1680 | 0.1386 | 1.2120 | 0.2265 | -0.1005 | 0.4366 |
| NAWM transverse temporal R | Intercept | 0.3959 | 0.2647 | 1.4959 | 0.1357 | -0.1169 | 0.9088 |
|  | Time | -0.0691 | 0.0186 | -3.7128 | **0.0002** | -0.1057 | -0.0326 |
|  | WML change | -0.0206 | 0.0573 | -0.3601 | 0.7190 | -0.1317 | 0.0904 |
|  | Age | -0.0081 | 0.0575 | -0.1412 | 0.8878 | -0.1196 | 0.1033 |
|  | Sex: Male | -0.3279 | 0.1299 | -2.5244 | **0.0121** | -0.5797 | -0.0762 |
|  | Site: ED2 | -0.2812 | 0.2865 | -0.9814 | 0.3272 | -0.8364 | 0.2740 |
|  | Site: ED1 | -0.2546 | 0.2862 | -0.8894 | 0.3745 | -0.8091 | 0.3000 |
|  | Site: DUN | -0.1998 | 0.3172 | -0.6298 | 0.5293 | -0.8143 | 0.4148 |
|  | Site: GLA | -0.2439 | 0.2843 | -0.8580 | 0.3916 | -0.7946 | 0.3069 |
|  | DMT | -0.0563 | 0.1373 | -0.4102 | 0.6820 | -0.3224 | 0.2097 |
| GM inferior parietal L | Intercept | -0.2441 | 0.2516 | -0.9700 | 0.3328 | -0.7315 | 0.2434 |
|  | Time | -0.0544 | 0.0090 | -6.0162 | **<0.0001** | -0.0721 | -0.0366 |
|  | WML change | -0.0110 | 0.0545 | -0.2015 | 0.8404 | -0.1167 | 0.0947 |
|  | Age | -0.2155 | 0.0548 | -3.9335 | **0.0001** | -0.3217 | -0.1094 |
|  | Sex: Male | -0.4169 | 0.1241 | -3.3588 | **0.0009** | -0.6575 | -0.1764 |
|  | Site: ED2 | 0.6517 | 0.2717 | 2.3981 | **0.0171** | 0.1251 | 1.1782 |
|  | Site: ED1 | 0.1001 | 0.2724 | 0.3675 | 0.7135 | -0.4276 | 0.6278 |
|  | Site: DUN | 0.3914 | 0.3014 | 1.2986 | 0.1951 | -0.1926 | 0.9755 |
|  | Site: GLA | 0.1726 | 0.2701 | 0.6393 | 0.5231 | -0.3506 | 0.6959 |
|  | DMT | 0.1277 | 0.1304 | 0.9791 | 0.3283 | -0.1250 | 0.3805 |
| GM inferior parietal R | Intercept | -0.4623 | 0.2476 | -1.8670 | 0.0629 | -0.9422 | 0.0175 |
|  | Time | -0.0306 | 0.0102 | -2.9927 | **0.0030** | -0.0506 | -0.0105 |
|  | WML change | 0.0037 | 0.0536 | 0.0696 | 0.9446 | -0.1002 | 0.1077 |
|  | Age | -0.2664 | 0.0538 | -4.9487 | **<0.0001** | -0.3708 | -0.1621 |
|  | Sex: Male | -0.3100 | 0.1216 | -2.5497 | **0.0113** | -0.5455 | -0.0744 |
|  | Site: ED2 | 0.9727 | 0.2676 | 3.6345 | **0.0003** | 0.4541 | 1.4913 |
|  | Site: ED1 | 0.6836 | 0.2679 | 2.5518 | **0.0112** | 0.1645 | 1.2027 |
|  | Site: DUN | 0.3993 | 0.2969 | 1.3450 | 0.1796 | -0.1759 | 0.9745 |
|  | Site: GLA | 0.4900 | 0.2660 | 1.8422 | 0.0664 | -0.0254 | 1.0054 |
|  | DMT | -0.0771 | 0.1283 | -0.6009 | 0.5483 | -0.3256 | 0.1715 |
| GM isthmus cingulate L | Intercept | -0.0278 | 0.2585 | -0.1076 | 0.9144 | -0.5286 | 0.4730 |
|  | Time | -0.0371 | 0.0110 | -3.3613 | **0.0009** | -0.0588 | -0.0154 |
|  | WML change | -0.0788 | 0.0560 | -1.4068 | 0.1605 | -0.1874 | 0.0297 |
|  | Age | -0.1533 | 0.0563 | -2.7244 | **0.0068** | -0.2624 | -0.0443 |
|  | Sex: Male | 0.1501 | 0.1274 | 1.1775 | 0.2399 | -0.0969 | 0.3970 |
|  | Site: ED2 | 0.1437 | 0.2793 | 0.5144 | 0.6074 | -0.3975 | 0.6848 |
|  | Site: ED1 | -0.4138 | 0.2796 | -1.4803 | 0.1398 | -0.9555 | 0.1278 |
|  | Site: DUN | -0.1329 | 0.3098 | -0.4290 | 0.6682 | -0.7332 | 0.4673 |
|  | Site: GLA | -0.0800 | 0.2777 | -0.2883 | 0.7733 | -0.6181 | 0.4580 |
|  | DMT | 0.1626 | 0.1339 | 1.2144 | 0.2255 | -0.0968 | 0.4219 |
| GM isthmus cingulate R | Intercept | -0.0018 | 0.2564 | -0.0069 | 0.9945 | -0.4986 | 0.4951 |
|  | Time | -0.0270 | 0.0114 | -2.3631 | **0.0187** | -0.0495 | -0.0046 |
|  | WML change | -0.0669 | 0.0557 | -1.2016 | 0.2305 | -0.1748 | 0.0410 |
|  | Age | -0.1984 | 0.0559 | -3.5493 | **0.0004** | -0.3067 | -0.0901 |
|  | Sex: Male | -0.2152 | 0.1265 | -1.7015 | 0.0899 | -0.4603 | 0.0299 |
|  | Site: ED2 | 0.4138 | 0.2773 | 1.4925 | 0.1366 | -0.1234 | 0.9511 |
|  | Site: ED1 | -0.1067 | 0.2774 | -0.3846 | 0.7008 | -0.6441 | 0.4308 |
|  | Site: DUN | -0.0301 | 0.3074 | -0.0979 | 0.9221 | -0.6257 | 0.5655 |
|  | Site: GLA | 0.2946 | 0.2756 | 1.0690 | 0.2859 | -0.2393 | 0.8285 |
|  | DMT | -0.1480 | 0.1329 | -1.1133 | 0.2665 | -0.4056 | 0.1096 |
| GM postcentral L | Intercept | -0.1007 | 0.2457 | -0.4097 | 0.6823 | -0.5768 | 0.3754 |
|  | Time | -0.0294 | 0.0173 | -1.7008 | 0.0900 | -0.0634 | 0.0045 |
|  | WML change | 0.0092 | 0.0535 | 0.1722 | 0.8634 | -0.0945 | 0.1129 |
|  | Age | -0.2702 | 0.0539 | -5.0174 | **<0.0001** | -0.3746 | -0.1659 |
|  | Sex: Male | -0.3208 | 0.1215 | -2.6396 | **0.0087** | -0.5562 | -0.0853 |
|  | Site: ED2 | 0.6967 | 0.2659 | 2.6198 | **0.0092** | 0.1815 | 1.2120 |
|  | Site: ED1 | -0.0287 | 0.2658 | -0.1080 | 0.9141 | -0.5436 | 0.4862 |
|  | Site: DUN | 0.2146 | 0.2948 | 0.7279 | 0.4672 | -0.3565 | 0.7857 |
|  | Site: GLA | 0.2610 | 0.2644 | 0.9870 | 0.3244 | -0.2513 | 0.7733 |
|  | DMT | -0.1072 | 0.1279 | -0.8385 | 0.4024 | -0.3550 | 0.1405 |
| GM postcentral R | Intercept | -0.4917 | 0.2515 | -1.9552 | 0.0515 | -0.9789 | -0.0044 |
|  | Time | -0.0215 | 0.0179 | -1.2043 | 0.2294 | -0.0567 | 0.0136 |
|  | WML change | 0.0803 | 0.0546 | 1.4707 | 0.1424 | -0.0255 | 0.1861 |
|  | Age | -0.1893 | 0.0548 | -3.4579 | **0.0006** | -0.2954 | -0.0832 |
|  | Sex: Male | -0.1303 | 0.1235 | -1.0554 | 0.2921 | -0.3696 | 0.1089 |
|  | Site: ED2 | 1.0074 | 0.2723 | 3.6991 | **0.0003** | 0.4798 | 1.5351 |
|  | Site: ED1 | 0.3679 | 0.2720 | 1.3526 | 0.1772 | -0.1591 | 0.8948 |
|  | Site: DUN | 0.5341 | 0.3014 | 1.7721 | 0.0774 | -0.0498 | 1.1181 |
|  | Site: GLA | 0.4635 | 0.2701 | 1.7158 | 0.0872 | -0.0599 | 0.9868 |
|  | DMT | -0.0153 | 0.1307 | -0.1172 | 0.9068 | -0.2686 | 0.2379 |
| GM posterior cingulate L | Intercept | -0.0322 | 0.2520 | -0.1279 | 0.8983 | -0.5205 | 0.4561 |
|  | Time | -0.0511 | 0.0112 | -4.5630 | **<0.0001** | -0.0730 | -0.0291 |
|  | WML change | 0.0508 | 0.0548 | 0.9266 | 0.3548 | -0.0554 | 0.1569 |
|  | Age | -0.3100 | 0.0549 | -5.6430 | **<0.0001** | -0.4164 | -0.2036 |
|  | Sex: Male | -0.0828 | 0.1239 | -0.6686 | 0.5043 | -0.3228 | 0.1572 |
|  | Site: ED2 | 0.4907 | 0.2726 | 1.8003 | 0.0728 | -0.0374 | 1.0188 |
|  | Site: ED1 | 0.1326 | 0.2727 | 0.4861 | 0.6272 | -0.3958 | 0.6609 |
|  | Site: DUN | -0.0062 | 0.3022 | -0.0206 | 0.9836 | -0.5917 | 0.5792 |
|  | Site: GLA | 0.2442 | 0.2708 | 0.9016 | 0.3680 | -0.2805 | 0.7689 |
|  | DMT | -0.2118 | 0.1307 | -1.6207 | 0.1061 | -0.4649 | 0.0414 |
| GM posterior cingulate R | Intercept | 0.1136 | 0.2433 | 0.4671 | 0.6408 | -0.3577 | 0.5849 |
|  | Time | -0.0295 | 0.0122 | -2.4243 | **0.0159** | -0.0534 | -0.0056 |
|  | WML change | 0.0496 | 0.0531 | 0.9332 | 0.3515 | -0.0533 | 0.1525 |
|  | Age | -0.3459 | 0.0532 | -6.4996 | **<0.0001** | -0.4490 | -0.2428 |
|  | Sex: Male | -0.0759 | 0.1202 | -0.6313 | 0.5283 | -0.3087 | 0.1570 |
|  | Site: ED2 | 0.3407 | 0.2632 | 1.2943 | 0.1965 | -0.1693 | 0.8506 |
|  | Site: ED1 | -0.4104 | 0.2632 | -1.5592 | 0.1200 | -0.9203 | 0.0995 |
|  | Site: DUN | 0.4071 | 0.2928 | 1.3903 | 0.1655 | -0.1602 | 0.9744 |
|  | Site: GLA | -0.0154 | 0.2615 | -0.0587 | 0.9532 | -0.5220 | 0.4913 |
|  | DMT | -0.1300 | 0.1262 | -1.0297 | 0.3040 | -0.3745 | 0.1146 |
| GM precuneus L | Intercept | 0.2367 | 0.2520 | 0.9393 | 0.3483 | -0.2516 | 0.7249 |
|  | Time | -0.0583 | 0.0136 | -4.3009 | **<0.0001** | -0.0849 | -0.0317 |
|  | WML change | 0.0336 | 0.0546 | 0.6149 | 0.5391 | -0.0722 | 0.1393 |
|  | Age | -0.2828 | 0.0548 | -5.1621 | **<0.0001** | -0.3889 | -0.1766 |
|  | Sex: Male | -0.3002 | 0.1237 | -2.4269 | **0.0158** | -0.5398 | -0.0605 |
|  | Site: ED2 | 0.1481 | 0.2723 | 0.5441 | 0.5868 | -0.3794 | 0.6757 |
|  | Site: ED1 | -0.2451 | 0.2725 | -0.8992 | 0.3692 | -0.7732 | 0.2830 |
|  | Site: DUN | -0.3544 | 0.3020 | -1.1734 | 0.2415 | -0.9396 | 0.2308 |
|  | Site: GLA | -0.1183 | 0.2706 | -0.4372 | 0.6622 | -0.6427 | 0.4060 |
|  | DMT | -0.0344 | 0.1305 | -0.2636 | 0.7923 | -0.2872 | 0.2184 |
| GM precuneus R | Intercept | -0.0841 | 0.2536 | -0.3317 | 0.7403 | -0.5755 | 0.4072 |
|  | Time | -0.0342 | 0.0140 | -2.4385 | **0.0153** | -0.0618 | -0.0067 |
|  | WML change | -0.0371 | 0.0550 | -0.6751 | 0.5001 | -0.1437 | 0.0695 |
|  | Age | -0.2096 | 0.0552 | -3.7972 | **0.0002** | -0.3166 | -0.1026 |
|  | Sex: Male | -0.1715 | 0.1245 | -1.3775 | 0.1694 | -0.4128 | 0.0697 |
|  | Site: ED2 | 0.5628 | 0.2740 | 2.0538 | **0.0408** | 0.0319 | 1.0938 |
|  | Site: ED1 | 0.0602 | 0.2745 | 0.2193 | 0.8266 | -0.4716 | 0.5920 |
|  | Site: DUN | -0.1834 | 0.3040 | -0.6034 | 0.5467 | -0.7724 | 0.4056 |
|  | Site: GLA | 0.3142 | 0.2724 | 1.1535 | 0.2496 | -0.2136 | 0.8420 |
|  | DMT | -0.1341 | 0.1314 | -1.0200 | 0.3085 | -0.3887 | 0.1206 |
| GM superior parietal L | Intercept | -0.2215 | 0.2509 | -0.8830 | 0.3779 | -0.7077 | 0.2646 |
|  | Time | -0.0525 | 0.0151 | -3.4701 | **0.0006** | -0.0822 | -0.0228 |
|  | WML change | 0.0365 | 0.0543 | 0.6719 | 0.5022 | -0.0688 | 0.1418 |
|  | Age | -0.1867 | 0.0545 | -3.4228 | **0.0007** | -0.2924 | -0.0810 |
|  | Sex: Male | -0.4937 | 0.1231 | -4.0094 | **0.0001** | -0.7323 | -0.2551 |
|  | Site: ED2 | 0.5808 | 0.2711 | 2.1426 | **0.0329** | 0.0556 | 1.1061 |
|  | Site: ED1 | 0.1143 | 0.2714 | 0.4213 | 0.6738 | -0.4115 | 0.6401 |
|  | Site: DUN | 0.1113 | 0.3007 | 0.3701 | 0.7115 | -0.4713 | 0.6939 |
|  | Site: GLA | 0.4209 | 0.2694 | 1.5621 | 0.1193 | -0.1012 | 0.9429 |
|  | DMT | 0.0524 | 0.1299 | 0.4032 | 0.6871 | -0.1994 | 0.3041 |
| GM superior parietal R | Intercept | -0.3277 | 0.2487 | -1.3177 | 0.1886 | -0.8094 | 0.1541 |
|  | Time | -0.0268 | 0.0160 | -1.6734 | 0.0953 | -0.0582 | 0.0046 |
|  | WML change | 0.0259 | 0.0539 | 0.4802 | 0.6315 | -0.0786 | 0.1304 |
|  | Age | -0.1874 | 0.0541 | -3.4637 | **0.0006** | -0.2923 | -0.0826 |
|  | Sex: Male | -0.4521 | 0.1227 | -3.6852 | **0.0003** | -0.6898 | -0.2144 |
|  | Site: ED2 | 0.8308 | 0.2689 | 3.0901 | **0.0022** | 0.3099 | 1.3517 |
|  | Site: ED1 | 0.3232 | 0.2688 | 1.2024 | 0.2301 | -0.1976 | 0.8441 |
|  | Site: DUN | 0.2564 | 0.2979 | 0.8609 | 0.3900 | -0.3207 | 0.8336 |
|  | Site: GLA | 0.7183 | 0.2669 | 2.6911 | **0.0075** | 0.2011 | 1.2355 |
|  | DMT | -0.1550 | 0.1288 | -1.2032 | 0.2298 | -0.4047 | 0.0946 |
| GM supramarginal L | Intercept | 0.0626 | 0.2545 | 0.2459 | 0.8059 | -0.4306 | 0.5558 |
|  | Time | -0.0454 | 0.0113 | -4.0016 | **0.0001** | -0.0676 | -0.0231 |
|  | WML change | 0.0314 | 0.0552 | 0.5690 | 0.5697 | -0.0755 | 0.1383 |
|  | Age | -0.2928 | 0.0554 | -5.2834 | **<0.0001** | -0.4002 | -0.1854 |
|  | Sex: Male | -0.0682 | 0.1256 | -0.5429 | 0.5876 | -0.3115 | 0.1751 |
|  | Site: ED2 | 0.1312 | 0.2749 | 0.4772 | 0.6336 | -0.4015 | 0.6638 |
|  | Site: ED1 | -0.0182 | 0.2755 | -0.0660 | 0.9474 | -0.5520 | 0.5157 |
|  | Site: DUN | -0.3957 | 0.3049 | -1.2979 | 0.1953 | -0.9865 | 0.1951 |
|  | Site: GLA | -0.0709 | 0.2732 | -0.2594 | 0.7955 | -0.6002 | 0.4585 |
|  | DMT | 0.0293 | 0.1320 | 0.2218 | 0.8246 | -0.2264 | 0.2849 |
| GM supramarginal R | Intercept | -0.2251 | 0.2503 | -0.8995 | 0.3691 | -0.7101 | 0.2598 |
|  | Time | -0.0383 | 0.0112 | -3.4154 | **0.0007** | -0.0603 | -0.0163 |
|  | WML change | 0.0200 | 0.0544 | 0.3684 | 0.7128 | -0.0854 | 0.1255 |
|  | Age | -0.3018 | 0.0546 | -5.5275 | **<0.0001** | -0.4076 | -0.1960 |
|  | Sex: Male | -0.0504 | 0.1230 | -0.4098 | 0.6822 | -0.2887 | 0.1879 |
|  | Site: ED2 | 0.5956 | 0.2705 | 2.2017 | **0.0284** | 0.0715 | 1.1197 |
|  | Site: ED1 | 0.0105 | 0.2709 | 0.0388 | 0.9691 | -0.5144 | 0.5354 |
|  | Site: DUN | 0.2700 | 0.3000 | 0.9000 | 0.3689 | -0.3113 | 0.8514 |
|  | Site: GLA | 0.1546 | 0.2689 | 0.5747 | 0.5659 | -0.3665 | 0.6756 |
|  | DMT | 0.0418 | 0.1297 | 0.3222 | 0.7476 | -0.2095 | 0.2931 |
| NAWM inferior parietal L | Intercept | -0.0833 | 0.2631 | -0.3166 | 0.7518 | -0.5931 | 0.4265 |
|  | Time | -0.0560 | 0.0080 | -7.0107 | **<0.0001** | -0.0716 | -0.0403 |
|  | WML change | -0.0325 | 0.0570 | -0.5707 | 0.5686 | -0.1430 | 0.0779 |
|  | Age | 0.0101 | 0.0572 | 0.1772 | 0.8594 | -0.1007 | 0.1210 |
|  | Sex: Male | -0.2222 | 0.1292 | -1.7199 | 0.0865 | -0.4724 | 0.0281 |
|  | Site: ED2 | 0.3445 | 0.2844 | 1.2115 | 0.2266 | -0.2065 | 0.8955 |
|  | Site: ED1 | -0.1492 | 0.2846 | -0.5241 | 0.6006 | -0.7007 | 0.4024 |
|  | Site: DUN | 0.0725 | 0.3154 | 0.2298 | 0.8184 | -0.5387 | 0.6836 |
|  | Site: GLA | -0.1548 | 0.2826 | -0.5477 | 0.5843 | -0.7024 | 0.3928 |
|  | DMT | 0.2380 | 0.1363 | 1.7466 | 0.0817 | -0.0260 | 0.5021 |
| NAWM inferior parietal R | Intercept | -0.1768 | 0.2672 | -0.6617 | 0.5087 | -0.6946 | 0.3409 |
|  | Time | -0.0392 | 0.0078 | -5.0385 | **<0.0001** | -0.0545 | -0.0239 |
|  | WML change | 0.0119 | 0.0580 | 0.2058 | 0.8371 | -0.1004 | 0.1243 |
|  | Age | -0.0076 | 0.0582 | -0.1298 | 0.8968 | -0.1204 | 0.1053 |
|  | Sex: Male | -0.0157 | 0.1312 | -0.1197 | 0.9048 | -0.2700 | 0.2386 |
|  | Site: ED2 | 0.2427 | 0.2890 | 0.8397 | 0.4017 | -0.3173 | 0.8027 |
|  | Site: ED1 | 0.1870 | 0.2891 | 0.6469 | 0.5182 | -0.3731 | 0.7472 |
|  | Site: DUN | -0.1056 | 0.3204 | -0.3295 | 0.7420 | -0.7263 | 0.5152 |
|  | Site: GLA | -0.1008 | 0.2871 | -0.3510 | 0.7258 | -0.6570 | 0.4555 |
|  | DMT | 0.2072 | 0.1386 | 1.4943 | 0.1361 | -0.0615 | 0.4758 |
| NAWM isthmus cingulate L | Intercept | 0.5524 | 0.2662 | 2.0746 | **0.0389** | 0.0365 | 1.0682 |
|  | Time | -0.0775 | 0.0136 | -5.6997 | **<0.0001** | -0.1042 | -0.0508 |
|  | WML change | -0.0474 | 0.0578 | -0.8194 | 0.4132 | -0.1595 | 0.0647 |
|  | Age | 0.0427 | 0.0580 | 0.7361 | 0.4623 | -0.0696 | 0.1550 |
|  | Sex: Male | -0.1698 | 0.1313 | -1.2926 | 0.1971 | -0.4243 | 0.0847 |
|  | Site: ED2 | -0.4720 | 0.2876 | -1.6411 | 0.1018 | -1.0293 | 0.0853 |
|  | Site: ED1 | -0.6355 | 0.2879 | -2.2075 | **0.0280** | -1.1933 | -0.0777 |
|  | Site: DUN | -0.5616 | 0.3206 | -1.7516 | 0.0808 | -1.1829 | 0.0596 |
|  | Site: GLA | -0.5994 | 0.2858 | -2.0969 | **0.0368** | -1.1532 | -0.0455 |
|  | DMT | 0.1013 | 0.1379 | 0.7347 | 0.4631 | -0.1658 | 0.3684 |
| NAWM isthmus cingulate R | Intercept | 0.4563 | 0.2643 | 1.7266 | 0.0853 | -0.0557 | 0.9683 |
|  | Time | -0.0824 | 0.0123 | -6.6934 | **<0.0001** | -0.1065 | -0.0582 |
|  | WML change | -0.0546 | 0.0576 | -0.9490 | 0.3434 | -0.1662 | 0.0569 |
|  | Age | 0.0776 | 0.0578 | 1.3441 | 0.1799 | -0.0343 | 0.1895 |
|  | Sex: Male | -0.2808 | 0.1310 | -2.1429 | **0.0329** | -0.5346 | -0.0269 |
|  | Site: ED2 | -0.0983 | 0.2853 | -0.3444 | 0.7308 | -0.6510 | 0.4544 |
|  | Site: ED1 | -0.3391 | 0.2861 | -1.1853 | 0.2368 | -0.8934 | 0.2152 |
|  | Site: DUN | -0.4621 | 0.3180 | -1.4532 | 0.1472 | -1.0783 | 0.1540 |
|  | Site: GLA | -0.3879 | 0.2835 | -1.3683 | 0.1722 | -0.9372 | 0.1614 |
|  | DMT | -0.0637 | 0.1371 | -0.4646 | 0.6425 | -0.3293 | 0.2019 |
| NAWM postcentral L | Intercept | 0.6543 | 0.2564 | 2.5515 | **0.0112** | 0.1574 | 1.1511 |
|  | Time | -0.0666 | 0.0189 | -3.5125 | **0.0005** | -0.1037 | -0.0294 |
|  | WML change | 0.0033 | 0.0558 | 0.0588 | 0.9532 | -0.1048 | 0.1114 |
|  | Age | 0.0221 | 0.0561 | 0.3940 | 0.6939 | -0.0867 | 0.1309 |
|  | Sex: Male | -0.1467 | 0.1262 | -1.1621 | 0.2461 | -0.3912 | 0.0979 |
|  | Site: ED2 | -0.2338 | 0.2775 | -0.8427 | 0.4000 | -0.7715 | 0.3038 |
|  | Site: ED1 | -0.9324 | 0.2773 | -3.3625 | **0.0009** | -1.4697 | -0.3952 |
|  | Site: DUN | -0.7953 | 0.3076 | -2.5856 | **0.0102** | -1.3912 | -0.1993 |
|  | Site: GLA | -0.6112 | 0.2758 | -2.2162 | **0.0274** | -1.1456 | -0.0769 |
|  | DMT | 0.0108 | 0.1334 | 0.0807 | 0.9357 | -0.2477 | 0.2693 |
| NAWM postcentral R | Intercept | 0.4180 | 0.2567 | 1.6287 | 0.1044 | -0.0793 | 0.9153 |
|  | Time | -0.0523 | 0.0194 | -2.6940 | **0.0074** | -0.0904 | -0.0142 |
|  | WML change | 0.0330 | 0.0556 | 0.5930 | 0.5536 | -0.0748 | 0.1407 |
|  | Age | 0.0360 | 0.0559 | 0.6451 | 0.5194 | -0.0722 | 0.1443 |
|  | Sex: Male | 0.0040 | 0.1259 | 0.0321 | 0.9744 | -0.2400 | 0.2481 |
|  | Site: ED2 | 0.0151 | 0.2772 | 0.0545 | 0.9565 | -0.5220 | 0.5523 |
|  | Site: ED1 | -0.7153 | 0.2780 | -2.5731 | **0.0106** | -1.2540 | -0.1767 |
|  | Site: DUN | -0.5471 | 0.3076 | -1.7788 | 0.0763 | -1.1431 | 0.0488 |
|  | Site: GLA | -0.4751 | 0.2756 | -1.7239 | 0.0857 | -1.0091 | 0.0589 |
|  | DMT | 0.0167 | 0.1334 | 0.1253 | 0.9004 | -0.2418 | 0.2752 |
| NAWM posterior cingulate L | Intercept | 0.1231 | 0.2625 | 0.4690 | 0.6394 | -0.3856 | 0.6318 |
|  | Time | -0.0928 | 0.0131 | -7.0628 | **<0.0001** | -0.1186 | -0.0670 |
|  | WML change | 0.0040 | 0.0570 | 0.0702 | 0.9441 | -0.1065 | 0.1145 |
|  | Age | -0.0559 | 0.0572 | -0.9788 | 0.3285 | -0.1667 | 0.0548 |
|  | Sex: Male | -0.2357 | 0.1295 | -1.8197 | 0.0698 | -0.4866 | 0.0153 |
|  | Site: ED2 | 0.3942 | 0.2836 | 1.3900 | 0.1655 | -0.1553 | 0.9437 |
|  | Site: ED1 | -0.0242 | 0.2839 | -0.0854 | 0.9320 | -0.5742 | 0.5258 |
|  | Site: DUN | -0.1337 | 0.3162 | -0.4228 | 0.6728 | -0.7462 | 0.4789 |
|  | Site: GLA | 0.1425 | 0.2818 | 0.5055 | 0.6136 | -0.4036 | 0.6886 |
|  | DMT | -0.1946 | 0.1359 | -1.4316 | 0.1533 | -0.4580 | 0.0688 |
| NAWM posterior cingulate R | Intercept | 0.4998 | 0.2521 | 1.9827 | **0.0483** | 0.0114 | 0.9882 |
|  | Time | -0.1095 | 0.0134 | -8.1935 | **<0.0001** | -0.1358 | -0.0833 |
|  | WML change | 0.0564 | 0.0548 | 1.0290 | 0.3043 | -0.0498 | 0.1627 |
|  | Age | -0.1762 | 0.0551 | -3.1976 | **0.0015** | -0.2830 | -0.0694 |
|  | Sex: Male | -0.2947 | 0.1244 | -2.3700 | **0.0184** | -0.5357 | -0.0538 |
|  | Site: ED2 | 0.0618 | 0.2725 | 0.2269 | 0.8206 | -0.4660 | 0.5897 |
|  | Site: ED1 | -0.6911 | 0.2730 | -2.5321 | **0.0118** | -1.2199 | -0.1623 |
|  | Site: DUN | -0.0079 | 0.3033 | -0.0262 | 0.9791 | -0.5955 | 0.5797 |
|  | Site: GLA | -0.3645 | 0.2706 | -1.3466 | 0.1791 | -0.8888 | 0.1599 |
|  | DMT | -0.1136 | 0.1310 | -0.8678 | 0.3862 | -0.3673 | 0.1401 |
| NAWM precuneus L | Intercept | 0.7088 | 0.2646 | 2.6785 | **0.0078** | 0.1961 | 1.2215 |
|  | Time | -0.1048 | 0.0118 | -8.8600 | **<0.0001** | -0.1280 | -0.0816 |
|  | WML change | -0.1081 | 0.0576 | -1.8759 | 0.0616 | -0.2198 | 0.0035 |
|  | Age | -0.0164 | 0.0579 | -0.2839 | 0.7767 | -0.1287 | 0.0958 |
|  | Sex: Male | -0.0353 | 0.1303 | -0.2707 | 0.7868 | -0.2877 | 0.2172 |
|  | Site: ED2 | -0.5885 | 0.2866 | -2.0533 | **0.0409** | -1.1438 | -0.0332 |
|  | Site: ED1 | -0.5656 | 0.2863 | -1.9752 | **0.0491** | -1.1203 | -0.0108 |
|  | Site: DUN | -0.7998 | 0.3175 | -2.5186 | **0.0123** | -1.4151 | -0.1845 |
|  | Site: GLA | -0.7469 | 0.2848 | -2.6228 | **0.0092** | -1.2987 | -0.1952 |
|  | DMT | -0.0126 | 0.1375 | -0.0918 | 0.9269 | -0.2791 | 0.2538 |
| NAWM precuneus R | Intercept | 0.3132 | 0.2683 | 1.1673 | 0.2440 | -0.2067 | 0.8330 |
|  | Time | -0.1076 | 0.0106 | -10.1784 | **<0.0001** | -0.1283 | -0.0868 |
|  | WML change | -0.0523 | 0.0583 | -0.8969 | 0.3705 | -0.1651 | 0.0606 |
|  | Age | 0.0106 | 0.0585 | 0.1809 | 0.8565 | -0.1028 | 0.1239 |
|  | Sex: Male | 0.0568 | 0.1324 | 0.4291 | 0.6681 | -0.1997 | 0.3133 |
|  | Site: ED2 | -0.1202 | 0.2903 | -0.4141 | 0.6791 | -0.6826 | 0.4422 |
|  | Site: ED1 | -0.2854 | 0.2904 | -0.9829 | 0.3265 | -0.8481 | 0.2772 |
|  | Site: DUN | -0.4385 | 0.3213 | -1.3646 | 0.1734 | -1.0611 | 0.1841 |
|  | Site: GLA | -0.2382 | 0.2880 | -0.8271 | 0.4088 | -0.7961 | 0.3198 |
|  | DMT | -0.0545 | 0.1395 | -0.3905 | 0.6964 | -0.3248 | 0.2158 |
| NAWM superior parietal L | Intercept | 0.1541 | 0.2663 | 0.5789 | 0.5631 | -0.3618 | 0.6700 |
|  | Time | -0.0433 | 0.0106 | -4.0786 | **0.0001** | -0.0641 | -0.0225 |
|  | WML change | 0.0228 | 0.0579 | 0.3932 | 0.6944 | -0.0894 | 0.1349 |
|  | Age | -0.0040 | 0.0581 | -0.0693 | 0.9448 | -0.1166 | 0.1085 |
|  | Sex: Male | -0.2209 | 0.1320 | -1.6732 | 0.0953 | -0.4767 | 0.0349 |
|  | Site: ED2 | -0.0666 | 0.2876 | -0.2316 | 0.8170 | -0.6238 | 0.4906 |
|  | Site: ED1 | -0.2882 | 0.2882 | -0.9998 | 0.3182 | -0.8467 | 0.2703 |
|  | Site: DUN | -0.4968 | 0.3190 | -1.5574 | 0.1204 | -1.1149 | 0.1213 |
|  | Site: GLA | -0.1457 | 0.2859 | -0.5095 | 0.6108 | -0.6997 | 0.4083 |
|  | DMT | 0.1542 | 0.1380 | 1.1168 | 0.2650 | -0.1133 | 0.4216 |
| NAWM superior parietal R | Intercept | 0.2353 | 0.2641 | 0.8910 | 0.3736 | -0.2764 | 0.7470 |
|  | Time | -0.0317 | 0.0091 | -3.4804 | **0.0006** | -0.0496 | -0.0138 |
|  | WML change | -0.0101 | 0.0572 | -0.1762 | 0.8603 | -0.1209 | 0.1008 |
|  | Age | -0.0114 | 0.0574 | -0.1978 | 0.8433 | -0.1226 | 0.0999 |
|  | Sex: Male | -0.2957 | 0.1296 | -2.2812 | **0.0232** | -0.5469 | -0.0445 |
|  | Site: ED2 | 0.0711 | 0.2854 | 0.2490 | 0.8035 | -0.4820 | 0.6241 |
|  | Site: ED1 | -0.2123 | 0.2857 | -0.7430 | 0.4581 | -0.7658 | 0.3413 |
|  | Site: DUN | -0.4926 | 0.3166 | -1.5561 | 0.1207 | -1.1060 | 0.1208 |
|  | Site: GLA | 0.0004 | 0.2837 | 0.0013 | 0.9989 | -0.5493 | 0.5500 |
|  | DMT | -0.0805 | 0.1368 | -0.5889 | 0.5564 | -0.3456 | 0.1845 |
| NAWM supramarginal L | Intercept | 0.4007 | 0.2649 | 1.5128 | 0.1314 | -0.1125 | 0.9140 |
|  | Time | -0.0226 | 0.0091 | -2.4920 | **0.0132** | -0.0404 | -0.0048 |
|  | WML change | -0.0082 | 0.0574 | -0.1429 | 0.8864 | -0.1195 | 0.1031 |
|  | Age | -0.0067 | 0.0577 | -0.1157 | 0.9080 | -0.1185 | 0.1051 |
|  | Sex: Male | 0.2061 | 0.1307 | 1.5767 | 0.1159 | -0.0472 | 0.4593 |
|  | Site: ED2 | -0.3129 | 0.2861 | -1.0934 | 0.2751 | -0.8673 | 0.2415 |
|  | Site: ED1 | -0.3713 | 0.2868 | -1.2947 | 0.1964 | -0.9270 | 0.1844 |
|  | Site: DUN | -0.8664 | 0.3174 | -2.7299 | **0.0067** | -1.4813 | -0.2514 |
|  | Site: GLA | -0.5209 | 0.2844 | -1.8318 | 0.0680 | -1.0719 | 0.0301 |
|  | DMT | 0.0158 | 0.1373 | 0.1151 | 0.9084 | -0.2503 | 0.2819 |
| NAWM supramarginal R | Intercept | 0.1603 | 0.2657 | 0.6036 | 0.5466 | -0.3544 | 0.6751 |
|  | Time | -0.0258 | 0.0080 | -3.2215 | **0.0014** | -0.0415 | -0.0101 |
|  | WML change | 0.0230 | 0.0576 | 0.3991 | 0.6901 | -0.0886 | 0.1346 |
|  | Age | 0.0215 | 0.0579 | 0.3709 | 0.7110 | -0.0906 | 0.1335 |
|  | Sex: Male | -0.0018 | 0.1310 | -0.0140 | 0.9888 | -0.2557 | 0.2520 |
|  | Site: ED2 | -0.0087 | 0.2871 | -0.0302 | 0.9759 | -0.5649 | 0.5476 |
|  | Site: ED1 | -0.4434 | 0.2874 | -1.5431 | 0.1238 | -1.0003 | 0.1134 |
|  | Site: DUN | -0.2046 | 0.3184 | -0.6426 | 0.5209 | -0.8217 | 0.4124 |
|  | Site: GLA | -0.4384 | 0.2854 | -1.5359 | 0.1256 | -0.9915 | 0.1147 |
|  | DMT | 0.2028 | 0.1376 | 1.4736 | 0.1416 | -0.0638 | 0.4694 |
| GM cuneus L | Intercept | -0.3733 | 0.2641 | -1.4133 | 0.1586 | -0.8851 | 0.1385 |
|  | Time | -0.0329 | 0.0094 | -3.5176 | **0.0005** | -0.0513 | -0.0145 |
|  | WML change | -0.0078 | 0.0572 | -0.1367 | 0.8914 | -0.1187 | 0.1031 |
|  | Age | -0.1230 | 0.0574 | -2.1417 | **0.0330** | -0.2343 | -0.0117 |
|  | Sex: Male | 0.1171 | 0.1297 | 0.9027 | 0.3674 | -0.1342 | 0.3683 |
|  | Site: ED2 | 0.4708 | 0.2855 | 1.6491 | 0.1001 | -0.0824 | 1.0240 |
|  | Site: ED1 | 0.3144 | 0.2858 | 1.1002 | 0.2721 | -0.2393 | 0.8681 |
|  | Site: DUN | 0.0442 | 0.3167 | 0.1395 | 0.8891 | -0.5694 | 0.6578 |
|  | Site: GLA | 0.4840 | 0.2837 | 1.7059 | 0.0890 | -0.0658 | 1.0338 |
|  | DMT | -0.0134 | 0.1368 | -0.0981 | 0.9219 | -0.2785 | 0.2517 |
| GM cuneus R | Intercept | -0.1336 | 0.2656 | -0.5029 | 0.6154 | -0.6483 | 0.3811 |
|  | Time | -0.0390 | 0.0112 | -3.4708 | **0.0006** | -0.0610 | -0.0169 |
|  | WML change | -0.0277 | 0.0575 | -0.4819 | 0.6302 | -0.1392 | 0.0838 |
|  | Age | -0.1352 | 0.0578 | -2.3408 | **0.0199** | -0.2471 | -0.0233 |
|  | Sex: Male | -0.0314 | 0.1304 | -0.2407 | 0.8100 | -0.2841 | 0.2213 |
|  | Site: ED2 | 0.0816 | 0.2871 | 0.2844 | 0.7763 | -0.4746 | 0.6379 |
|  | Site: ED1 | 0.0813 | 0.2874 | 0.2828 | 0.7775 | -0.4755 | 0.6381 |
|  | Site: DUN | -0.0773 | 0.3184 | -0.2429 | 0.8083 | -0.6943 | 0.5397 |
|  | Site: GLA | 0.1811 | 0.2853 | 0.6348 | 0.5260 | -0.3717 | 0.7340 |
|  | DMT | 0.0844 | 0.1376 | 0.6131 | 0.5403 | -0.1822 | 0.3509 |
| GM lateral occipital L | Intercept | -0.4915 | 0.2564 | -1.9170 | 0.0562 | -0.9882 | 0.0053 |
|  | Time | -0.0631 | 0.0140 | -4.5204 | **<0.0001** | -0.0906 | -0.0357 |
|  | WML change | 0.0334 | 0.0557 | 0.5987 | 0.5498 | -0.0746 | 0.1413 |
|  | Age | -0.2500 | 0.0559 | -4.4723 | **<0.0001** | -0.3584 | -0.1417 |
|  | Sex: Male | 0.0114 | 0.1260 | 0.0903 | 0.9281 | -0.2327 | 0.2555 |
|  | Site: ED2 | 0.7455 | 0.2772 | 2.6892 | **0.0076** | 0.2084 | 1.2826 |
|  | Site: ED1 | 0.4279 | 0.2773 | 1.5430 | 0.1239 | -0.1094 | 0.9653 |
|  | Site: DUN | 0.6460 | 0.3073 | 2.1021 | **0.0364** | 0.0506 | 1.2415 |
|  | Site: GLA | 0.7057 | 0.2755 | 2.5619 | **0.0109** | 0.1720 | 1.2394 |
|  | DMT | -0.1203 | 0.1329 | -0.9053 | 0.3660 | -0.3779 | 0.1372 |
| GM lateral occipital R | Intercept | -0.7095 | 0.2491 | -2.8486 | **0.0047** | -1.1922 | -0.2269 |
|  | Time | -0.0269 | 0.0120 | -2.2446 | **0.0255** | -0.0504 | -0.0034 |
|  | WML change | -0.0250 | 0.0541 | -0.4619 | 0.6445 | -0.1297 | 0.0798 |
|  | Age | -0.2502 | 0.0542 | -4.6141 | **<0.0001** | -0.3553 | -0.1451 |
|  | Sex: Male | 0.1456 | 0.1229 | 1.1843 | 0.2372 | -0.0926 | 0.3838 |
|  | Site: ED2 | 1.1356 | 0.2694 | 4.2154 | **<0.0001** | 0.6136 | 1.6576 |
|  | Site: ED1 | 0.5920 | 0.2694 | 2.1976 | **0.0287** | 0.0700 | 1.1139 |
|  | Site: DUN | 0.9845 | 0.2985 | 3.2980 | **0.0011** | 0.4061 | 1.5628 |
|  | Site: GLA | 1.0513 | 0.2675 | 3.9307 | **0.0001** | 0.5331 | 1.5696 |
|  | DMT | -0.2951 | 0.1291 | -2.2866 | **0.0229** | -0.5452 | -0.0450 |
| GM lingual L | Intercept | -0.1215 | 0.2625 | -0.4628 | 0.6438 | -0.6301 | 0.3871 |
|  | Time | -0.0405 | 0.0105 | -3.8496 | **0.0001** | -0.0611 | -0.0198 |
|  | WML change | -0.1104 | 0.0571 | -1.9340 | 0.0540 | -0.2210 | 0.0002 |
|  | Age | -0.1624 | 0.0573 | -2.8325 | **0.0049** | -0.2735 | -0.0513 |
|  | Sex: Male | 0.0815 | 0.1296 | 0.6289 | 0.5299 | -0.1696 | 0.3326 |
|  | Site: ED2 | 0.1820 | 0.2837 | 0.6416 | 0.5216 | -0.3677 | 0.7317 |
|  | Site: ED1 | 0.2409 | 0.2844 | 0.8470 | 0.3977 | -0.3101 | 0.7918 |
|  | Site: DUN | -0.1580 | 0.3145 | -0.5023 | 0.6158 | -0.7674 | 0.4514 |
|  | Site: GLA | 0.1392 | 0.2818 | 0.4939 | 0.6217 | -0.4069 | 0.6853 |
|  | DMT | -0.0158 | 0.1364 | -0.1155 | 0.9081 | -0.2801 | 0.2486 |
| GM lingual R | Intercept | -0.0859 | 0.2626 | -0.3270 | 0.7439 | -0.5947 | 0.4230 |
|  | Time | -0.0361 | 0.0101 | -3.5790 | **0.0004** | -0.0559 | -0.0163 |
|  | WML change | -0.0845 | 0.0569 | -1.4855 | 0.1384 | -0.1947 | 0.0257 |
|  | Age | -0.1441 | 0.0571 | -2.5228 | **0.0121** | -0.2547 | -0.0334 |
|  | Sex: Male | -0.1934 | 0.1289 | -1.5002 | 0.1346 | -0.4432 | 0.0564 |
|  | Site: ED2 | 0.2670 | 0.2838 | 0.9407 | 0.3476 | -0.2830 | 0.8170 |
|  | Site: ED1 | 0.1923 | 0.2841 | 0.6769 | 0.4990 | -0.3582 | 0.7428 |
|  | Site: DUN | -0.1320 | 0.3148 | -0.4194 | 0.6753 | -0.7420 | 0.4780 |
|  | Site: GLA | 0.0089 | 0.2821 | 0.0315 | 0.9749 | -0.5377 | 0.5555 |
|  | DMT | 0.0778 | 0.1360 | 0.5717 | 0.5679 | -0.1858 | 0.3413 |
| GM pericalcarine L | Intercept | -0.3509 | 0.2585 | -1.3578 | 0.1755 | -0.8517 | 0.1498 |
|  | Time | -0.0310 | 0.0138 | -2.2411 | **0.0257** | -0.0581 | -0.0038 |
|  | WML change | -0.0864 | 0.0562 | -1.5356 | 0.1257 | -0.1953 | 0.0226 |
|  | Age | -0.1133 | 0.0564 | -2.0081 | **0.0455** | -0.2226 | -0.0040 |
|  | Sex: Male | -0.2257 | 0.1275 | -1.7698 | 0.0778 | -0.4728 | 0.0214 |
|  | Site: ED2 | 0.5250 | 0.2795 | 1.8784 | 0.0613 | -0.0165 | 1.0665 |
|  | Site: ED1 | 0.6407 | 0.2797 | 2.2904 | **0.0227** | 0.0987 | 1.1826 |
|  | Site: DUN | -0.0267 | 0.3098 | -0.0861 | 0.9314 | -0.6269 | 0.5735 |
|  | Site: GLA | 0.3657 | 0.2777 | 1.3167 | 0.1889 | -0.1724 | 0.9038 |
|  | DMT | 0.0227 | 0.1341 | 0.1696 | 0.8654 | -0.2370 | 0.2825 |
| GM pericalcarine R | Intercept | -0.1726 | 0.2586 | -0.6675 | 0.5049 | -0.6736 | 0.3284 |
|  | Time | -0.0441 | 0.0146 | -3.0180 | **0.0028** | -0.0729 | -0.0154 |
|  | WML change | -0.0659 | 0.0562 | -1.1732 | 0.2416 | -0.1748 | 0.0430 |
|  | Age | -0.1021 | 0.0564 | -1.8085 | 0.0715 | -0.2114 | 0.0073 |
|  | Sex: Male | -0.3184 | 0.1275 | -2.4966 | **0.0131** | -0.5655 | -0.0713 |
|  | Site: ED2 | 0.1343 | 0.2794 | 0.4807 | 0.6311 | -0.4070 | 0.6755 |
|  | Site: ED1 | 0.4506 | 0.2798 | 1.6103 | 0.1084 | -0.0916 | 0.9928 |
|  | Site: DUN | -0.0543 | 0.3099 | -0.1753 | 0.8610 | -0.6547 | 0.5461 |
|  | Site: GLA | 0.0097 | 0.2778 | 0.0348 | 0.9723 | -0.5285 | 0.5479 |
|  | DMT | 0.1901 | 0.1341 | 1.4170 | 0.1575 | -0.0698 | 0.4499 |
| NAWM cuneus L | Intercept | 0.1761 | 0.2641 | 0.6668 | 0.5054 | -0.3355 | 0.6877 |
|  | Time | -0.0331 | 0.0137 | -2.4145 | **0.0163** | -0.0599 | -0.0062 |
|  | WML change | -0.0510 | 0.0576 | -0.8868 | 0.3759 | -0.1625 | 0.0605 |
|  | Age | 0.0271 | 0.0579 | 0.4682 | 0.6400 | -0.0851 | 0.1394 |
|  | Sex: Male | 0.2129 | 0.1300 | 1.6374 | 0.1026 | -0.0390 | 0.4648 |
|  | Site: ED2 | 0.0156 | 0.2854 | 0.0547 | 0.9564 | -0.5373 | 0.5685 |
|  | Site: ED1 | -0.4074 | 0.2867 | -1.4210 | 0.1563 | -0.9630 | 0.1481 |
|  | Site: DUN | -0.1219 | 0.3169 | -0.3847 | 0.7007 | -0.7359 | 0.4921 |
|  | Site: GLA | -0.0798 | 0.2843 | -0.2807 | 0.7791 | -0.6306 | 0.4710 |
|  | DMT | -0.1050 | 0.1380 | -0.7610 | 0.4472 | -0.3725 | 0.1624 |
| NAWM cuneus R | Intercept | 0.4553 | 0.2638 | 1.7256 | 0.0854 | -0.0559 | 0.9664 |
|  | Time | -0.0030 | 0.0145 | -0.2043 | 0.8383 | -0.0315 | 0.0256 |
|  | WML change | -0.0153 | 0.0573 | -0.2667 | 0.7899 | -0.1264 | 0.0958 |
|  | Age | 0.0563 | 0.0576 | 0.9785 | 0.3286 | -0.0552 | 0.1679 |
|  | Sex: Male | 0.0994 | 0.1301 | 0.7637 | 0.4456 | -0.1527 | 0.3515 |
|  | Site: ED2 | -0.2645 | 0.2849 | -0.9282 | 0.3541 | -0.8165 | 0.2876 |
|  | Site: ED1 | -0.6719 | 0.2863 | -2.3467 | **0.0196** | -1.2266 | -0.1172 |
|  | Site: DUN | -0.5218 | 0.3176 | -1.6430 | 0.1014 | -1.1371 | 0.0935 |
|  | Site: GLA | -0.3585 | 0.2834 | -1.2653 | 0.2067 | -0.9075 | 0.1905 |
|  | DMT | -0.0930 | 0.1377 | -0.6753 | 0.5000 | -0.3597 | 0.1738 |
| NAWM lateral occipital L | Intercept | -0.0402 | 0.2629 | -0.1530 | 0.8785 | -0.5497 | 0.4692 |
|  | Time | -0.0689 | 0.0143 | -4.7993 | **<0.0001** | -0.0970 | -0.0407 |
|  | WML change | 0.1219 | 0.0571 | 2.1358 | **0.0335** | 0.0113 | 0.2324 |
|  | Age | -0.1337 | 0.0573 | -2.3310 | **0.0204** | -0.2448 | -0.0226 |
|  | Sex: Male | 0.1857 | 0.1303 | 1.4246 | 0.1553 | -0.0668 | 0.4382 |
|  | Site: ED2 | 0.2066 | 0.2841 | 0.7271 | 0.4677 | -0.3439 | 0.7571 |
|  | Site: ED1 | -0.0684 | 0.2844 | -0.2406 | 0.8100 | -0.6195 | 0.4826 |
|  | Site: DUN | 0.3793 | 0.3148 | 1.2050 | 0.2291 | -0.2306 | 0.9892 |
|  | Site: GLA | 0.2103 | 0.2820 | 0.7458 | 0.4564 | -0.3361 | 0.7568 |
|  | DMT | -0.1676 | 0.1365 | -1.2284 | 0.2203 | -0.4320 | 0.0968 |
| NAWM lateral occipital R | Intercept | -0.3706 | 0.2608 | -1.4212 | 0.1563 | -0.8758 | 0.1346 |
|  | Time | -0.0468 | 0.0115 | -4.0644 | **0.0001** | -0.0694 | -0.0242 |
|  | WML change | 0.0040 | 0.0566 | 0.0707 | 0.9437 | -0.1058 | 0.1138 |
|  | Age | -0.1074 | 0.0568 | -1.8897 | 0.0598 | -0.2176 | 0.0027 |
|  | Sex: Male | 0.1749 | 0.1286 | 1.3598 | 0.1749 | -0.0743 | 0.4242 |
|  | Site: ED2 | 0.7410 | 0.2819 | 2.6286 | **0.0090** | 0.1948 | 1.2872 |
|  | Site: ED1 | 0.2583 | 0.2820 | 0.9159 | 0.3604 | -0.2881 | 0.8047 |
|  | Site: DUN | 0.7025 | 0.3139 | 2.2382 | **0.0259** | 0.0944 | 1.3107 |
|  | Site: GLA | 0.6647 | 0.2800 | 2.3738 | **0.0182** | 0.1222 | 1.2072 |
|  | DMT | -0.2815 | 0.1352 | -2.0820 | **0.0382** | -0.5434 | -0.0195 |
| NAWM lingual L | Intercept | 0.0639 | 0.2660 | 0.2401 | 0.8104 | -0.4516 | 0.5793 |
|  | Time | -0.1169 | 0.0151 | -7.7652 | **<0.0001** | -0.1464 | -0.0873 |
|  | WML change | -0.0050 | 0.0578 | -0.0856 | 0.9318 | -0.1170 | 0.1071 |
|  | Age | -0.0674 | 0.0580 | -1.1606 | 0.2467 | -0.1798 | 0.0451 |
|  | Sex: Male | -0.2105 | 0.1307 | -1.6104 | 0.1084 | -0.4637 | 0.0428 |
|  | Site: ED2 | 0.1312 | 0.2877 | 0.4561 | 0.6486 | -0.4261 | 0.6886 |
|  | Site: ED1 | -0.0925 | 0.2877 | -0.3214 | 0.7481 | -0.6500 | 0.4650 |
|  | Site: DUN | -0.0400 | 0.3189 | -0.1256 | 0.9001 | -0.6579 | 0.5778 |
|  | Site: GLA | 0.0463 | 0.2858 | 0.1621 | 0.8714 | -0.5074 | 0.6000 |
|  | DMT | 0.0356 | 0.1380 | 0.2579 | 0.7967 | -0.2318 | 0.3030 |
| NAWM lingual R | Intercept | -0.1106 | 0.2641 | -0.4187 | 0.6757 | -0.6223 | 0.4011 |
|  | Time | -0.0787 | 0.0132 | -5.9618 | **<0.0001** | -0.1047 | -0.0528 |
|  | WML change | -0.0267 | 0.0573 | -0.4659 | 0.6416 | -0.1377 | 0.0843 |
|  | Age | -0.0019 | 0.0575 | -0.0334 | 0.9734 | -0.1133 | 0.1095 |
|  | Sex: Male | -0.2464 | 0.1297 | -1.9000 | 0.0584 | -0.4977 | 0.0049 |
|  | Site: ED2 | 0.3624 | 0.2856 | 1.2690 | 0.2054 | -0.1909 | 0.9158 |
|  | Site: ED1 | -0.0173 | 0.2857 | -0.0606 | 0.9517 | -0.5709 | 0.5363 |
|  | Site: DUN | -0.0850 | 0.3166 | -0.2684 | 0.7886 | -0.6984 | 0.5285 |
|  | Site: GLA | 0.0342 | 0.2837 | 0.1205 | 0.9041 | -0.5155 | 0.5839 |
|  | DMT | 0.1773 | 0.1369 | 1.2953 | 0.1962 | -0.0879 | 0.4426 |
| NAWM pericalcarine L | Intercept | 0.3322 | 0.2649 | 1.2539 | 0.2108 | -0.1811 | 0.8455 |
|  | Time | -0.1607 | 0.0127 | -12.6755 | **<0.0001** | -0.1855 | -0.1358 |
|  | WML change | -0.0379 | 0.0575 | -0.6594 | 0.5101 | -0.1493 | 0.0735 |
|  | Age | -0.0953 | 0.0577 | -1.6514 | 0.0997 | -0.2071 | 0.0165 |
|  | Sex: Male | -0.1599 | 0.1301 | -1.2288 | 0.2201 | -0.4119 | 0.0922 |
|  | Site: ED2 | -0.1772 | 0.2865 | -0.6186 | 0.5366 | -0.7324 | 0.3779 |
|  | Site: ED1 | -0.3486 | 0.2866 | -1.2165 | 0.2247 | -0.9039 | 0.2066 |
|  | Site: DUN | -0.4753 | 0.3176 | -1.4967 | 0.1355 | -1.0907 | 0.1400 |
|  | Site: GLA | -0.1743 | 0.2846 | -0.6125 | 0.5407 | -0.7256 | 0.3771 |
|  | DMT | 0.0383 | 0.1374 | 0.2786 | 0.7808 | -0.2280 | 0.3045 |
| NAWM pericalcarine R | Intercept | -0.1195 | 0.2632 | -0.4539 | 0.6502 | -0.6295 | 0.3906 |
|  | Time | -0.1376 | 0.0120 | -11.5135 | **<0.0001** | -0.1610 | -0.1141 |
|  | WML change | -0.0604 | 0.0572 | -1.0560 | 0.2918 | -0.1712 | 0.0504 |
|  | Age | -0.0400 | 0.0574 | -0.6972 | 0.4862 | -0.1513 | 0.0712 |
|  | Sex: Male | -0.1092 | 0.1293 | -0.8445 | 0.3991 | -0.3597 | 0.1413 |
|  | Site: ED2 | 0.3249 | 0.2847 | 1.1414 | 0.2546 | -0.2266 | 0.8765 |
|  | Site: ED1 | -0.1317 | 0.2853 | -0.4616 | 0.6447 | -0.6844 | 0.4210 |
|  | Site: DUN | -0.1318 | 0.3157 | -0.4176 | 0.6765 | -0.7434 | 0.4798 |
|  | Site: GLA | 0.0886 | 0.2828 | 0.3131 | 0.7544 | -0.4595 | 0.6366 |
|  | DMT | 0.2082 | 0.1371 | 1.5180 | 0.1300 | -0.0575 | 0.4738 |
| GM insula L | Intercept | -0.0391 | 0.2465 | -0.1586 | 0.8741 | -0.5166 | 0.4384 |
|  | Time | -0.0484 | 0.0169 | -2.8575 | **0.0046** | -0.0817 | -0.0152 |
|  | WML change | 0.0355 | 0.0536 | 0.6619 | 0.5086 | -0.0684 | 0.1393 |
|  | Age | -0.2396 | 0.0538 | -4.4539 | **<0.0001** | -0.3439 | -0.1354 |
|  | Sex: Male | -0.2374 | 0.1211 | -1.9609 | 0.0508 | -0.4720 | -0.0028 |
|  | Site: ED2 | 0.4918 | 0.2669 | 1.8428 | 0.0663 | -0.0253 | 1.0089 |
|  | Site: ED1 | -0.1720 | 0.2666 | -0.6452 | 0.5193 | -0.6886 | 0.3446 |
|  | Site: DUN | -0.3720 | 0.2953 | -1.2596 | 0.2088 | -0.9442 | 0.2002 |
|  | Site: GLA | -0.0432 | 0.2647 | -0.1631 | 0.8705 | -0.5560 | 0.4696 |
|  | DMT | 0.1512 | 0.1284 | 1.1772 | 0.2400 | -0.0976 | 0.3999 |
| GM insula R | Intercept | 0.4550 | 0.2437 | 1.8666 | 0.0629 | -0.0173 | 0.9272 |
|  | Time | -0.0420 | 0.0190 | -2.2143 | **0.0275** | -0.0792 | -0.0048 |
|  | WML change | 0.0271 | 0.0529 | 0.5135 | 0.6080 | -0.0753 | 0.1296 |
|  | Age | -0.3129 | 0.0530 | -5.8986 | **<0.0001** | -0.4156 | -0.2101 |
|  | Sex: Male | 0.1095 | 0.1197 | 0.9148 | 0.3610 | -0.1224 | 0.3413 |
|  | Site: ED2 | -0.0016 | 0.2633 | -0.0061 | 0.9951 | -0.5118 | 0.5086 |
|  | Site: ED1 | -0.6333 | 0.2636 | -2.4031 | **0.0169** | -1.1440 | -0.1227 |
|  | Site: DUN | -0.6400 | 0.2920 | -2.1915 | **0.0292** | -1.2059 | -0.0742 |
|  | Site: GLA | -0.5373 | 0.2617 | -2.0529 | **0.0409** | -1.0444 | -0.0302 |
|  | DMT | -0.0502 | 0.1262 | -0.3978 | 0.6910 | -0.2947 | 0.1943 |
| NAWM insula L | Intercept | 0.3637 | 0.2480 | 1.4666 | 0.1435 | -0.1167 | 0.8441 |
|  | Time | -0.0386 | 0.0144 | -2.6743 | **0.0079** | -0.0669 | -0.0103 |
|  | WML change | -0.1253 | 0.0539 | -2.3223 | **0.0209** | -0.2298 | -0.0208 |
|  | Age | 0.1087 | 0.0542 | 2.0051 | **0.0458** | 0.0037 | 0.2138 |
|  | Sex: Male | -0.3791 | 0.1221 | -3.1050 | **0.0021** | -0.6156 | -0.1425 |
|  | Site: ED2 | 0.3376 | 0.2681 | 1.2594 | 0.2089 | -0.1818 | 0.8570 |
|  | Site: ED1 | -0.4573 | 0.2681 | -1.7053 | 0.0892 | -0.9768 | 0.0622 |
|  | Site: DUN | -0.4295 | 0.2974 | -1.4442 | 0.1497 | -1.0057 | 0.1467 |
|  | Site: GLA | -0.4366 | 0.2669 | -1.6360 | 0.1029 | -0.9537 | 0.0805 |
|  | DMT | -0.0065 | 0.1287 | -0.0501 | 0.9600 | -0.2558 | 0.2429 |
| NAWM insula R | Intercept | 0.6696 | 0.2553 | 2.6231 | **0.0092** | 0.1750 | 1.1642 |
|  | Time | -0.0185 | 0.0183 | -1.0103 | 0.3131 | -0.0544 | 0.0174 |
|  | WML change | 0.0609 | 0.0554 | 1.1004 | 0.2720 | -0.0463 | 0.1682 |
|  | Age | -0.0492 | 0.0556 | -0.8844 | 0.3772 | -0.1569 | 0.0585 |
|  | Sex: Male | -0.1344 | 0.1253 | -1.0729 | 0.2842 | -0.3772 | 0.1083 |
|  | Site: ED2 | -0.0912 | 0.2759 | -0.3306 | 0.7412 | -0.6257 | 0.4433 |
|  | Site: ED1 | -0.7145 | 0.2759 | -2.5895 | **0.0101** | -1.2491 | -0.1799 |
|  | Site: DUN | -0.6313 | 0.3058 | -2.0645 | **0.0398** | -1.2238 | -0.0388 |
|  | Site: GLA | -0.5998 | 0.2742 | -2.1878 | **0.0294** | -1.1310 | -0.0686 |
|  | DMT | -0.1892 | 0.1324 | -1.4285 | 0.1542 | -0.4458 | 0.0674 |

B=standardised beta value, SE=standard error, CI=confidence interval for beta value, w0=baseline, w1=1-year follow-up, L=left, R=right, GM=grey matter, NAWM=normal-appearing white matter, WML=white matter lesion, DC=diencephalon, sts=superior temporal sulcus, ED1=Edinburgh site 1, ED2=Edinburgh site 2, GLA=Glasgow site, DUN=Dundee site, DMT=disease-modifying treatment

Significant (p<.05) Uuncorrected p-values <0.05 are shown in bold.
