# Supplementary material for: Patterns of brain atrophy in recently-diagnosed relapsing-remitting multiple sclerosis
Source: PLoS One. 2023 Jul 28;18(7):e0288967. doi: 10.1371/journal.pone.0288967 (PMC10381059; doi:10.1371/journal.pone.0288967)
Supplement: S4 Table — (DOCX) [file pone.0288967.s004.docx]

**S4 Table.** **Regional GM and NAWM volume results for change over time (w1-w0)**. This was assessed using linear mixed-effects models, corrected for age, sex, imaging site, DMT status at w1 and WML change. Standardised regression coeffients are shown. Mean and SD are shown for volumes as % of intracranial volume (%ICV), without covariate correction.

|  |  | B_standardised_ | SE | df | t | p-value | CI 2.5. | CI 97.5. | Mean w0  (%ICV) | SD w0  (%ICV) | Mean w1  (%ICV) | SD w1  (%ICV) | Mean 1-year % change |
| --- | --- | --- | --- | --- | --- | --- | --- | --- | --- | --- | --- | --- | --- |
| Frontal lobe | GM caudal anterior cingulate L | -0.0441 | 0.0078 | 312 | -5.6479 | **<0.0001** | -0.0595 | -0.0288 | 0.1153 | 0.0255 | 0.1143 | 0.0250 | -0.0441 |
|  | GM caudal anterior cingulate R | -0.0223 | 0.0073 | 314 | -3.0396 | **0.0026** | -0.0367 | -0.0079 | 0.1278 | 0.0269 | 0.1272 | 0.0267 | -0.0223 |
|  | GM caudal middle frontal L | -0.0325 | 0.0131 | 311 | -2.4729 | **0.0139** | -0.0582 | -0.0067 | 0.4142 | 0.0667 | 0.4120 | 0.0662 | -0.0325 |
|  | GM caudal middle frontal R | -0.0279 | 0.0132 | 312 | -2.1094 | **0.0357** | -0.0538 | -0.0019 | 0.3968 | 0.0626 | 0.3953 | 0.0621 | -0.0279 |
|  | GM frontal pole L | -0.0293 | 0.0203 | 311 | -1.4411 | 0.1506 | -0.0691 | 0.0106 | 0.0621 | 0.0089 | 0.0619 | 0.0091 | -0.0293 |
|  | GM frontal pole R | -0.0381 | 0.0193 | 312 | -1.9739 | 0.0493 | -0.0759 | -0.0002 | 0.0734 | 0.0100 | 0.0730 | 0.0096 | -0.0381 |
|  | GM lateral orbitofrontal L | -0.0684 | 0.0167 | 313 | -4.1102 | **0.0001** | -0.1011 | -0.0358 | 0.5036 | 0.0418 | 0.5008 | 0.0418 | -0.0684 |
|  | GM lateral orbitofrontal R | -0.0291 | 0.0197 | 313 | -1.4790 | 0.1402 | -0.0678 | 0.0095 | 0.4845 | 0.0404 | 0.4833 | 0.0414 | -0.0291 |
|  | GM medial orbitofrontal L | -0.0588 | 0.0220 | 311 | -2.6696 | **0.0080** | -0.1021 | -0.0156 | 0.3350 | 0.0333 | 0.3332 | 0.0337 | -0.0588 |
|  | GM medial orbitofrontal R | -0.0137 | 0.0214 | 312 | -0.6390 | 0.5233 | -0.0556 | 0.0283 | 0.3505 | 0.0336 | 0.3500 | 0.0339 | -0.0137 |
|  | GM paracentral L | -0.0157 | 0.0170 | 311 | -0.9226 | 0.3569 | -0.0490 | 0.0177 | 0.2346 | 0.0294 | 0.2342 | 0.0294 | -0.0157 |
|  | GM paracentral R | -0.0317 | 0.0159 | 311 | -1.9976 | 0.0466 | -0.0629 | -0.0006 | 0.2559 | 0.0305 | 0.2550 | 0.0310 | -0.0317 |
|  | GM pars opercularis L | -0.0388 | 0.0101 | 311 | -3.8407 | **0.0001** | -0.0586 | -0.0190 | 0.3052 | 0.0473 | 0.3035 | 0.0474 | -0.0388 |
|  | GM pars opercularis R | -0.0448 | 0.0115 | 312 | -3.9067 | **0.0001** | -0.0673 | -0.0223 | 0.2544 | 0.0349 | 0.2529 | 0.0350 | -0.0448 |
|  | GM pars orbitalis L | -0.0341 | 0.0151 | 313 | -2.2575 | **0.0247** | -0.0637 | -0.0044 | 0.1567 | 0.0196 | 0.1561 | 0.0195 | -0.0341 |
|  | GM pars orbitalis R | -0.0629 | 0.0146 | 312 | -4.3127 | **<0.0001** | -0.0915 | -0.0342 | 0.1825 | 0.0231 | 0.1812 | 0.0233 | -0.0629 |
|  | GM pars triangularis L | -0.0365 | 0.0116 | 313 | -3.1445 | **0.0018** | -0.0593 | -0.0137 | 0.2392 | 0.0349 | 0.2379 | 0.0340 | -0.0365 |
|  | GM pars triangularis R | -0.0422 | 0.0113 | 313 | -3.7344 | **0.0002** | -0.0644 | -0.0200 | 0.2782 | 0.0402 | 0.2766 | 0.0402 | -0.0422 |
|  | GM precentral L | -0.0108 | 0.0180 | 312 | -0.5980 | 0.5503 | -0.0462 | 0.0246 | 0.8799 | 0.0884 | 0.8790 | 0.0884 | -0.0108 |
|  | GM precentral R | -0.0040 | 0.0176 | 313 | -0.2269 | 0.8206 | -0.0385 | 0.0305 | 0.8651 | 0.0889 | 0.8648 | 0.0873 | -0.0040 |
|  | GM rostral anterior cingulate L | -0.0472 | 0.0124 | 311 | -3.7977 | **0.0002** | -0.0715 | -0.0228 | 0.1730 | 0.0260 | 0.1719 | 0.0259 | -0.0472 |
|  | GM rostral anterior cingulate R | -0.0195 | 0.0103 | 314 | -1.8930 | 0.0593 | -0.0398 | 0.0007 | 0.1270 | 0.0221 | 0.1266 | 0.0226 | -0.0195 |
|  | GM rostral middle frontal L | -0.0505 | 0.0143 | 310 | -3.5410 | **0.0005** | -0.0785 | -0.0225 | 1.0101 | 0.1139 | 1.0042 | 0.1120 | -0.0505 |
|  | GM rostral middle frontal R | -0.0317 | 0.0150 | 311 | -2.1159 | **0.0351** | -0.0611 | -0.0023 | 1.0209 | 0.1147 | 1.0172 | 0.1152 | -0.0317 |
|  | GM superior frontal L | -0.0624 | 0.0195 | 311 | -3.1906 | **0.0016** | -0.1007 | -0.0240 | 1.4641 | 0.1467 | 1.4554 | 0.1463 | -0.0624 |
|  | GM superior frontal R | -0.0348 | 0.0190 | 312 | -1.8294 | 0.0683 | -0.0721 | 0.0025 | 1.4018 | 0.1410 | 1.3962 | 0.1395 | -0.0348 |
|  | NAWM caudal anterior cingulate L | -0.0743 | 0.0134 | 312 | -5.5530 | **<0.0001** | -0.1006 | -0.0481 | 0.1689 | 0.0220 | 0.1673 | 0.0228 | -0.0743 |
|  | NAWM caudal anterior cingulate R | -0.0863 | 0.0134 | 312 | -6.4445 | **<0.0001** | -0.1126 | -0.0600 | 0.1710 | 0.0246 | 0.1690 | 0.0246 | -0.0863 |
|  | NAWM caudal middle frontal L | -0.0185 | 0.0089 | 313 | -2.0926 | **0.0372** | -0.0359 | -0.0011 | 0.4198 | 0.0535 | 0.4189 | 0.0544 | -0.0185 |
|  | NAWM caudal middle frontal R | -0.0254 | 0.0093 | 313 | -2.7177 | **0.0069** | -0.0437 | -0.0071 | 0.3832 | 0.0550 | 0.3818 | 0.0547 | -0.0254 |
|  | NAWM frontal pole L | -0.0111 | 0.0243 | 311 | -0.4555 | 0.6491 | -0.0589 | 0.0367 | 0.0186 | 0.0033 | 0.0186 | 0.0034 | -0.0111 |
|  | NAWM frontal pole R | 0.0042 | 0.0199 | 314 | 0.2122 | 0.8321 | -0.0348 | 0.0432 | 0.0229 | 0.0043 | 0.0229 | 0.0044 | 0.0042 |
|  | NAWM lateral orbitofrontal L | -0.0730 | 0.0109 | 311 | -6.6879 | **<0.0001** | -0.0945 | -0.0516 | 0.4369 | 0.0376 | 0.4343 | 0.0380 | -0.0730 |
|  | NAWM lateral orbitofrontal R | -0.0109 | 0.0221 | 312 | -0.4955 | 0.6206 | -0.0543 | 0.0324 | 0.4379 | 0.0387 | 0.4375 | 0.0400 | -0.0109 |
|  | NAWM medial orbitofrontal L | -0.0148 | 0.0223 | 313 | -0.6654 | 0.5063 | -0.0587 | 0.0290 | 0.2358 | 0.0333 | 0.2353 | 0.0336 | -0.0148 |
|  | NAWM medial orbitofrontal R | -0.0301 | 0.0214 | 313 | -1.4092 | 0.1598 | -0.0721 | 0.0118 | 0.2377 | 0.0247 | 0.2371 | 0.0249 | -0.0301 |
|  | NAWM paracentral L | -0.0505 | 0.0140 | 311 | -3.6198 | **0.0003** | -0.0779 | -0.0231 | 0.2485 | 0.0315 | 0.2471 | 0.0311 | -0.0505 |
|  | NAWM paracentral R | -0.0391 | 0.0136 | 310 | -2.8630 | **0.0045** | -0.0658 | -0.0123 | 0.2926 | 0.0398 | 0.2913 | 0.0397 | -0.0391 |
|  | NAWM pars opercularis L | -0.0279 | 0.0093 | 312 | -2.9909 | **0.0030** | -0.0461 | -0.0096 | 0.2253 | 0.0351 | 0.2244 | 0.0354 | -0.0279 |
|  | NAWM pars opercularis R | -0.0282 | 0.0085 | 313 | -3.3114 | **0.0010** | -0.0449 | -0.0115 | 0.2069 | 0.0291 | 0.2061 | 0.0293 | -0.0282 |
|  | NAWM pars orbitalis L | -0.0245 | 0.0175 | 312 | -1.4010 | 0.1622 | -0.0588 | 0.0098 | 0.0658 | 0.0097 | 0.0656 | 0.0095 | -0.0245 |
|  | NAWM pars orbitalis R | -0.0373 | 0.0176 | 311 | -2.1198 | **0.0348** | -0.0719 | -0.0028 | 0.0808 | 0.0117 | 0.0803 | 0.0118 | -0.0373 |
|  | NAWM pars triangularis L | -0.0155 | 0.0104 | 314 | -1.4843 | 0.1387 | -0.0359 | 0.0050 | 0.1949 | 0.0284 | 0.1945 | 0.0287 | -0.0155 |
|  | NAWM pars triangularis R | -0.0072 | 0.0109 | 313 | -0.6627 | 0.5080 | -0.0285 | 0.0141 | 0.2111 | 0.0299 | 0.2108 | 0.0300 | -0.0072 |
|  | NAWM precentral L | -0.0262 | 0.0150 | 311 | -1.7514 | 0.0809 | -0.0556 | 0.0032 | 0.8392 | 0.0890 | 0.8366 | 0.0888 | -0.0262 |
|  | NAWM precentral R | -0.0244 | 0.0134 | 311 | -1.8166 | 0.0702 | -0.0508 | 0.0020 | 0.8425 | 0.0872 | 0.8402 | 0.0897 | -0.0244 |
|  | NAWM rostral anterior cingulate L | -0.0329 | 0.0174 | 314 | -1.8938 | 0.0592 | -0.0670 | 0.0012 | 0.1675 | 0.0241 | 0.1667 | 0.0232 | -0.0329 |
|  | NAWM rostral anterior cingulate R | -0.0303 | 0.0145 | 313 | -2.0852 | **0.0379** | -0.0588 | -0.0018 | 0.1244 | 0.0169 | 0.1239 | 0.0171 | -0.0303 |
|  | NAWM rostral middle frontal L | -0.0311 | 0.0100 | 312 | -3.1023 | **0.0021** | -0.0507 | -0.0114 | 0.8169 | 0.0896 | 0.8141 | 0.0908 | -0.0311 |
|  | NAWM rostral middle frontal R | -0.0176 | 0.0103 | 311 | -1.7067 | 0.0889 | -0.0378 | 0.0026 | 0.8354 | 0.1016 | 0.8337 | 0.1027 | -0.0176 |
|  | NAWM superior frontal L^a^ | -0.0348 | 0.0137 | 313 | -2.5346 | **0.0117** | -0.0617 | -0.0079 | 1.1580 | 0.1209 | 1.1539 | 0.1230 | -0.0348 |
|  | NAWM superior frontal R^a^ | -0.0272 | 0.0131 | 313 | -2.0744 | **0.0389** | -0.0528 | -0.0015 | 1.1242 | 0.1175 | 1.1207 | 0.1193 | -0.0272 |
| Temporal lobe | GM banks sts L | -0.0399 | 0.0091 | 313 | -4.3896 | **<0.0001** | -0.0577 | -0.0220 | 0.1568 | 0.0254 | 0.1558 | 0.0253 | -0.0399 |
|  | GM banks sts R | -0.0338 | 0.0118 | 312 | -2.8521 | **0.0046** | -0.0570 | -0.0105 | 0.1430 | 0.0192 | 0.1424 | 0.0196 | -0.0338 |
|  | GM entorhinal L | -0.0066 | 0.0186 | 308 | -0.3544 | 0.7233 | -0.0430 | 0.0298 | 0.1324 | 0.0246 | 0.1322 | 0.0244 | -0.0066 |
|  | GM entorhinal R | -0.0252 | 0.0184 | 309 | -1.3689 | 0.1720 | -0.0612 | 0.0109 | 0.1278 | 0.0245 | 0.1271 | 0.0245 | -0.0252 |
|  | GM fusiform L | -0.0512 | 0.0128 | 311 | -4.0073 | **0.0001** | -0.0763 | -0.0261 | 0.6295 | 0.0699 | 0.6262 | 0.0691 | -0.0512 |
|  | GM fusiform R | -0.0307 | 0.0113 | 312 | -2.7082 | **0.0071** | -0.0529 | -0.0084 | 0.6161 | 0.0706 | 0.6141 | 0.0700 | -0.0307 |
|  | GM inferior temporal L | -0.0447 | 0.0127 | 311 | -3.5313 | **0.0005** | -0.0695 | -0.0199 | 0.7238 | 0.0864 | 0.7201 | 0.0849 | -0.0447 |
|  | GM inferior temporal R | -0.0278 | 0.0108 | 314 | -2.5747 | **0.0105** | -0.0490 | -0.0066 | 0.7031 | 0.0852 | 0.7007 | 0.0850 | -0.0278 |
|  | GM middle temporal L | -0.0535 | 0.0099 | 314 | -5.3785 | **<0.0001** | -0.0730 | -0.0340 | 0.7225 | 0.0886 | 0.7177 | 0.0881 | -0.0535 |
|  | GM middle temporal R | -0.0528 | 0.0114 | 313 | -4.6230 | **<0.0001** | -0.0752 | -0.0304 | 0.7927 | 0.0839 | 0.7883 | 0.0840 | -0.0528 |
|  | GM parahippocampal L | -0.0711 | 0.0127 | 311 | -5.5987 | **<0.0001** | -0.0961 | -0.0462 | 0.1401 | 0.0182 | 0.1388 | 0.0186 | -0.0711 |
|  | GM parahippocampal R | -0.0343 | 0.0130 | 314 | -2.6424 | **0.0086** | -0.0598 | -0.0088 | 0.1298 | 0.0161 | 0.1293 | 0.0163 | -0.0343 |
|  | GM superior temporal L | -0.0616 | 0.0123 | 313 | -5.0033 | **<0.0001** | -0.0858 | -0.0375 | 0.8128 | 0.0840 | 0.8078 | 0.0826 | -0.0616 |
|  | GM superior temporal R | -0.0524 | 0.0141 | 311 | -3.7128 | **0.0002** | -0.0801 | -0.0247 | 0.7688 | 0.0743 | 0.7649 | 0.0749 | -0.0524 |
|  | GM temporal pole L | -0.0721 | 0.0222 | 312 | -3.2482 | **0.0013** | -0.1156 | -0.0285 | 0.1580 | 0.0211 | 0.1564 | 0.0218 | -0.0721 |
|  | GM temporal pole R | -0.0210 | 0.0179 | 313 | -1.1683 | 0.2436 | -0.0562 | 0.0143 | 0.1589 | 0.0233 | 0.1586 | 0.0225 | -0.0210 |
|  | GM transverse temporal L | -0.0603 | 0.0120 | 313 | -5.0434 | **<0.0001** | -0.0837 | -0.0368 | 0.0781 | 0.0129 | 0.0773 | 0.0128 | -0.0603 |
|  | GM transverse temporal R | -0.0593 | 0.0146 | 313 | -4.0557 | **0.0001** | -0.0880 | -0.0306 | 0.0591 | 0.0094 | 0.0585 | 0.0092 | -0.0593 |
|  | NAWM banks sts L | -0.0282 | 0.0062 | 314 | -4.5194 | **<0.0001** | -0.0404 | -0.0159 | 0.1791 | 0.0384 | 0.1780 | 0.0385 | -0.0282 |
|  | NAWM banks sts R | -0.0356 | 0.0072 | 314 | -4.9273 | **<0.0001** | -0.0498 | -0.0214 | 0.1770 | 0.0301 | 0.1759 | 0.0305 | -0.0356 |
|  | NAWM entorhinal L | -0.0444 | 0.0138 | 313 | -3.2247 | **0.0014** | -0.0714 | -0.0174 | 0.0526 | 0.0141 | 0.0520 | 0.0138 | -0.0444 |
|  | NAWM entorhinal R | -0.0402 | 0.0166 | 313 | -2.4201 | **0.0161** | -0.0728 | -0.0076 | 0.0470 | 0.0109 | 0.0465 | 0.0107 | -0.0402 |
|  | NAWM fusiform L | -0.0925 | 0.0103 | 311 | -9.0060 | **<0.0001** | -0.1126 | -0.0723 | 0.4246 | 0.0520 | 0.4199 | 0.0537 | -0.0925 |
|  | NAWM fusiform R | -0.0832 | 0.0100 | 313 | -8.3457 | **<0.0001** | -0.1028 | -0.0636 | 0.4064 | 0.0507 | 0.4022 | 0.0516 | -0.0832 |
|  | NAWM inferior temporal L | -0.0578 | 0.0099 | 313 | -5.8325 | **<0.0001** | -0.0773 | -0.0384 | 0.4083 | 0.0492 | 0.4055 | 0.0500 | -0.0578 |
|  | NAWM inferior temporal R | -0.0490 | 0.0087 | 314 | -5.6095 | **<0.0001** | -0.0661 | -0.0318 | 0.3883 | 0.0488 | 0.3859 | 0.0493 | -0.0490 |
|  | NAWM middle temporal L | -0.0456 | 0.0103 | 310 | -4.4399 | **<0.0001** | -0.0657 | -0.0254 | 0.3447 | 0.0468 | 0.3427 | 0.0469 | -0.0456 |
|  | NAWM middle temporal R | -0.0522 | 0.0109 | 314 | -4.8055 | **<0.0001** | -0.0736 | -0.0309 | 0.3946 | 0.0438 | 0.3923 | 0.0442 | -0.0522 |
|  | NAWM parahippocampal L | -0.0587 | 0.0190 | 311 | -3.0862 | **0.0022** | -0.0961 | -0.0214 | 0.0908 | 0.0130 | 0.0901 | 0.0132 | -0.0587 |
|  | NAWM parahippocampal R | -0.0635 | 0.0134 | 312 | -4.7352 | **<0.0001** | -0.0899 | -0.0372 | 0.0939 | 0.0141 | 0.0931 | 0.0142 | -0.0635 |
|  | NAWM superior temporal L | -0.0489 | 0.0104 | 313 | -4.6780 | **<0.0001** | -0.0694 | -0.0284 | 0.4943 | 0.0570 | 0.4916 | 0.0577 | -0.0489 |
|  | NAWM superior temporal R | -0.0575 | 0.0128 | 312 | -4.4985 | **<0.0001** | -0.0825 | -0.0324 | 0.4126 | 0.0448 | 0.4102 | 0.0456 | -0.0575 |
|  | NAWM temporal pole L | -0.0990 | 0.0311 | 312 | -3.1791 | **0.0016** | -0.1601 | -0.0379 | 0.0377 | 0.0068 | 0.0370 | 0.0070 | -0.0990 |
|  | NAWM temporal pole R | -0.0731 | 0.0275 | 311 | -2.6628 | **0.0082** | -0.1270 | -0.0192 | 0.0395 | 0.0071 | 0.0390 | 0.0074 | -0.0731 |
|  | NAWM transverse temporal L | -0.0539 | 0.0174 | 309 | -3.0900 | **0.0022** | -0.0881 | -0.0197 | 0.0504 | 0.0107 | 0.0499 | 0.0107 | -0.0539 |
|  | NAWM transverse temporal R | -0.0691 | 0.0186 | 313 | -3.7128 | **0.0002** | -0.1057 | -0.0326 | 0.0369 | 0.0072 | 0.0364 | 0.0074 | -0.0691 |
| Parietal lobe | GM inferior parietal L | -0.0544 | 0.0090 | 313 | -6.0162 | **<0.0001** | -0.0721 | -0.0366 | 0.7770 | 0.1102 | 0.7712 | 0.1098 | -0.0544 |
|  | GM inferior parietal R | -0.0306 | 0.0102 | 314 | -2.9927 | **0.0030** | -0.0506 | -0.0105 | 0.9329 | 0.1155 | 0.9294 | 0.1158 | -0.0306 |
|  | GM isthmus cingulate L | -0.0371 | 0.0110 | 313 | -3.3613 | **0.0009** | -0.0588 | -0.0154 | 0.1654 | 0.0206 | 0.1647 | 0.0204 | -0.0371 |
|  | GM isthmus cingulate R | -0.0270 | 0.0114 | 312 | -2.3631 | **0.0187** | -0.0495 | -0.0046 | 0.1536 | 0.0212 | 0.1531 | 0.0211 | -0.0270 |
|  | GM postcentral L | -0.0294 | 0.0173 | 311 | -1.7008 | 0.0900 | -0.0634 | 0.0045 | 0.6390 | 0.0700 | 0.6372 | 0.0694 | -0.0294 |
|  | GM postcentral R | -0.0215 | 0.0179 | 312 | -1.2043 | 0.2294 | -0.0567 | 0.0136 | 0.6082 | 0.0693 | 0.6067 | 0.0695 | -0.0215 |
|  | GM posterior cingulate L | -0.0511 | 0.0112 | 312 | -4.5630 | **<0.0001** | -0.0730 | -0.0291 | 0.2000 | 0.0248 | 0.1988 | 0.0247 | -0.0511 |
|  | GM posterior cingulate R | -0.0295 | 0.0122 | 310 | -2.4243 | **0.0159** | -0.0534 | -0.0056 | 0.2021 | 0.0269 | 0.2014 | 0.0270 | -0.0295 |
|  | GM precuneus L | -0.0583 | 0.0136 | 314 | -4.3009 | **<0.0001** | -0.0849 | -0.0317 | 0.6268 | 0.0643 | 0.6231 | 0.0635 | -0.0583 |
|  | GM precuneus R | -0.0342 | 0.0140 | 313 | -2.4385 | **0.0153** | -0.0618 | -0.0067 | 0.6540 | 0.0638 | 0.6519 | 0.0635 | -0.0342 |
|  | GM superior parietal L | -0.0525 | 0.0151 | 314 | -3.4701 | **0.0006** | -0.0822 | -0.0228 | 0.8728 | 0.0974 | 0.8677 | 0.0974 | -0.0525 |
|  | GM superior parietal R | -0.0268 | 0.0160 | 313 | -1.6734 | 0.0953 | -0.0582 | 0.0046 | 0.8565 | 0.1005 | 0.8537 | 0.0992 | -0.0268 |
|  | GM supramarginal L | -0.0454 | 0.0113 | 313 | -4.0016 | **0.0001** | -0.0676 | -0.0231 | 0.7297 | 0.0912 | 0.7257 | 0.0891 | -0.0454 |
|  | GM supramarginal R | -0.0383 | 0.0112 | 312 | -3.4154 | **0.0007** | -0.0603 | -0.0163 | 0.6656 | 0.0806 | 0.6627 | 0.0813 | -0.0383 |
|  | NAWM inferior parietal L | -0.0560 | 0.0080 | 314 | -7.0107 | **<0.0001** | -0.0716 | -0.0403 | 0.5977 | 0.0870 | 0.5928 | 0.0892 | -0.0560 |
|  | NAWM inferior parietal R | -0.0392 | 0.0078 | 313 | -5.0385 | **<0.0001** | -0.0545 | -0.0239 | 0.6897 | 0.0892 | 0.6864 | 0.0901 | -0.0392 |
|  | NAWM isthmus cingulate L | -0.0775 | 0.0136 | 313 | -5.6997 | **<0.0001** | -0.1042 | -0.0508 | 0.2312 | 0.0284 | 0.2289 | 0.0294 | -0.0775 |
|  | NAWM isthmus cingulate R | -0.0824 | 0.0123 | 311 | -6.6934 | **<0.0001** | -0.1065 | -0.0582 | 0.2060 | 0.0267 | 0.2038 | 0.0278 | -0.0824 |
|  | NAWM postcentral L | -0.0666 | 0.0189 | 312 | -3.5125 | **0.0005** | -0.1037 | -0.0294 | 0.4456 | 0.0522 | 0.4428 | 0.0505 | -0.0666 |
|  | NAWM postcentral R | -0.0523 | 0.0194 | 313 | -2.6940 | **0.0074** | -0.0904 | -0.0142 | 0.4394 | 0.0472 | 0.4370 | 0.0489 | -0.0523 |
|  | NAWM posterior cingulate L | -0.0928 | 0.0131 | 313 | -7.0628 | **<0.0001** | -0.1186 | -0.0670 | 0.2733 | 0.0326 | 0.2703 | 0.0337 | -0.0928 |
|  | NAWM posterior cingulate R | -0.1095 | 0.0134 | 310 | -8.1935 | **<0.0001** | -0.1358 | -0.0833 | 0.2586 | 0.0318 | 0.2552 | 0.0333 | -0.1095 |
|  | NAWM precuneus L | -0.1048 | 0.0118 | 312 | -8.8600 | **<0.0001** | -0.1280 | -0.0816 | 0.5624 | 0.0727 | 0.5552 | 0.0735 | -0.1048 |
|  | NAWM precuneus R | -0.1076 | 0.0106 | 312 | -10.1784 | **<0.0001** | -0.1283 | -0.0868 | 0.5938 | 0.0792 | 0.5853 | 0.0802 | -0.1076 |
|  | NAWM superior parietal L | -0.0433 | 0.0106 | 312 | -4.0786 | **0.0001** | -0.0641 | -0.0225 | 0.7466 | 0.0825 | 0.7430 | 0.0841 | -0.0433 |
|  | NAWM superior parietal R | -0.0317 | 0.0091 | 314 | -3.4804 | **0.0006** | -0.0496 | -0.0138 | 0.7215 | 0.0823 | 0.7188 | 0.0839 | -0.0317 |
|  | NAWM supramarginal L | -0.0226 | 0.0091 | 313 | -2.4920 | **0.0132** | -0.0404 | -0.0048 | 0.5533 | 0.0720 | 0.5516 | 0.0727 | -0.0226 |
|  | NAWM supramarginal R | -0.0258 | 0.0080 | 313 | -3.2215 | **0.0014** | -0.0415 | -0.0101 | 0.5375 | 0.0692 | 0.5358 | 0.0710 | -0.0258 |
| Occipital lobe | GM cuneus L | -0.0329 | 0.0094 | 314 | -3.5176 | **0.0005** | -0.0513 | -0.0145 | 0.2035 | 0.0356 | 0.2023 | 0.0349 | -0.0329 |
|  | GM cuneus R | -0.0390 | 0.0112 | 314 | -3.4708 | **0.0006** | -0.0610 | -0.0169 | 0.2238 | 0.0330 | 0.2225 | 0.0326 | -0.0390 |
|  | GM lateral occipital L | -0.0631 | 0.0140 | 312 | -4.5204 | **<0.0001** | -0.0906 | -0.0357 | 0.8060 | 0.0890 | 0.8006 | 0.0886 | -0.0631 |
|  | GM lateral occipital R | -0.0269 | 0.0120 | 313 | -2.2446 | **0.0255** | -0.0504 | -0.0034 | 0.8294 | 0.0999 | 0.8268 | 0.0974 | -0.0269 |
|  | GM lingual L | -0.0405 | 0.0105 | 311 | -3.8496 | **0.0001** | -0.0611 | -0.0198 | 0.4309 | 0.0595 | 0.4286 | 0.0587 | -0.0405 |
|  | GM lingual R | -0.0361 | 0.0101 | 314 | -3.5790 | **0.0004** | -0.0559 | -0.0163 | 0.4607 | 0.0625 | 0.4584 | 0.0615 | -0.0361 |
|  | GM pericalcarine L | -0.0310 | 0.0138 | 311 | -2.2411 | **0.0257** | -0.0581 | -0.0038 | 0.1378 | 0.0269 | 0.1371 | 0.0268 | -0.0310 |
|  | GM pericalcarine R | -0.0441 | 0.0146 | 312 | -3.0180 | **0.0028** | -0.0729 | -0.0154 | 0.1550 | 0.0277 | 0.1538 | 0.0272 | -0.0441 |
|  | NAWM cuneus L | -0.0331 | 0.0137 | 311 | -2.4145 | **0.0163** | -0.0599 | -0.0062 | 0.1586 | 0.0278 | 0.1579 | 0.0278 | -0.0331 |
|  | NAWM cuneus R | -0.0030 | 0.0145 | 311 | -0.2043 | 0.8383 | -0.0315 | 0.0256 | 0.1623 | 0.0278 | 0.1623 | 0.0289 | -0.0030 |
|  | NAWM lateral occipital L | -0.0689 | 0.0143 | 312 | -4.7993 | **<0.0001** | -0.0970 | -0.0407 | 0.6024 | 0.0727 | 0.5976 | 0.0723 | -0.0689 |
|  | NAWM lateral occipital R | -0.0468 | 0.0115 | 312 | -4.0644 | **0.0001** | -0.0694 | -0.0242 | 0.6219 | 0.0824 | 0.6184 | 0.0829 | -0.0468 |
|  | NAWM lingual L | -0.1169 | 0.0151 | 312 | -7.7652 | **<0.0001** | -0.1464 | -0.0873 | 0.3336 | 0.0478 | 0.3283 | 0.0476 | -0.1169 |
|  | NAWM lingual R | -0.0787 | 0.0132 | 313 | -5.9618 | **<0.0001** | -0.1047 | -0.0528 | 0.3518 | 0.0589 | 0.3472 | 0.0608 | -0.0787 |
|  | NAWM pericalcarine L | -0.1607 | 0.0127 | 313 | -12.6755 | **<0.0001** | -0.1855 | -0.1358 | 0.1960 | 0.0442 | 0.1891 | 0.0441 | -0.1607 |
|  | NAWM pericalcarine R | -0.1376 | 0.0120 | 312 | -11.5135 | **<0.0001** | -0.1610 | -0.1141 | 0.2039 | 0.0450 | 0.1978 | 0.0462 | -0.1376 |
| Insula | GM insula L | -0.0484 | 0.0169 | 311 | -2.8575 | **0.0046** | -0.0817 | -0.0152 | 0.4547 | 0.0400 | 0.4528 | 0.0395 | -0.0484 |
|  | GM insula R | -0.0420 | 0.0190 | 313 | -2.2143 | **0.0275** | -0.0792 | -0.0048 | 0.4491 | 0.0374 | 0.4476 | 0.0382 | -0.0420 |
|  | NAWM insula L | -0.0386 | 0.0144 | 311 | -2.6743 | **0.0079** | -0.0669 | -0.0103 | 0.6146 | 0.0534 | 0.6129 | 0.0522 | -0.0386 |
|  | NAWM insula R | -0.0185 | 0.0183 | 312 | -1.0103 | 0.3131 | -0.0544 | 0.0174 | 0.5967 | 0.0493 | 0.5958 | 0.0487 | -0.0185 |

B=standardised beta value, SE=standard error, df=degrees of freedom, CI=confidence interval for beta value, SD=standard deviation, w0=baseline, w1=1-year follow-up, L=left, R=right, GM=grey matter, NAWM=normal-appearing white matter, sts= superior temporal sulcus, WML=white matter lesion, DMT=disease-modifying treatment. p-values surviving FDR correction (q<0.05) are highlighted in bold

^a^This model included the significant interaction term for time*age. See table S3.
